# Occupational exposure to asbestos in the steel industry (1972–2006)

**DOI:** 10.1038/s41370-023-00576-4

**Published:** 2023-07-26

**Authors:** Dennis J. Paustenbach, Michael E. Stevens, Brett P. Tuttle, Ross A. Shore, Sabina Ligas, David W. Brew

**Affiliations:** Paustenbach and Associates, 970 W Broadway, Suite E, Jackson, WY 83001 USA

**Keywords:** Risk assessment, Steel mill workers, Asbestos, Exposure assessment, Industrial hygiene

## Abstract

**Background:**

Historically, the use of asbestos in steelmaking has been limited to a few applications. Due to its physical and chemical properties, asbestos was not necessary or suitable for most purposes in a steel mill. The few applications where asbestos were used (i.e., certain gaskets, brakes, protective cloth, refractory materials, insulation materials, and hot top products) were replaced by alternative materials as they became available.

**Objective:**

We discuss historical uses of asbestos in steel manufacturing and the associated airborne asbestos concentrations collected at sixteen U. S. Steel facilities between 1972 and 2006.

**Methods:**

A total of 495 personal airborne asbestos samples from the U. S. Steel industrial hygiene records were analyzed across four time periods corresponding to changes in the OSHA permissible exposure limit (PEL) for asbestos. 68% of the samples (*n* = 337) were considered representative of an employee’s workday. The remaining samples (*n* = 158) represented task samples. Samples were grouped by facility, department, and job category within the four time periods.

**Results:**

The average fiber concentrations measured for each facility and department over time were below the contemporaneous OSHA PEL. The mean representative workday asbestos air concentration from 1972 and 1975 was 1.09 f/cc. The mean representative workday concentration decreased to 0.13 f/cc between 1976 and 1985, then decreased again to 0.02 f/cc between 1986 and 1993 and 0.03 f/cc between 1994 and 2006. For task samples, the mean air concentration from 1972 to 1975 was 3.29 f/cc. The mean task sample concentration decreased to 0.48 f/cc between 1976 and 1985, then decreased again to 0.01 f/cc between 1986 and 1993 and 0.03 f/cc between 1994 and 2006. Only eleven out of the 495 samples (2.2%), for both task and representative workday samples, were in exceedance of the contemporaneous PEL(as an 8-hour TWA), ten of which occurred prior to 1978. Eight of these eleven PEL exceeding samples were task samples. Of the remaining three representative workday samples, two had unknown sampling times.

**Impact:**

- This paper presents an analysis of all the available personal sampling data for airborne asbestos across 16 facilities of the U. S. Steel Corporation between 1972 and 2006. This dataset has previously never been publicly shared or analyzed. It represents one of the more complete industrial hygiene datasets from a corporation to be presented in a scientific journal and, due to the similarities in the processes at each mill, it should reflect analogous exposures throughout the steelmaking industry in the United States. One of the benefits of presenting these data is that it also provides insight into where asbestos-containing materials (ACMs) were used in the steel making process. This is just one example of a large firm that released information that had previously remained in file cabinets for decades. We believe that another benefit of publishing this paper is that it may encourage the largest firms in industry to assemble and analyze their industrial hygiene data to benefit the occupational hygiene, medical, and epidemiology communities. This can support future epidemiology studies and improve the design of future industrial hygiene programs.

## Introduction

Historically, economic volatility and global outsourcing have impacted the American steel industry. In the 1950s, the United States produced approximately half of the world’s steel, but by 1984, its share had dropped to below 12% of the global steel output [1]. Between 1950 and 1984, approximately 100 million tons of steel were produced in the United States annually [1].

As was the case for workers in most major manufacturing industries in the pre-OSHA era, some steelworkers were occasionally, if not routinely, exposed to physical and chemical hazards in their work environments. Physical hazards in steel mills included noise, vibration, airborne dusts, gases, heat stress, injuries from equipment, cuts, and burns [2]. An analysis of historical data in the steel industry indicates that various dusts, silica, asbestos, lead, carbon monoxide, coke oven emissions, and chlorinated solvents were worthy of evaluation [2].

At U. S. Steel and other major steel companies in both the pre-OSHA (pre-1970) and post-OSHA eras (1970–present), the inhalation hazards in the steel mills were generally well characterized and up to date with contemporaneous knowledge [2]. Occupational exposures to asbestos were studied beginning in the mid-1960s by U. S. Steel after adverse effects were recognized in other industries [3, 4].

Contrary to the perception of some outside the steel industry that asbestos was extensively used during various steel making processes from the 1940s to the 1980s; there was actually a limited use of asbestos in the various high-temperature operations because the intense heat caused asbestos to degrade. Unless this degradation was desired (i.e., hot topping and expansion sheets), asbestos was not sought after for these high-temperature purposes. In general, there were limited instances in which workers could be exposed to asbestos in the steel industry. As addressed in this paper, historical exposures to asbestos in steel mills could have included working with asbestos-containing gaskets, brakes, protective cloth, select refractory materials, insulation materials, and hot top products. However, the available data supports that exposures associated with these activities typically did not exceed the contemporaneous occupational exposure limits (OELs).

The objective of this paper was to evaluate the historical magnitude of asbestos exposures to workers in the U. S. Steel facilities and departments from 1972 through 2006 by comparing multiple exposure groups to one another, as well as to the OELs over time. As will be discussed, the decrease in potential asbestos exposures over time follow changes in facility and hygiene practices, including the decreased use of asbestos-containing materials (ACMs) at U. S. Steel. This is the most robust known collection of industrial hygiene data regarding exposure to asbestos within the steelmaking industry. In our view, the information presented characterizes not only the likely range of exposures at U. S. Steel facilities, but also represents possible asbestos exposures in other major steel companies within the United States during this era since steel is manufactured essentially the same way across the industry. Other companies where the data should be insightful include Bethlehem, Republic, Armco, Jones & Laughlin, Inland, Allegheny Ludlum, Wheeling-Pittsburgh and more. Regardless of the company, analogous production processes involving similar equipment, departments, and plant staff were utilized [5, 6].

## Background

### Asbestos regulation and U. S. Steel

Medical, industrial hygiene, and safety departments at U. S. Steel collectively developed safe workplace programs. Those hired to run the industrial hygiene and medical departments were often leaders in the fields of occupational health. Discussions with many other professionals in the steel industry, experts in occupational health [7], and professional societies aided in developing the practices at U. S. Steel. Due in part to the steel industry union, the United Steelworkers of America, and trade associations, like the American Iron and Steel Institute, steel making companies compared themselves to one another, especially in regards to workplace safety (personal communications with Dr. Fred Toca). U. S. Steel’s efforts often served as exemplars for regulatory bodies including the Occupational Safety and Health Administration (OSHA), the National Institute for Occupational Safety and Health (NIOSH), and the Environmental Protection Agency (EPA) [8].

U. S. Steel relied on recommendations by government and professional societies (i.e., the American Conference of Governmental Industrial Hygienists (ACGIH) threshold limit values (TLVs)) to identify acceptable levels of exposure to various toxic substances. U. S. Steel was responsive to industry and governmental recommendations concerning employee exposure to occupational hazards, including asbestos, as it became clear in the early 1960s that asbestos was an important occupational health hazard in American industry [3].

On December 7th, 1971, OSHA published an “Emergency Standard for Exposure to Asbestos Dust” which established a permissible exposure limit (PEL) of 5 fibers per cubic centimeter (f/cc) as an 8-hour time-weighted average (TWA) and a peak exposure level of 10 f/cc as a 15-minute ceiling [9]. In response, U. S. Steel’s Director of Environmental Health, Mr. Ken Morse, sent an inter-organizational letter on December 10th, 1971, [10] to the general superintendents, plant managers, superintendents of personnel services, and safety supervisors, which stated:

“The above regulations and the hazards associated with asbestos dust warrants immediate attention to determine those occupations in your facilities which utilized asbestos in any form and in determining the concentrations to which workers may be exposed” [10].

This is one of the numerous examples of U. S. Steel’s response to proposed or promulgated government regulations. On July 3rd, 1972, in response to the promulgation of OSHA’s asbestos PEL, Mr. Ken Morse issued a *Proposed Permanent Asbestos Standard for U. S. Steel* [11]. This standard described the methods of compliance to be used in U. S. Steel’s facilities, including (a) engineering controls (i.e., local exhaust ventilation), (b) work practices (i.e., wetting of all materials that contained asbestos prior to manipulation and wearing U.S. Bureau of Mines and NIOSH-approved respirators and special clothing when employees sprayed asbestos and removed/demolished pipes, structures, or equipment insulated with asbestos), (c) the monitoring requirement (to determine the exposure of every employee and then repeated at least every six months for employees who were expected to be exposed), (d) the use of caution signs in areas where asbestos is used and labeling of ACMs (except for those where a bonding agent is used), (e) housekeeping (i.e., appropriate cleaning and waste disposal), (f) recordkeeping, and (g) medical examinations (required for all U. S. Steel employees within 30 days of their first exposure to asbestos).

In 1972, when OSHA instituted requirements for air monitoring for asbestos, U. S. Steel had initiated a program to train personnel in how to conduct air monitoring [12]. This became *The Environmental Health Monitoring Manual*, which was developed to help with training on silica and other contaminants, including asbestos [13]. In 1980, based on U. S. Steel’s experience identifying and measuring asbestos in their facilities, the EPA requested U. S. Steel’s assistance in creating guidelines for setting up laboratories, equipment, and personnel capable of analyzing particulates, including asbestos fibers, in order to establish guidelines for release into the ambient air [8].

### Removal of asbestos by abatement firms at U. S. Steel facilities

After the early 1980s, licensed abatement workers, who would not have been U. S. Steel employees, were commonly utilized to remove and dispose of asbestos-containing insulation [14]. During the remediation process, these contracted workers would build standard plastic enclosures around the section(s) of the facility that they were abating to prevent asbestos fibers from traveling beyond the containment area [15]. These enclosures were maintained under negative air pressure to further prevent airborne asbestos fibers from leaving the enclosures [15]. Any ACMs were to be removed from the containment area in sealed bags [16–19]. To ensure proper containment, industrial hygienists at U. S. Steel monitored the perimeter of the enclosures by collecting area air samples [20–26]. The areas of the facility where the abatement process took place were only returned to U. S. Steel employees once testing showed that asbestos concentrations were well below the contemporaneous OSHA PEL [27].

Federal, along with state governments, regulated how asbestos abatement companies needed to operate, train, and protect their employees. According to internal U. S. Steel reports, the expected safety precautions regarding asbestos abatement were to be understood and followed by the abatement contractors while they performed their work. The abatement workers inside the containment areas were required to wear respirators, neoprene gloves, goggles, boot covers, and Tyvek coveralls or suits [18, 19, 28, 29].

### Types of asbestos fibers

OSHA regulates all types of asbestos under the same occupational exposure limit, yet it is important to note the distinction between chrysotile, amosite, and crocidolite when characterizing human health risks [30]. Chrysotile is the only form of serpentine asbestos and has historically accounted for more than 95% of all asbestos used around the world [31, p. 893]. The remaining percentage is comprised of amphibole asbestos, which predominately includes anthophyllite, amosite, crocidolite, actinolite, and tremolite [32]. Apart from rare instances of trace contaminants, only amosite and crocidolite amphiboles were used commercially. Each of these fibers has different chemical structures, iron content, solubilities in lung tissue fluid, and potencies for causing adverse effects [33].

It has been postulated since the early 1960s and known since the late 1970s that there are substantial potency differences between chrysotile asbestos and amphibole fibers for both lung cancer and mesothelioma [33–36]. Compared to chrysotile fibers, amphiboles are significantly more resistant to being degraded by macrophages and are much more persistent in the lungs [33, 37–39]. Studies indicate that chrysotile fibers, when exposed to the acidic conditions of a macrophage, are readily depleted of magnesium and other cations, which facilitates the breakdown of fiber bundles and lung clearance [37, 38]. The biological half-life of inhaled amphiboles ranges from years to decades, whereas the half-life of chrysotile is only days to weeks [32, 40, 41]. Many animal and human studies have shown that exposure to amphibole fibers represent a much greater hazard, compared to chrysotile, in causing an asbestos-related disease [34, 42–45].

Pierce et al. [46] reported that the “best estimate” for the no-observed-adverse-effect level for chrysotile to cause mesothelioma was 208 to 415 f/cc-years, and to cause lung cancer was 89 to 168 f/cc-years [46]. There is significant evidence that, if it does cause mesothelioma, chrysotile asbestos requires doses that are likely at least 200 f/cc-years, with fibers that are longer, likely greater than 25 μm in length, and have an aspect ratio greater than 3:1 [34, 45–47]. In other words, one has to have experienced doses that can cause asbestosis for chrysotile to be a mesothelioma hazard.

Some authors have suggested that chrysotile exposure may not be a risk factor for mesothelioma at all. Berman and Crump stated that based upon the exposure-response modeling of epidemiology studies that for “mesothelioma the best estimate of the coefficient (potency) for chrysotile is only 0.0013 times that for amphibole and the possibility that pure chrysotile is non-potent for causing mesothelioma cannot be ruled out by the epidemiology data” [45].

All types of commercial asbestos decompose at 1000 °C (1832 °F), or lower depending on the mineral species [48]. When heated to temperatures above 800 °C (1472 °F), chrysotile asbestos “survives for only minutes,” and is degraded to forsterite (a non-asbestos, non-toxic magnesium silicate) [49–52]. The two most common amphiboles, amosite and crocidolite, degrade into silicates at approximately 900 °C (1652 °F) and 930 °C (1706 °F), respectively [52].

### Historical asbestos-containing materials (ACM) in the steel industry

We assembled the available U. S. Steel industrial hygiene reports from 1972 to 2006 and have consulted with several former steel mill employees from the 1960s to 1990s era to confirm our understanding of the potential asbestos exposures in the industry. Based on our research, it is reasonable to assume that the same ACMs were used in virtually all steel mills throughout the time period considered in this paper. As will be discussed later, few of these ACMs were routinely manipulated and, when they were, the direct worker exposures were usually below the contemporaneous OSHA PEL.

U. S. Steel began to routinely evaluate concentrations of airborne asbestos that were generated through interactions with ACMs in 1965 utilizing an area sampling method [4]. As part of U. S. Steel’s early attempts to understand the possible use of asbestos in their facilities, they conducted inventories of ACMs. A memorandum dated October 27th, 1970, provided this inventory of the ACMs in various facilities [53]. Industrial hygiene reports in 1973 and later recommended to U. S. Steel management that alternatives to ACMs should be purchased [12, 54]. As monitoring of ACMs continued, a report in 1977 from the Fairless Works facility detailed the ACMs used in each department [54]. It was reported that by 1977, the active new use of most ACMs (i.e., cement, insulation block, expansion sheets [asbestos paper], hot top liners and boards, gaskets, and mortar) had been substantially reduced or ceased [54].

### Possible opportunities for exposure in the steel industry

In contrast to some anecdotal claims, objective analysis suggests that from approximately the 1940s through the present day, worker exposure to asbestos in the steelmaking industry has been infrequent and not widespread. The following are the most plausible opportunities for exposure to ACMs in steel mills, which were mostly comprised of chrysotile asbestos. ACMs used in the steelmaking process included gaskets, brakes, protective cloth, refractory materials, insulation materials, and hot top products.

### Replacing gaskets

From approximately the 1940s through the early 1980s, gaskets, including rope gaskets, and packing materials for valve stems used in the steelmaking industry, if asbestos-containing, contained exclusively chrysotile [55–58].

Products containing encapsulated asbestos fibers (i.e., gaskets, phenolic molded materials, etc.) have generally been known to pose a negligible health risk and have therefore been historically and presently exempt from federal asbestos labeling requirements [59–62]. Encapsulated means that the asbestos is bound in a resin, paper, glues, or other media; asbestos is generally used as a filler, and the asbestos is almost impossible to release into the air without significant manipulation (e.g., cutting with a saw or an electric sander).

Gaskets that contained asbestos, and most did not, normally contained between 40% and 80% chrysotile asbestos by weight [55]. Published studies for the relevant time periods indicate that 8-hour TWA exposures were generally below the current OSHA PEL of 0.1 f/cc, whether the worker was directly exposed [55, 58, 63, 64] or indirectly as a bystander [65, 66]. Madl et al. [58] reviewed seven simulation studies and four work-site studies containing over 300 air samples for employees working with encapsulated asbestos-containing gaskets and packing materials [58]. The average concentration of asbestos fibers was 0.09 f/cc for samples collected (typically collected for approximately 30 minutes) during all gasket removal and installation activities (*n* = 58) [58]. In all but one of the studies involving the replacement of gaskets and packing using hand-held tools, the short-term average exposures were less than the current 30-minute OSHA excursion limit of 1 f/cc and all the long-term average exposures were less than the current 8-hour OSHA PEL-TWA of 0.1 f/cc [58]. The authors concluded that the use of hand tools and hand-operated power tools to remove or install gaskets or packing should not, under conditions normally encountered, have produced airborne concentrations in excess of contemporaneous regulatory levels [58].

For rope gaskets, which were sometimes used in steel mills around the doors of coke ovens, U. S. Steel industrial hygienists found that asbestos exposure from these products were below the PEL or the limit of detection (LOD) [67–69]. It should be noted that as of 1972, U. S. Steel’s policy was that all ACMs, including gaskets, were to be wet when they were handled or cut [11, 70]. By the time rope gaskets were removed, chrysotile asbestos in the gaskets would be degraded to a harmless dust (e.g., forsterite). This is because coke ovens are held between 1000 °C (1832 °F) and 1100 °C (2012 °F), which is between 200 and 300 degrees above the temperatures in which chrysotile asbestos degrades [71].

### Replacing brake pads

Regarding the use of brake pads in steel mills, industrial hygienists primarily evaluated conveyor and crane brakes [72, 73]. Replacing brakes on these machines is an infrequent event. Historically, chrysotile was the only form of asbestos used in brake pads [74–76]. By the 1980s, brake pads for cranes, vehicles, conveyors, and other applications began to become asbestos-free [75]. This was a gradual phase out period as it was difficult to identify a replacement material for asbestos that made manufacturers confident brakes would perform adequately under a wide array of temperatures and conditions critical for ensuring safety. As was the case with all ACMs at U. S. Steel, asbestos-containing brakes were to be wet when handle after 1972 [11, 77].

Paustenbach et al. performed a review of the available published literature for brake mechanics exposed to asbestos [78]. From 1968 through 1996, among 141 automobile mechanics who performed brake jobs, the authors reported an average 8-hour TWA of 0.05 f/cc (min: 0.004 f/cc; max: 0.28 f/cc). Among 162 car and light truck personal samples on mechanics that performed brake repairs, they reported an average 8-hour TWA of 0.04 f/cc (min: 0.00; max: 0.68 f/cc). Among automobile mechanics in the 1970s, the cumulative distribution of 8-hour TWA concentrations had a 50th percentile of 0.07 f/cc and 90th percentile of 0.10 f/cc. In the 1980s, the reported cumulative 8-hour TWA concentrations were significantly lower than those from the prior decade and had a 90th percentile of 0.002 f/cc [78]. An internal EPA memoranda supports 0.04 f/cc as a reasonable estimate of the exposure of workers performing brake work before 1985 [79].

The brake wear debris for automobiles was nearly the same as for crane and conveyer brakes. Sahmel et al. performed an analysis on chrysotile asbestos exposures during crane operation as well as crane brake and clutch maintenance [80]. For the two 30-minute crane operation samples, transmission electron microscopy (TEM) analysis of the only sample that detected fibers quantified one chrysotile and one non-asbestos fiber [80, p. 12]. This resulted in an average chrysotile concentration of 0.005 f/cc (PCME) [80, p. 12]. Short-term exposures (i.e., 15-to-36-minute sampling times) for brake maintenance which includes lining removal, installation, wire brushing, hand sanding, and compressed air use had concentrations that ranged from non-detectable to 0.238 f/cc, which is below the current OSHA 30-minute excursion limit of 1 f/cc [80].

In 1984, two U. S. Steel industrial hygiene reports examined crane brake pads and detected that the pads contained between 20% and 40% chrysotile asbestos upon installation [81, 82]. A 1985 industrial hygiene report at U. S. Steel found greater than 50% chrysotile in a bulk sample of a conveyor brake pad [83]. None of these reports detected any amphibole asbestos in these brake pads. U. S. Steel industrial hygiene reports in 1984 and 1985 found the dust from these crane and conveyor brake pads following normal use contained less than 1% chrysotile asbestos fibers [81, 83]. These results are consistent with many other studies of brakes as the wear debris from encapsulated chrysotile in brakes is degraded mechanically and thermally [74, 79, 80, 84–91].

### Protective cloth and clothing used in hot environments

Historically, asbestos blankets have been used to prevent sparks and heat from affecting welders, welding surfaces, and equipment [14]. Certain employees in hot environments, such as the blast furnace and melt shop, may have routinely worn protective gloves, mittens, and clothing which protected them from heat and hot products [54, 77, 92]. According to a 1978 industrial hygiene report at U. S. Steel, asbestos blankets were rarely, if ever, used by maintenance and coke personnel; however, they were reportedly used in blast furnace operations [70]. In 1977, a U. S. Steel industrial hygiene report stated that the finishing department’s use of asbestos was limited; however, in the rolling division, scarfers and scalemen were stated to have utilized asbestos cloth to insulate hoses on scarfing units or as a curtain material [54].

Protective clothing, used for safety in extreme heat environments, until the 1980s may have contained chrysotile asbestos due to its ability to be woven into fabrics easily. The hard, needle-like properties of amphibole fibers are poorly suited for weaving into cloth and clothing. However, in limited applications, blanket material in shipyards were reported to have contained amosite [93, 94].

In most cases, asbestos textile materials were treated (bound or coated with resins or elastomers prior to being manufactured) into finished products (i.e., welding curtains, draperies, blankets, protective clothing, hot conveyor belts, furnace shields, and molten metal splash protection aprons [95, p. 270].

Under typical conditions of use, the opportunity for fiber release from asbestos-containing cloth is *de minimis* and the airborne concentrations are usually immeasurably low [77, 96]. As described in a 1977 industrial hygiene report, OSHA did not evaluate the potential exposure involved in the use of asbestos protective clothing because “… it would not appear necessary to include such potential exposure in either the asbestos product inventory, or in further air sampling surveys” [54, p. 4]. According to a 2005 study, “people who wore asbestos mitts were likely to have been exposed to relatively low levels of airborne chrysotile asbestos fibers, certainly much lower than the standards that were accepted in the 1960s and [19]70s. The cancer risks from this type of use are likely to be very low” [97]. In Cherrie et al. the measured respirable fiber exposure from the use of asbestos-containing mitts ranged from less than 0.060 f/cc to 0.55 f/cc, with no signifigant difference in exposures between aged and unused mitts [97]. Longo and Hatfield found similarly low personal asbestos air concentrations during the use of asbestos-containing gloves with a mean concentration of 0.023 f/cc (lower limit: 0.019 f/cc; upper limit: 0.027 f/cc) [96].

Specific to U. S. Steel, at their Homestead Works facility, samples were collected for hot shear stampers whom the industrial hygienist believed had no source of potential asbestos exposure other than the asbestos gloves and protective clothing that they were wearing [92]. This sampling indicated a short-term air concentration of 0.05 f/cc [92]. In the same report, air samples on a scarfer in the 45” Mill, who’s only believed exposure to asbestos was from wearing asbestos-containing clothing, indicated an 8-hour TWA of 0.08 f/cc [92].

### Replacing refractory materials

Refractory materials have been characterized as substances that can withstand high heat and mechanical loads while maintaining their function. The use of the correct type of materials is important in the steel manufacturing industry for safe and efficient process control. Refractory materials are the primary materials that are used in retaining vessels and furnaces, and they are also used in the conduction of hot gases through flues and stacks [71, Chapter 3].

Iron melts at temperatures of approximately 2800 °F [71] and steel, being an alloy of iron, melts at temperatures slightly below 2800 °F. As such, melting vessels such as electric arc furnaces, basic oxygen blast furnaces, and open-hearth furnaces all operate at temperatures around 3000 °F [71]. As mentioned previously, these temperatures would degrade all forms of asbestos into harmless silicates [48, 52].

Asbestos degradation under these temperatures makes asbestos-containing cement structurally unsuitable for refractory use in melting vessels. For this reason, asbestos-free cements like *Johns-Manville No. 460 Insulating cement* were used at U. S. Steel facilities, according to a 1974 industrial hygiene report [98]. According to inventory reports from 1972, U. S. Steel did not use asbestos-containing mortar [54]. Applications of asbestos-containing cement were typically non-refractory applications such as pipe and external boiler insulation.

Masons or bricklayers may have used asbestos paper (expansion sheets) in open-hearth roofs and the sidewalls of some furnaces and stoves [5]. It is a common misconception that refractory brick contained asbestos [99]. After thermal decomposition, asbestos would impair the structural features of the aluminum silicate clay refractory brick materials. Refractory brick was asbestos-free, except for metal-clad bricks (sometimes used for open-hearth roofs) that came pre-packaged with both the clay brick and asbestos paper wrapped in a metal case [14, 99, 100]. Pre-encased asbestos was within the metal case and presented no risk to installation employees (e.g., masons and bricklayers).

If asbestos-containing, paper expansion sheets contained chrysotile [100–102]. Sheets of this asbestos paper were inserted in between every 5–10 refractory bricks during the installation of open-hearth roof linings and sidewalls of some steel melting vessels such as furnaces or stoves for expansion allowance [5]. This paper could have been cut onsite but commonly came pre-cut [100]. This asbestos paper would decompose immediately after the ovens were fired, which is what allowed for expansion of refractory bricks upon heating to extreme temperatures [100]. This means that when removing these bricks for repairs or replacement, there would be no potential for generating airborne asbestos as any asbestos from the paper would have been converted to silicates. Hollins et al. [100] found TWA airborne asbestos concentrations, when sampled for greater than 227 minutes during refractory removal activities, averaged 0.045 f/cc. Bradt found TWA airborne asbestos concentrations between 0.1 f/cc and 0.3 f/cc amongst refractory removal activities or jobs in 1970 [99]. Forsterite or other non-asbestos fibers, which can be indistinguishable from asbestos by phase contrast microscopy (PCM) analysis (discussed further in the *Limitations of PCM Sampling* section), likely contributed to these concentrations.

### Replacing insulation materials

Pipe insulation installed before the mid-1970s, if it was asbestos-containing, could have contained both chrysotile and/or amphibole asbestos [103]. The presence of both asbestos fiber types was confirmed in a bulk sampling analysis performed on insulating material that was knocked off an overhead pipe in one of the finishing department offices at Fairfield Works [104]. This sample identified that the material composition was 3% chrysotile, 20% amphibole and 77% binder [104]. It could not be determined from the industrial hygiene report when this pipe insulation was initially installed. The asbestos type and composition of pipe insulation was not identified in any other available industrial hygiene reports. After the mid-1970s, fiberglass and non-asbestos insulation materials were widely adopted in American industry, including the chemical and steel industries [14].

In 1977, according to a U. S. Steel industrial hygiene report, pipefitters in the maintenance department and boiler house performed repair work with pipe insulation that was potentially asbestos-containing [54]. Insulation block, half-round, waterproof adhesive, cloth, and non-refractory cement were possible ACMs used for pipe insulation [105, 106]. Insulation block also was documented in an industrial hygiene report to have been used when relining soaking pits [107].

It is known that most interactions with asbestos-containing pipe insulation occurred during the construction of steel mill facilities and that this construction was performed by large construction companies and not steel mill employees (personal communications with Dr. Fred Toca). Because the steelmaking processes rarely changed after initial construction, the potential interactions with pipe insulation were generally limited to repairs. Repairs of pipe insulation were infrequent and due mostly to accidental damage. The insulation on a boiler, after it has been installed during construction, would typically never need to be replaced during the lifespan of the boiler.

The possible use of non-refractory asbestos-containing cement in a steel mill was generally limited to insulating pipes as well as being a component in cement insulation boards. In 1972, asbestos-containing insulating cement typically contained 15% or less chrysotile asbestos [108, p. 175]. Insulation board was used in some instances in steel manufacturing facilities to build walls, benches, and huts [100]. It was also reported to have been installed in overhead crane cabs that were used in the steel-pouring process to protect the crane operator from radiant heat [109–111]. The asbestos in insulation boards was encapsulated (i.e., non-friable) and thus did not pose an inhalational hazard to employees [100, 112]. With respect to the term “encapsulation”, OSHA has defined it in the following manner [113]:

“When cements, mastics, coatings, and flashings are manufactured and installed, the asbestos fibers are tightly encapsulated by adhesive bituminous and resinous compounds that effectively prevent the fibers from being released. In order to provide effective waterproofing, these materials must retain their adhesive quality over their useful life. Accordingly, when such materials are intact prior to removal, the use of commonly used manual methods to remove the material will not result in significant fiber release.”

There should have been no measurable exposure of workers in most instances as these boards were pre-cut, so it was not necessary for maintenance to saw them. Air sampling of crane cabs with insulation boards shows no measurable exposure [114, 115]. An OSHA study concluded that drilling and cutting during “asbestos-containing sheet installation” was associated with an 8-hour TWA ranging from non-detectable levels (below 0.1 f/cc) to 0.50 f/cc with geometric means of around 0.1 f/cc [116].

In a 1972 U. S. Steel industrial hygiene report, the wetting of insulation materials prior to removal was required [117]. This was recommended in the 1972 OSHA guideline [60]. A 1981 report confirmed that this practice was also used with insulation board [111]. Additionally, U. S. Steel instituted rules in 1972 that required employees involved in “… the removal or demolition of asbestos insulation or coverings shall be provided with respiratory equipment … and with special clothing …” [118].

A 1980 U. S. Steel industrial hygiene report explained that when employees remove insulation materials they were to follow these guidelines: (a) wet materials prior to handling, (b) wear approved and properly selected respirators, (c) minimize the amount of fracturing of the insulation, (d) avoid using power tools, (e) seal the insulation in impermeable bags, (f) restrict employees from the area who are not involved in the process, and (g) that breathing zone samples of these employees should be collected [119]. Following the removal process, employees were to wash their hands, face, and forearms, their clothing was to be properly cleaned, the area was to be cleaned without the use of dry sweeping, and disposal of the ACM filled bags was to be completed by an outside contracted company [119]. Employees removing insulation were expected to be “… instructed to the hazards of asbestos and the precautionary measures that are being taken to control their exposures” [119, p. 3]. After the early 1980s, shortly following this report, the removal of potential asbestos-containing insulation was performed by licensed outside abatement workers [14].

### Handling hot tops and cleaning ingot molds

Hot tops are metal castings placed at the top of ingot molds prior to ingot teeming (steel being poured from a handle into molds). Historically, there were various manufacturers and styles of hot tops, including those lined with one-time-use boards/liners made from cellulose, resin, asbestos, and sand [120, 121]. Hot tops would be placed on top of ingot molds to control the cooling of the steel [5]. These boards could also be placed directly into ingot molds, and these served the same purpose as hot tops (personal communications with Dr. Fred Toca). The application of a hot top system or just the board allowed the steel to cool more slowly, which allowed impurities in the steel to rise and improved the quality of the finished steel. Hot topping additionally limits the formation of pipe (the shrinkage cavity at the top of ingots that occurs during cooling) and blowholes (deeper cavities) from traveling down the ingot [5, p. 394–395]. Limiting the formation of pipe and blowholes further improves the quality of steel.

Ferro hot top liners and Foseco hot top boards were mentioned as being used at U. S. Steel facilities in the available industrial hygiene reports [54, 122]. Historically, certain Ferro hot top liners contained a mixture of 6% asbestos (chrysotile and amosite) [121]. Ferro hot top liners were labeled as asbestos-containing in 1972 and were asbestos-free by mid-1974 [121].

Asbestos composition varied across Foseco products, however many Foseco products were asbestos-free by 1972, and all were reportedly asbestos-free by July 1976 [123, p. 50]. All asbestos-containing Foseco products were labeled from 1972 to 1976, like most asbestos products, with the following text as mandated by OSHA [123, p. 74]:

CAUTION

CONTAINS ASBESTOS FIBERS

AVOID CREATING DUST

BREATHING ASBESTOS DUST MAY CAUSE BODILY HARM

In a 1973 U. S. Steel industrial hygiene report, an industrial hygienist stated that hot top products at their facilities contained less than 15% of asbestos [124], while another industrial hygienist stated he was told products by “Ferro, Fesceco [assumed to be a typo of Foseco], etc.” contained between 5 and 10% asbestos [125]. Specifically, the Foseco Profax hot top product was 4% amosite and 1% chrysotile asbestos by weight [123, p. 45].

Asbestos-containing hot top liners and boards used by U. S. Steel and the steelmaking industry were custom manufactured, non-friable, pre-formed shapes that did not need to be cut, sawed, ground, or otherwise manipulated in order to be used (personal communications with Dr. Fred Toca). Thus, claims that these liners and boards were routinely cut and/or sanded are generally inaccurate. No preparation other than removing the products from the shipping pallet and securing them to the ingot mold via spring clips, wedges, metal clips, pneumatic nail guns, or a specialty apparatus were needed to secure the hot top Foseco boards within the ingot molds [123, p. 39–40].

These boards and liners would be consumed by the heat of hot tops during use [123, p. 20–21]. In a conservative thermal model of ingot teeming, temperatures were generally sufficient to degrade asbestos into harmless silicates [52, 126].

After the steel is cooled, the ingots would be stripped out of the ingot molds [120]. In the 1970s, the ingot molds could be either cleaned by vacuuming or compressed air. In 1973, U. S. Steel industrial hygienists reported that it should be mandatory that mold cleaning vacuums be equipped with proper capture bags [12]. Although the fibers were likely not asbestos (due to the high temperatures present during ingot teeming), the only available U. S. Steel industrial hygiene reports involving hot tops or molds that recorded airborne fiber concentrations that exceeded the contemporaneous PEL for asbestos was when a capture bag was not equipped on a mold cleaning vacuum [12]. At U. S. Steel facilities, the removal of asbestos debris was recommended to be performed by vacuuming rather than air compressed blowing or dry sweeping as of 1973 [12]. In 1973, moldmen responsible for cleaning ingot molds by blowing or dry sweeping would have worn respirators that were provided by U. S. Steel [12, 124, 127].

The use of hot tops and the need to clean molds were phased out of U. S. Steel manufacturing processes when continuous casting replaced ingot teeming. Continuous casting was introduced in the 1950s and grew in popularity to become the most common method for steelmaking in the 1980s [128]. Many potential hazards were eliminated, including asbestos, because this method made ingot teeming, soaking pits, and roughing mills no longer necessary [2, section 5.4].

## Materials and methods

### U. S. Steel air sampling and data collection

We examined nearly 2000 pages of historical industrial hygiene documents from 1972 through 2006. These documents detailed the use and procedures involving ACMs, as well as methods and results of asbestos sampling collected at U. S. Steel facilities. The industrial hygiene reports were obtained from various sources including prior litigation from entities other than U. S. Steel. After Paustenbach and Associates was retained on behalf of U. S. Steel in asbestos litigation, original industrial hygiene reports were provided by outside counsel for the company. Sampling for airborne concentrations of asbestos was conducted by U. S. Steel industrial hygienists according to the NIOSH 7400 guidelines. Sampling pumps typically were operated at a flow rate between 2.0 L/min to 2.8 L/min.

Air samples were typically analyzed by PCM by an internal laboratory at U. S. Steel or an accredited outside laboratory. Beginning in 1997, some samples at the Gary Works facility were analyzed by TEM performed by outside laboratories.

The U. S. Steel industrial hygiene documents were examined for exposure data and additional details regarding the jobs and tasks sampled. In addition to reviewing these documents, information regarding the steelmaking processes, job and task descriptions, department classifications, and exposure assessment initiatives relevant to the study were gathered from numerous sources (e.g., textbooks and personal communications with former steel mill employees).

### Data entry

Information from the available U. S. Steel industrial hygiene reports regarding (1) the date of sampling, (2) date of the report, (3) facility, (4) department, (5) job, (6) task, (7) airborne asbestos concentrations, (8) if respirators were used, and (9) if any ACMs were identified in the report were manually entered into a proprietary database. All data entries were checked for accuracy and “double entered” by two different staffers. Any data errors were corrected prior to the quantitative analysis. Area samples were not included in this analysis.

For samples (*n* = 21) that were reported to be below the limit of detection and for which the limit of detection was reported, half the limit of detection was substituted as the sample concentration in the dataset. For samples (*n* = 43) that were reported to have a concentration of 0 f/cc and for which no limit of detection was disclosed in the reports, a value of 0.001 f/cc (half the lowest LOD in the dataset) was substituted.

If multiple samples were collected for a worker over the course of a workday, then the multiple samples were integrated into one time-weighted average for that worker over the total duration of the sampling efforts that were performed on that day. If the industrial hygienist provided an 8-hour TWA where they calculated the average airborne asbestos concentrations for an employee, this value was used as the airborne concentration in our data set.

### Representative workday and task sampling categories

Based on the duration of sampling and whether an 8-hour TWA was calculated, each personal sample was considered either a representative workday sample or a task sample.

Samples with durations of 180 minutes or greater (*n* = 247), regardless of whether an 8-hour TWA was calculated or not in the industrial hygiene report, were considered to be representative workday samples. This was because these samples most likely characterized more than one task performed by a worker as part of their routine job duties. The cutoff value of 180 minutes was used because, historically, the air sampling pump battery life was limited and were commonly turned off during the worker’s lunch break. If the worker’s job was the same in the first and second half of their shift, as was the custom, the measured concentration would be considered representative of a full 8-hour day, regardless of which half of the shift the sample was collected. This was a common industrial hygiene practice (personal communications with Dr. Fred Toca). There were 46 data points that were considered to be representative workday samples, despite them having sampling times that were below 180 minutes or where the sample time was not reported. These were included in this category because the industrial hygienist had performed an 8-hour TWA calculation for these data points. When the sample collection time could not be determined and an industrial hygienist calculated an 8-hour TWA was not provided (*n* = 44), samples were assumed to be representative workday samples.

For samples collected less than 180 minutes, where the industrial hygienist did not calculate an 8-hour TWA, they were considered to be task exposures (*n* = 158). Task samples would have been selected based on the industrial hygienist’s professional judgment that airborne concentrations of asbestos were high during a particular assignment or task that an employee was performing. Because of this, there is an almost certainty that the air concentrations measured during these tasks generally overestimated the average workday exposure. The task samples category also includes 35 samples that were collected for 15 minutes or less that did not have an available industrial hygienist calculated 8-hour TWA. These short-term exposure samples typically were performed as the industrial hygienist aimed to quantify the “worst-case” scenarios that a worker was exposed to (e.g., tasks expected to have higher airborne concentrations of fibers).

### Data grouping

To observe trends in our dataset, we grouped the 495 personal asbestos air samples by facility, department, and job category within each department. Within each of these categories, samples were further grouped by time periods that corresponded to changes in the OSHA PEL for asbestos [30]. The four different time periods were (1) 1972 to 1975, (2) 1976 to 1985, (3) 1986 to 1993, and (4) 1994 to 2006.

### Facility

Based on industrial hygiene reports, the data were collected from 16 distinct facilities, including (1) Clairton Works, (2) Eastern Steel Division, (3) Edgar Thomson Plant, (4) Fairfield Works, (5) Fairless Works, (6) Gary Works, (7) Geneva Works, (8) Homestead Works, (9) Irvin Works, (10) Johnstown Works, (11) Mon Valley Works, (12) National Duquesne Works, (13) Neville Island Plant, (14) New Haven Works, (15) Pittsburg Works, and (16) Waukegan Works.

One industrial hygiene report had no facility listed other than “The Eastern Steel Division” [129]. This was a descriptor that could have included multiple facilities. We were unable to confirm which facility this report was referring to; therefore, the Eastern Steel Division was considered a unique facility in the dataset. Similarly, the Mon Valley Works was an integrated steelmaking operation that included the Clairton Works, Edgar Thomson Works, Irvin Works, and Fairless Works facilities [130]. It historically also included the Homestead Works and National Duquesne Works facilities, before those two plants were shut down [131]. For several industrial hygiene reports, we were unable to identify which plant was sampled under the Mon Valley Works descriptor; therefore, it was considered as a unique facility in the database.

### Department

Across these facilities, nine different departments were identified, including the (1) blast furnace, (2) boiler house, (3) central maintenance, (4) coke, (5) finishing, (6) foundry, (7) masonry, (8) melt shop, and (9) research lab. Samples from the wire rope mill (four concentrations were found in the industrial hygiene reports), were not considered for this analysis, since it is not part of the steelmaking process, but rather a secondary operation.

Whenever the department in an industrial hygiene report was not specified as one of the nine departments identified, the provided job titles, tasks, facility, and other information was used to best determine the appropriate department. Each of these instances was reviewed by former employees and industrial hygienists at steel manufacturers, as well as the authors, to ensure that the departments were properly characterized.

### Job category within department

Job classifications for each sample were determined by both the original job titles from the available industrial hygiene reports and the task(s) the employee performed during sampling. After combining the job titles from these reports, there was a total of 21 different job classifications (Table A Appendix I). These were reviewed by former employees and industrial hygienists at steel manufacturers to ensure workers within each classification had similar duties and the possible exposure to ACMs were reasonably expected to be similar. Due to concerns that the potential ACMs and asbestos exposures vary across departments for employees who may have the same job title, job classifications were further grouped into categories by department and time period. Each of the original job titles from industrial hygiene reports and how they were classified are in Table A in the *Job Groupings* section in Appendix I.

### Analysis

Stata software (version 17.0) was used to determine all arithmetic means and standard deviations, geometric means and geometric standard deviations, maximum values, and 95th percentiles for the facility, department, and job category groupings for both task and representative workday samples in each time period. Standard deviations (both arithmetic and geometric) were calculated when the sample size within a group had five or more data points and 95th percentile concentrations were calculated for groups with 20 or more data points.

When “mean” and “standard deviation” are mentioned throughout the text, it refers to the arithmetic mean and standard deviation. The entirety of the data (*n* = 495), based on the visual examination of a quantile–quantile plot, appeared to be log-normally distributed. This is partially due to the presence of many non-detect samples. When broken down into smaller data groupings, not all data groups were log-normally distributed. Due to insufficient sample size, it is not always possible to determine the distribution of every data group. For every data grouping, the arithmetic mean was equal to or greater than the geometric mean. For this reason, and in order to maintain consistency across data groups, the arithmetic mean and standard deviations were discussed throughout the paper. Geometric means and geometric standard deviations were included in Appendix IV*. Geometric Mean and Geometric Standard Deviation*.

The standard deviations help identify the spread of values observed in the data groups. Additionally, the use of the 95th percentile has been historically used in order for hygienists and their management to understand the narrowness (or breadth) of workplace exposures. Any exceedances of the contemporaneous PEL for asbestos (11 out of 495 samples), while included in our analyses, were discussed separately in Appendix III*. PEL Exceeding Instances*.

## Results

### Overall trends in the data (representative workday and task samples)

The majority of the data analyzed in this study were categorized as representative workday samples (*n* = 337; 68%), while task samples only represented 32% (*n* = 158) of the data (Table 1).

**Table 1:** Statistical analysis of all personal air sampling (representative workday samples and task samples) for airborne fiber concentrations (1972–2006).

			Asbestos Fiber Concentration by PCM (fibers/cc)			
Sample Type	Time Period	*n*	Mean	SD	95th Percentile	PEL
Representative Workday	1972–1975	45	1.09	2.02	4.50	5
	1976–1985	105	0.13	0.30	1.11	2
	1986–1993	152	0.02	0.03	0.10	0.2
	1994–2006	35	0.03	0.03	0.09	0.1
Task	1972–1975	22	3.29	5.58	13.70	5
	1976–1985	98	0.48	1.93	1.12	2
	1986–1993	6	0.01	0.003	–	0.2
	1994–2006	32	0.03	0.02	0.07	0.1
**Total**		**495**				

Empty (–) values were not calculated because the number of samples were deemed too low. Standard deviations were calculated for all samples with an *n* of 5 or greater. 95th percentiles were calculated for *n* of 20 or greater.

For representative workday and task samples, the means for each time period were below the contemporaneous PEL (Table 1). In 1972 through 1975 and 1976 through 1985 (the first two time periods), task samples in these time periods had greater means and standard deviations than the representative workday samples in the same time period (Table 1). From 1986 through 1993 and 1994 through 2006 (the last two time periods), task samples and representative workday samples both averaged 0.03 f/cc or less (Table 1).

From 1972 through 1975, the mean asbestos air concentration of all representative workday samples was 1.09 f/cc with a standard deviation of 2.02 f/cc and the 95th percentile value was 4.50 f/cc (Table 1). From 1976 through 1985, the mean of representative workday samples decreased to 0.13 f/cc, standard deviation to 0.30 f/cc, and the 95th percentile value to 1.11 f/cc (Table 1). From 1986 through 1993, these representative workday samples further decreased to have a mean of 0.02 f/cc with a standard deviation of 0.03 f/cc and a 95th percentile value of 0.10 f/cc (Table 1). From 1994 through 2006, only the Gary Works facility was sampled and had a mean representative workday fiber concentration of 0.03 f/cc, standard deviation of 0.03 f/cc, and a 95^th^ percentile value of 0.09 f/cc (Table 1).

From 1972 through 1975, the mean asbestos air concentration of all task samples was 3.29 f/cc with a standard deviation of 5.58 f/cc and the 95th percentile value was 13.70 f/cc (Table 1). From 1976 through 1985, the mean of task samples decreased to 0.48 f/cc, standard deviation to 1.93 f/cc, and the 95th percentile value to 1.12 f/cc (Table 1). From 1986 through 1993, these task samples further decreased to have a mean of 0.01 f/cc with a standard deviation of 0.003 f/cc (Table 1). From 1994 through 2006, only the Gary Works facility was sampled and had a mean task sample fiber concentration of 0.03 f/cc, standard deviation of 0.02 f/cc, and a 95th percentile value of 0.07 f/cc (Table 1).

Task samples from 1972 through 1975 were the only group from Table 1 that had a 95th percentile or a mean added to the standard deviation that exceeded the contemporaneous PEL (Table 1).

**Fig. 1:**
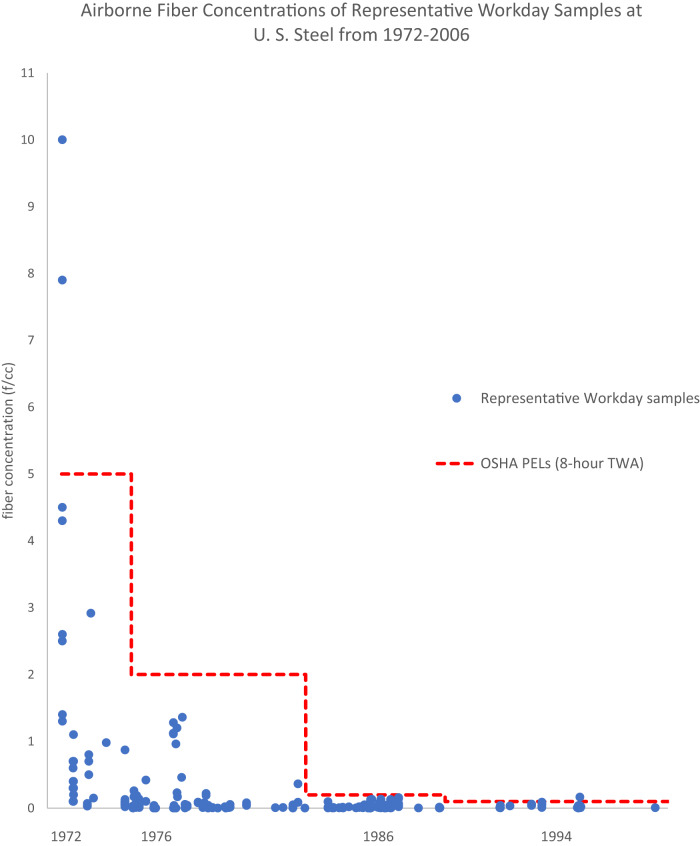
Airborne fiber concentrations of representative workday samples at U. S. Steel from 1972 to 2006. Scatterplot of airborne fiber concentrations, representative workday samples (blue circles), by their sampling dates from 1972 to 2006 in comparison to the contemporaneous OSHA PELs as an 8-h TWA (red dotted line).

**Fig. 2:**
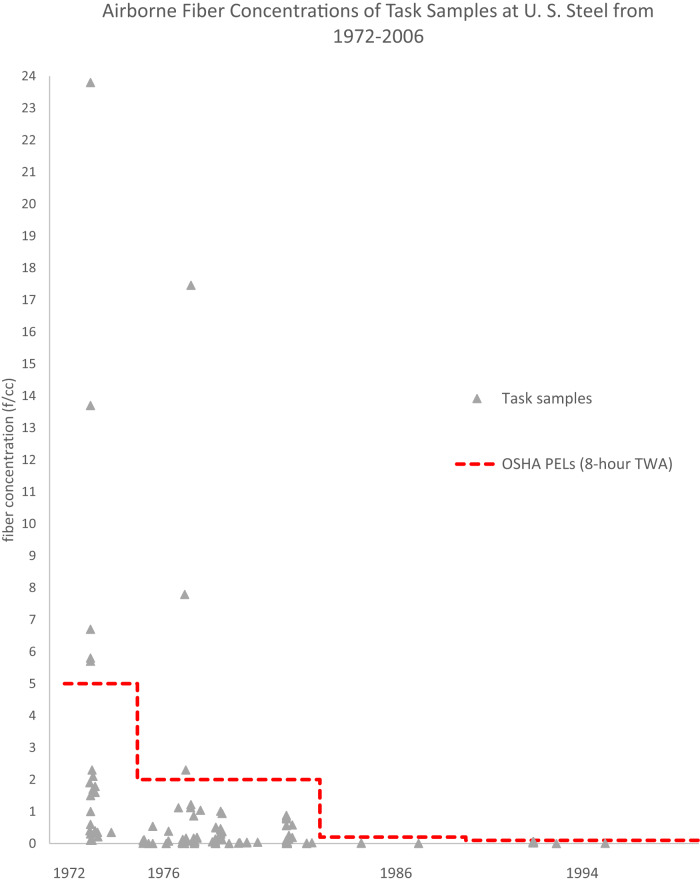
Airborne fiber concentrations of task samples at U. S. Steel from 1972 to 2006. Scatterplot of airborne fiber concentrations, task samples (gray triangles), by their sampling dates from 1972 to 2006 in comparison to the contemporaneous OSHA PELs as an 8-h TWA (red dotted line).

Figure 1 shows the temporal distribution of every representative workday sample in the dataset from 1972 to 2006. Figure 2 shows the temporal distribution of every task sample in the dataset from 1972 to 2006. In general, a decrease in airborne asbestos concentrations over time is observed. No samples greater than 2 f/cc are observed after 1978 and no samples greater than 0.2 f/cc are observed after 1984 (Fig. 1 and 2).

Across the entire dataset, 11 out of the 495 total samples (2.2%) exceeded the contemporaneous OSHA PEL (8-hour TWA) for asbestos, with eight of these samples being task samples that are likely not representative of 8-hour TWAs. The remaining three samples were representative workday samples (two of which had unknown sampling times). Details regarding these 11 samples are discussed in Appendix III*. PEL Exceeding Instances*.

Specifically for representative workday samples, just 0.9% of samples from 1972 to 2006 exceeded the contemporaneous OSHA PEL. Only one of the PEL exceeding representative workday samples had a sampling time that exceeded 180 minutes. The two other PEL exceeding representative workday samples had unknown sampling times. It was unclear whether the TWAs for these reports were 8-hour calculations, however these were assumed to be representative workday samples per the methods described previously.

When compared to the current occupational standards, 16.6% of representative workday samples from 1972 through 2006 exceeded the current PEL of 0.1 f/cc. For representative workday samples past the year 1980, only nine out of a possible 213 samples (4.2%) exceeded 0.1 f/cc.

### Overview of the summary statistics by facility

None of the mean fiber concentrations, both representative workday and task samples, when broken down by the 16 facilities across each time period exceeded any of the contemporaneous OSHA PELs for asbestos (Tables 2 and 3).

**Table 2:** Statistical analysis of all task samples for airborne fiber concentrations at 16 different U. S. Steel Facilities (1972–2006).

			Fiber Concentration by PCM (fibers/cc)				
Facility Name	Time Period	*n*	Mean	SD	95th Percentile	Max	PEL
Edgar-Thomson Plant	1972–1975	1	0.35	–	–	0.35	5
	1976–1985	7	0.06	0.10	–	0.25	2
Fairfield Works	1976–1985	16	0.21	0.26	–	1.01	2
Fairless Works	1976–1985	35	1.01	3.17	7.79	17.46	2
Gary Works	1972–1975	16	4.23	6.32	–	23.80	5
	1976–1985	4	0.45	–	–	0.94	2
	1986–1993	6	0.01	0.004	–	0.01	0.2
	1994–2006	32	0.03	0.02	0.07	0.07	0.1
Geneva Works	1976–1985	4	0.15	–	–	0.38	2
Homestead Works	1972–1975	5	0.87	0.76	–	1.79	5
	1976–1985	10	0.04	0.04	–	0.12	2
Irvin Works	1976–1985	1	0.001	–	–	0.001	2
Mon Valley Works	1976–1985	2	0.39	–	–	0.58	5
National Duquesne Works	1976–1985	2	0.04	–	–	0.04	2
Neville Island Plant	1976–1985	16	0.28	0.33	–	0.88	2
Pittsburg Works	1976–1985	1	0.05	–	–	0.05	2
**Total**		**158**					

Empty (–) values were not calculated because the number of samples were deemed too low. Standard deviations were calculated for all samples with an *n* of 5 or greater. 95th percentiles were calculated for *n* of 20 or greater.

**Table 3:** Statistical analysis of all representative workday samples for airborne fiber concentrations at 16 different U. S. Steel Facilities (1972–2006).

			Fiber Concentration by PCM (fibers/cc)				
Facility Name	Time Period	*n*	Mean	SD	95th Percentile	Max	PEL
Clairton Works	1976–1985	8	0.06	0.03	–	0.09	2
	1986–1993	48	0.03	0.04	0.13	0.16	0.2
Eastern Steel Division	1976–1985	2	0.21	–	–	0.22	2
Edgar-Thomson Plant	1972–1975	1	0.98	–	–	0.98	5
	1976–1985	10	0.01	0.02	–	0.05	2
	1986–1993	58	0.02	0.03	0.13	0.13	0.2
Fairfield Works	1976–1985	6	0.16	0.12	–	0.36	2
	1986–1993	7	0.01	0.01	–	0.04	0.2
Fairless Works	1972–1975	8	4.31	3.14	–	10.00	5
	1976–1985	27	0.37	0.51	1.28	1.36	2
	1986–1993	15	0.01	0.01	–	0.03	0.2
Gary Works	1972–1975	4	0.68	–	–	0.80	5
	1986–1993	16	0.001	0.01	–	0.02	0.2
	1994–2006	35	0.03	0.03	0.09	0.17	0.1
Homestead Works	1972–1975	6	0.54	1.16	–	2.92	5
	1976–1985	27	0.04	0.06	0.17	0.26	2
Johnstown Works	1972–1975	11	0.14	0.24	–	0.87	5
Mon Valley Works	1976–1985	2	0.01	–	–	0.01	5
	1986–1993	8	0.01	0.002	–	0.01	2
National Duquesne Works	1976–1985	9	0.03	0.02	–	0.07	2
Neville Island Plant	1972–1975	15	0.41	0.29	–	1.10	5
	1976–1985	2	0.01	0.01	–	0.02	2
New Haven Works	1976–1985	2	0.001	–	–	0.001	2
Pittsburg Works	1976–1985	2	0.01	–	–	0.01	2
Waukegan Works	1976–1985	8	0.03	0.01	–	0.04	2
**Total**		**337**					

Empty (–) values were not calculated because the number of samples were deemed too low. Standard deviations were calculated for all samples with an *n* of 5 or greater. 95th percentiles were calculated for *n* of 20 or greater.

Of the task samples, the only facilities across any time period that had reported concentrations greater than the contemporaneous PEL were Gary Works from 1972 to 1975 (max value = 23.80 f/cc) and Fairless Works from 1976 to 1985 (max value = 17.46 f/cc) (Table 2). Gary Works from 1972 to 1975 (mean = 4.23 f/cc; SD = 6.32 f/cc) and Fairless Works from 1976 to 1985 (mean = 1.01 f/cc; SD = 3.17 f/cc; 95th percentile = 7.79 f/cc) were also the only groups where the mean of task samples added to the standard deviation and/or the 95th percentile value exceeded the contemporaneous PEL (Table 2). Details regarding the eight PEL exceeding task samples from the entire dataset are discussed in Appendix III*. PEL Exceeding Instances*.

Of the representative workday samples, the only facilities across any time period that had a reported concentration greater than the contemporaneous PEL were Fairless Works from 1972 to 1975 (max value = 10 f/cc) and Gary Works from 1994 to 2006 (max value = 0.17 f/cc) (Table 3). The mean added to the standard deviation and 95th percentile for Gary Works from 1994 to 2006 (mean = 0.03 f/cc; SD = 0.03 f/cc; 95th percentile = 0.09 f/cc) was below the contemporaneous PEL of 0.1 f/cc (Table 3). Fairless Works from 1972 to 1975 is the only group of representative workday samples across any time period with a mean added to the standard deviation (mean = 4.31 f/cc; SD = 3.14 f/cc) that exceeded the contemporaneous PEL (5 f/cc) (Table 3). Details regarding the three PEL exceeding representative workday samples from the entire dataset are discussed in Appendix III*. PEL Exceeding Instances*.

### Overview of the summary statistics by department

None of the mean fiber concentrations, representative workday, or task samples, when broken down by the nine departments across each time period, exceeded any of the contemporaneous PELs for asbestos (Tables 4 and 5).

**Table 4:** Statistical analysis of all task samples for airborne fiber concentrations at nine different U. S. Steel Departments (1972–2006).

			Fiber Concentration by PCM (fibers/cc)				
Department	Time Period	*n*	Mean	SD	95th Percentile	Max	PEL
Blast Furnace	1972–1975	3	1.26	–	–	1.79	5
Boiler House	1976–1985	5	0.02	0.01	–	0.04	2
	1986–1993	2	0.005	–	–	0.01	0.2
	1994–2006	30	0.03	0.02	0.07	0.07	0.1
Central Maintenance	1972–1975	1	0.35	–	–	0.35	5
	1976–1985	47	0.77	2.74	1.22	17.46	2
	1986–1993	4	0.01	0.003	–	0.01	0.2
	1994–2006	1	0.03	–	–	0.03	0.1
Coke	1976–1985	24	0.26	0.49	1.01	2.30	2
Finishing	1972–1975	1	0.21	–	–	0.21	5
	1976–1985	8	0.23	0.31	–	0.86	2
Masonry	1972–1975	1	0.35	–	–	0.35	5
	1976–1985	5	0.06	0.04	–	0.12	2
Melt Shop	1972–1975	16	4.23	6.32	–	23.80	5
	1976–1985	7	0.19	0.38	–	1.04	2
	1994–2006	1	0.001	–	–	0.001	0.1
Research Labs	1976–1985	2	0.50	–	–	0.51	2
**Total**		**158**					

Empty (–) values were not calculated because the number of samples were deemed too low. Standard deviations were calculated for all samples with an *n* of 5 or greater. 95th percentiles were calculated for *n* of 20 or greater.

**Table 5:** Statistical analysis of all representative workday samples for airborne fiber concentrations at nine different U. S. Steel Departments (1972–2006).

			Fiber Concentration by PCM (fibers/cc)				
Department	Time Period	*n*	Mean	SD	95th Percentile	Max	PEL
Blast Furnace	1972–1975	1	2.92	–	–	2.92	5
	1976–1985	6	0.12	0.08	–	0.23	2
Boiler House	1976–1985	12	0.06	0.10	–	0.36	2
	1986–1993	37	0.01	0.02	0.08	0.10	0.2
	1994–2006	31	0.02	0.03	0.09	0.17	0.1
Central Maintenance	1972–1975	15	0.41	0.29	–	1.10	5
	1976–1985	6	0.05	0.04	–	0.09	2
	1986–1993	10	0.01	0.01	–	0.03	0.2
	1994–2006	1	0.01	–	–	0.01	0.1
Coke	1976–1985	6	0.07	0.05	–	0.17	2
	1986–1993	46	0.03	0.04	0.13	0.16	0.2
Finishing	1972–1975	2	0.51	–	–	0.87	5
	1976–1985	51	0.16	0.35	1.20	1.28	2
	1986–1993	42	0.02	0.04	0.13	0.13	0.2
Foundry	1972–1975	10	0.07	0.03	–	0.13	5
Masonry	1972–1975	5	0.23	0.42	–	0.98	5
	1976–1985	6	0.07	0.11	–	0.22	2
Melt Shop	1972–1975	12	3.10	3.08	–	10.00	5
	1976–1985	12	0.25	0.45	–	1.36	2
	1986–1993	5	0.01	0.01	–	0.02	0.2
	1994–2006	3	0.05	–	–	0.06	0.1
Research Labs	1976–1985	6	0.004	0.01	–	0.02	2
	1986–1993	12	0.01	0.004	–	0.01	0.2
**Total**		**337**					

Empty (–) values were not calculated because the number of samples were deemed too low. Standard deviations were calculated for all samples with an *n* of 5 or greater. 95th percentiles were calculated for *n* of 20 or greater.

Of the task samples, the only departments across any time period that had reported concentrations greater than the contemporaneous PEL were the melt shop from 1972 to 1975 (max value = 23.80 f/cc), the central maintenance department from 1976 to 1985 (max value = 17.46 f/cc), and the coke department from 1976 to 1985 (max value = 2.30 f/cc) (Table 4). The melt shop from 1972 to 1975 (mean = 4.23 f/cc; SD = 6.32 f/cc) and central maintenance from 1976 to 1985 (mean = 0.77 f/cc; SD = 2.74 f/cc) were the only department groups where the mean of task samples added to the standard deviation exceeded the contemporaneous PEL (Table 4). None of the calculated 95th percentiles of any departments grouped by time period exceeded contemporaneous PEL. Details regarding the eight PEL exceeding task samples from the entire dataset are discussed in Appendix III*. PEL Exceeding Instances*.

Of the representative workday samples, the only departments across any time period that had reported concentrations greater than the contemporaneous PEL were the melt shop department from 1972 to 1975 (max value = 10 f/cc) and the boiler house department from 1994 to 2006 (max value = 0.17 f/cc) (Table 5). The melt shop from 1972 to 1975 (mean = 3.10 f/cc; SD = 3.08 f/cc) was the only department where the mean of representative workday samples added to the standard deviation exceeded the contemporaneous PEL (Table 5). None of the calculated 95th percentiles of any departments grouped by time period exceeded contemporaneous PEL. Details regarding the three PEL exceeding representative workday samples from the entire dataset are discussed in Appendix III*. PEL Exceeding Instances*.

### Summary statistics by department job category

Tables 6–22 examined the job categories within each department across time periods. As discussed previously in the methods, job categories were considered for each department distinctly due to concerns that asbestos exposures may vary between people with the same title in different departments.

**Table 6:** Task sample airborne fiber concentrations for Blast Furnace (BF) operators in that department (1972–1975).

			Fiber Concentration by PCM (fibers/cc)				
Job Title	Time Period	*n*	Mean	SD	95th percentile	Max	PEL
BF Operator	1972–1975	3	1.26	–	–	1.79	5

Empty (–) values were not calculated because the number of samples were deemed too low. Standard deviations were calculated for all samples with an *n* of 5 or greater. 95th percentiles were calculated for *n* of 20 or greater.

**Table 7:** Representative workday sample airborne fiber  concentrations for Blast Furnace (BF) operators in that department (1972–1985).

			Fiber Concentration by PCM (fibers/cc)				
Job Title	Time Period	*n*	Mean	SD	95th percentile	Max	PEL
BF Operator	**Overall**	7	0.52	1.06	–	2.92	
	1972–1975	1	2.92	–	–	2.92	5
	1976–1985	6	0.12	0.08	–	0.23	2

Empty (–) values were not calculated because the number of samples were deemed too low. Standard deviations were calculated for all samples with an *n* of 5 or greater. 95th percentiles were calculated for *n* of 20 or greater.

**Table 8:** Task sample airborne fiber concentrations in the Boiler House department grouped by job category (1972–2006).

			Fiber Concentration by PCM (fibers/cc)				
Job Title	Time Period	*n*	Mean	SD	95th Percentile	Max	PEL
BH Operator	1994–2006	8	0.03	0.02	–	0.06	0.1
Mechanical Maintenance	**Overall**	24	0.03	0.02	0.07	0.07	
	1976–1985	2	0.01	–	–	0.01	2
	1986–1993	2	0.005	–	–	0.01	0.2
	1994–2006	20	0.04	0.01	0.07	0.07	0.1
Boiler Cleaner	1976–1985	3	0.03	–	–	0.04	2
Insulator	1994–2006	2	0.03	–	–	0.04	0.1

Empty (–) values were not calculated because the number of samples were deemed too low. Standard deviations were calculated for all samples with an *n* of 5 or greater. 95th percentiles were calculated for *n* of 20 or greater.

**Table 9:** Representative workday sample airborne fiber concentrations in the Boiler House department grouped by job category (1972–2006).

			Fiber Concentration by PCM (fibers/cc)				
Job Title	Time Period	*n*	Mean	SD	95th Percentile	Max	PEL
BH Operator	Overall	43	0.01	0.02	0.05	0.06	
	1976–1985	6	0.02	0.02	–	0.06	2
	1986–1993	22	0.01	0.01	0.02	0.03	0.2
	1994–2006	15	0.02	0.02	–	0.06	0.1
Craftsman	1994–2006	6	0.02	0.01	–		0.1
Mechanical Maintenance	Overall	22	0.03	0.04	0.10	0.17	
	1976–1985	1	0.001	–	–	0.001	2
	1986–1993	12	0.02	0.02	–	0.10	0.2
	1994–2006	9	0.04	0.05	–	0.17	0.1
Boiler Cleaner	1976–1985	3	0.18	–	–	0.36	2
Oversight	Overall	6	0.01	0.01	–	0.02	
	1976–1985	2	0.01	–	–	0.01	2
	1986–1993	3	0.01	–	–	0.02	0.2
	1994–2006	1	0.01	–	–	0.01	0.1

Empty (–) values were not calculated because the number of samples were deemed too low. Standard deviations were calculated for all samples with an *n* of 5 or greater. 95th percentiles were calculated for *n* of 20 or greater.

**Table 10:** Task sample airborne fiber concentrations in the Central Maintenance department grouped by job category (1972–2006).

			Fiber Concentration by PCM (fibers/cc)				
Job Title	Time Period	*n*	Mean	SD	95th percentile	Max	PEL
Craftsman	Overall	10	0.19	0.17	–	0.58	
	1972–1975	1	0.35	–	–	0.35	5
	1976–1985	9	0.17	0.17	–	0.58	2
Mechanical Maintenance	Overall	23	0.21	0.34	0.88	0.94	
	1976–1985	19	0.26	0.36	–	0.94	2
	1986–1993	4	0.01	–	–	0.01	0.2
Brake Repairman	1976–1985	2	0.05	–	–	0.09	5
Electrician	1976–1985	7	0.09	0.13	–	0.38	2
Insulator	1976–1985	2	12.63	–	–	17.46	2
Motor Inspector	Overall	6	0.64	0.56	–	1.22	
	1976–1985	5	0.76	0.53	–	1.22	2
	1994–2006	1	0.03	–	–	0.03	0.1
Oversight	1976–1985	3	0.02	–	–	0.04	2

Empty (–) values were not calculated because the number of samples were deemed too low. Standard deviations were calculated for all samples with an *n* of 5 or greater. 95th percentiles were calculated for *n* of 20 or greater.

**Table 11:** Representative workday sample airborne fiber concentrations in the Central Maintenance department grouped by job category (1972–2006).

			Fiber Concentration by PCM (fibers/cc)				
Job Title	Time Period	*n*	Mean	SD	95th percentile	Max	PEL
Mechanical Maintenance	Overall	24	0.26	0.30	0.70	1.10	
	1972–1975	15	0.41	0.29	–	1.10	5
	1986–1993	9	0.01	0.01	–	0.03	0.2
Brake Repairman	Overall	4	0.01	–	–	0.02	
	1976–1985	2	0.02	–	–	0.02	5
	1986–1993	1	0.02	–	–	0.02	0.2
	1994–2006	1	0.01	–	–	0.01	0.1
Insulator	1976–1985	3	0.08	–	–	0.09	2
Motor Inspector	1976–1985	1	0.01	–	–	0.01	2

Empty (–) values were not calculated because the number of samples were deemed too low. Standard deviations were calculated for all samples with an *n* of 5 or greater. 95th percentiles were calculated for *n* of 20 or greater.

**Table 12:** Task sample airborne fiber concentrations in the Coke department grouped by job category (1976–1985).

			Fiber Concentration by PCM (fibers/cc)				
Job Title	Time Period	*n*	Mean	SD	95th percentile	Max	PEL
Craftsman	1976–1985	23	0.27	0.50	1.01	2.30	2
CO Operator	1976–1985	1	0.001	–	–	0.001	2

Empty (–) values were not calculated because the number of samples were deemed too low. Standard deviations were calculated for all samples with an *n* of 5 or greater. 95th percentiles were calculated for *n* of 20 or greater.

**Table 13:** Representative workday sample airborne fiber concentrations in the Coke department grouped by job category (1976–1993).

			Fiber Concentration by PCM (fibers/cc)				
Job Title	Time Period	*n*	Mean	SD	95th percentile	Max	PEL
Craftsman	Overall	28	0.04	0.04	0.13	0.17	
	1976–1985	6	0.07	0.05	–	0.17	2
	1986–1993	22	0.04	0.04	0.13	0.13	0.2
CO Operator	1986–1993	1	0.16	–	–	0.16	0.2
Insulator	1986–1993	2	0.04	–	–	0.05	0.2
Mechanical Maintenance	1986–1993	19	0.01	0.01	–	0.05	0.2
Oversight	1986–1993	2	0.08	–	–	0.14	0.2

Empty (–) values were not calculated because the number of samples were deemed too low. Standard deviations were calculated for all samples with an *n* of 5 or greater. 95th percentiles were calculated for *n* of 20 or greater.

**Table 14:** Task sample airborne fiber concentrations in the Finishing department grouped by job category (1972–1985).

			Fiber Concentration by PCM (fibers/cc)				
Job Title	Time Period	*n*	Mean	SD	95th percentile	Max	PEL
Finishing Operator	**Overall**	5	0.18	0.22	–	0.54	
	1972–1975	1	0.21	–	–	0.21	5
	1976–1985	4	0.18	–	–	0.54	2
Craftsman	1976–1985	1	0.05	–	–	0.05	2
Motor Inspector	1976–1985	3	0.37	–	–	0.86	2

Empty (–) values were not calculated because the number of samples were deemed too low. Standard deviations were calculated for all samples with an *n* of 5 or greater. 95th percentiles were calculated for *n* of 20 or greater.

**Table 15:** Representative workday sample airborne fiber concentrations in the Finishing department grouped by job category (1972–1993).

			Fiber Concentration by PCM (fibers/cc)				
Job Title	Time Period	*n*	Mean	SD	95th percentile	Max	PEL
Finishing Operator	Overall	34	0.13	0.30	1.11	1.20	
	1972–1975	2	0.51	–	–	0.87	5
	1976–1985	24	0.14	0.32	1.11	1.20	2
	1986–1993	8	0.01	0.01	–	0.02	0.2
Craftsman	Overall	4	0.01	–	–	0.01	
	1976–1985	1	0.01	–	–	0.01	2
	1986–1993	3	0.01	–	–	0.01	0.2
Crane Operator	Overall	23	0.06	0.23	0.13	1.12	
	1976–1985	9	0.13	0.37	–	1.12	2
	1986–1993	14	0.02	0.04	–	0.13	0.2
Mechanical Maintenance	Overall	7	0.45	0.59	–	1.28	
	1976–1985	5	0.62	0.62	–	1.28	2
	1986–1993	2	0.02	–	–	0.04	0.2
Motor Inspector	1976–1985	1	0.01	–	–	0.01	2
Oversight	1976–1985	4	0.03	–	–	0.04	2
Process Support	Overall	22	0.03	0.04	0.13	0.13	
	1976–1985	7	0.02	0.02	–	0.05	2
	1986–1993	15	0.04	0.05	–	0.13	0.2

Empty (–) values were not calculated because the number of samples were deemed too low. Standard deviations were calculated for all samples with an *n* of 5 or greater. 95th percentiles were calculated for *n* of 20 or greater.

**Table 16:** Representative workday sample airborne fiber concentrations in the Foundry department grouped by job category (1972–1975).

			Fiber Concentration by PCM (fibers/cc)				
Job Title	Time Period	*n*	Mean	SD	95th Percentile	Max	PEL
Foundry Operator	1972–1975	1	0.13	–	–	0.13	5
Foundryman	1972–1975	9	0.06	0.03	–	0.10	5

Empty (–) values were not calculated because the number of samples were deemed too low. Standard deviations were calculated for all samples with an *n* of 5 or greater. 95th percentiles were calculated for *n* of 20 or greater.

**Table 17:** Task sample airborne fiber concentrations in the Masonry department grouped by job category (1972–1985).

				Fiber Concentration by PCM (fibers/cc)			
Job Title	Time Period	*n*	Mean	SD	95th percentile	Max	PEL
Bricklayer	Overall	6	0.11	0.12	–	0.35	
	1972–1975	1	0.35	–	–	0.35	5
	1976–1985	5	0.06	0.04	–	0.12	2

Empty (–) values were not calculated because the number of samples were deemed too low. Standard deviations were calculated for all samples with an *n* of 5 or greater. 95th percentiles were calculated for *n* of 20 or greater.

**Table 18:** Representative workday sample airborne fiber concentrations in the Masonry department grouped by job category (1972–1985).

			Fiber Concentration by PCM (fibers/cc)				
Job Title	Time Period	*n*	Mean	SD	95th percentile	Max	PEL
Bricklayer	Overall	11	0.14	0.29	–	0.98	
	1972–1975	5	0.23	0.42	–	0.98	5
	1976–1985	6	0.07	0.11	–	0.22	2

Empty (–) values were not calculated because the number of samples were deemed too low. Standard deviations were calculated for all samples with an *n* of 5 or greater. 95th percentiles were calculated for *n* of 20 or greater.

**Table 19:** Task sample airborne fiber concentrations in the Melt Shop department grouped by job category (1972–2006).

			Fiber Concentration by PCM (fibers/cc)				
Job Title	Time Period	*n*	Mean	SD	95th percentile	Max	PEL
Crane/Forklift Operator	1972–1975	5	0.76	0.94	–	2.30	5
Mechanical Maintenance	1976–1985	1	0.001	–	–	0.001	2
Moldman	1972–1975	11	5.81	7.12	–	23.80	5
Motor Inspector	1976–1985	2	0.62	–	–	1.04	2
Melt Shop Operator	1976–1985	3	0.02	–	–	0.03	2
Oversight	Overall	2	0.001	–	–	0.001	
	1976–1985	1	0.001	–	–	0.001	2
	1994–2006	1	0.001	–	–	0.001	0.1

Empty (–) values were not calculated because the number of samples were deemed too low. Standard deviations were calculated for all samples with an *n* of 5 or greater. 95th percentiles were calculated for *n* of 20 or greater.

**Table 20:** Representative workday sample airborne fiber concentrations in the Melt Shop department grouped by job category (1972–2006).

			Fiber Concentration by PCM (fibers/cc)				
Job Title	Time Period	*n*	Mean	SD	95th percentile	Max	PEL
Crane/Forklift Operator	1986–1993	3	0.003	–	–	0.006	0.2
Mechanical Maintenance	Overall	5	0.04	0.03	–	0.08	
	1976–1985	3	0.06	–	–	0.08	2
	1986–1993	2	0.01	–	–	0.02	0.2
Moldman	Overall	12	3.06	3.12	–	10.00	
	1972–1975	11	3.34	3.11	–	10.00	5
	1976–1985	1	0.01	–	–	0.01	0.2
Motor Inspector	1976–1985	2	0.91	–	–	1.36	2
Melt Shop Operator	1976–1985	2	0.49	–	–	0.96	2
Oversight	Overall	7	0.02	0.02	–	0.06	
	1976–1985	4	0.002	–	–	0.004	2
	1994–2006	3	0.05	–	–	0.06	0.1

Empty (–) values were not calculated because the number of samples were deemed too low. Standard deviations were calculated for all samples with an *n* of 5 or greater. 95th percentiles were calculated for *n* of 20 or greater.

**Table 21:** Task sample airborne fiber concentrations in the Research Labs department grouped by job category (1972–1985).

			Fiber Concentration by PCM (fibers/cc)				
Job Title	Time Period	*n*	Mean	SD	95th percentile	Max	PEL
Research Tester	1976–1985	2	0.50	–	–	0.51	2

Empty (–) values were not calculated because the number of samples were deemed too low. Standard deviations were calculated for all samples with an *n* of 5 or greater. 95th percentiles were calculated for *n* of 20 or greater.

**Table 22:** Representative workday sample airborne fiber concentrations in the Research Labs department grouped by job category (1976–1993).

			Fiber Concentration by PCM (fibers/cc)				
Job Title	Time Period	*n*	Mean	SD	95th percentile	Max	PEL
Research Tester	Overall	14	0.00	0.005	–	0.02	
	1976–1985	6	0.004	0.01	–	0.02	2
	1986–1993	8	0.005	0.002	–	0.01	0.2
Janitor	1986–1993	4	0.005	–	–	0.01	0.2

Empty (–) values were not calculated because the number of samples were deemed too low. Standard deviations were calculated for all samples with an *n* of 5 or greater. 95th percentiles were calculated for *n* of 20 or greater.

Of the task samples, only three job categories—moldmen in the melt shop from 1972 to 1975 (max value = 23.80 f/cc) (Table 19), insulators in the central maintenance department from 1976 to 1985 (max value = 17.46) (Table 10), and craftsman in the coke department from 1976 to 1985 (max value = 2.30 f/cc) (Table 12)—had maximum concentrations above the contemporaneous OSHA PEL. Details regarding the eight total PEL exceeding task samples in the dataset are discussed in Appendix III*. PEL Exceeding Instances*.

The mean and mean added to the standard deviation for task samples of craftsmen in the coke department from 1976 to 1985 (mean = 0.27 f/cc; SD = 0.50; *n* = 23; 95th = 1.01) was below the contemporaneous PEL (2 f/cc) (Table 12). Task samples of moldmen in the melt shop from 1972 to 1975 had a mean (mean = 5.81 f/cc; SD = 7.12 f/cc; *n* = 11) that exceeded the contemporaneous PEL (5 f/cc) (Table 19). Task samples of insulators in the central maintenance department from 1976 to 1985 also had a mean (mean = 12.63 f/cc; *n* = 2) that exceeded the contemporaneous PEL (2 f/cc) (Table 10). However, the representative workday samples for insulators in the central maintenance department from 1976 to 1985 (*n* = 3) had a mean value of 0.08 f/cc and a maximum concentration of 0.09 f/cc (Table 11).

Of the representative workday samples, moldmen in the meltshop from 1972 to 1975 (*n* = 11) was the only job category in which the mean (3.34 f/cc) added to the standard deviation (3.11 f/cc) exceeded contemporaneous PEL (5 f/cc) (Table 20). This job category had a maximum concentration of 10 f/cc reported. Details regarding the three PEL exceeding representative workday samples in the dataset are discussed in Appendix III*. PEL Exceeding Instances*.

### Airborne asbestos concentrations (1972–1975)

For samples from all facilities and departments in this time period (*n* = 67), the mean of representative workday samples (*n* = 45) was 1.09 f/cc, the standard deviation was 2.02 f/cc, and the 95th percentile was 4.50 f/cc (Table 1). The mean of task samples (*n* = 22) sampled during this time was 3.29 f/cc, the standard deviation was 5.58 f/cc, and 95th percentile was 13.70 f/cc (Table 1). There was a total of 7 datapoints (2 representative workday samples and 5 task samples) from 1972 to 1975 that were above the contemporaneous PEL of 5 f/cc. These all occurred in Moldmen that were cleaning ingot molds and are described in detail in Appendix III*. PEL Exceeding Instances*.

### 1972–1975 results by facility

**Fig. 3:**
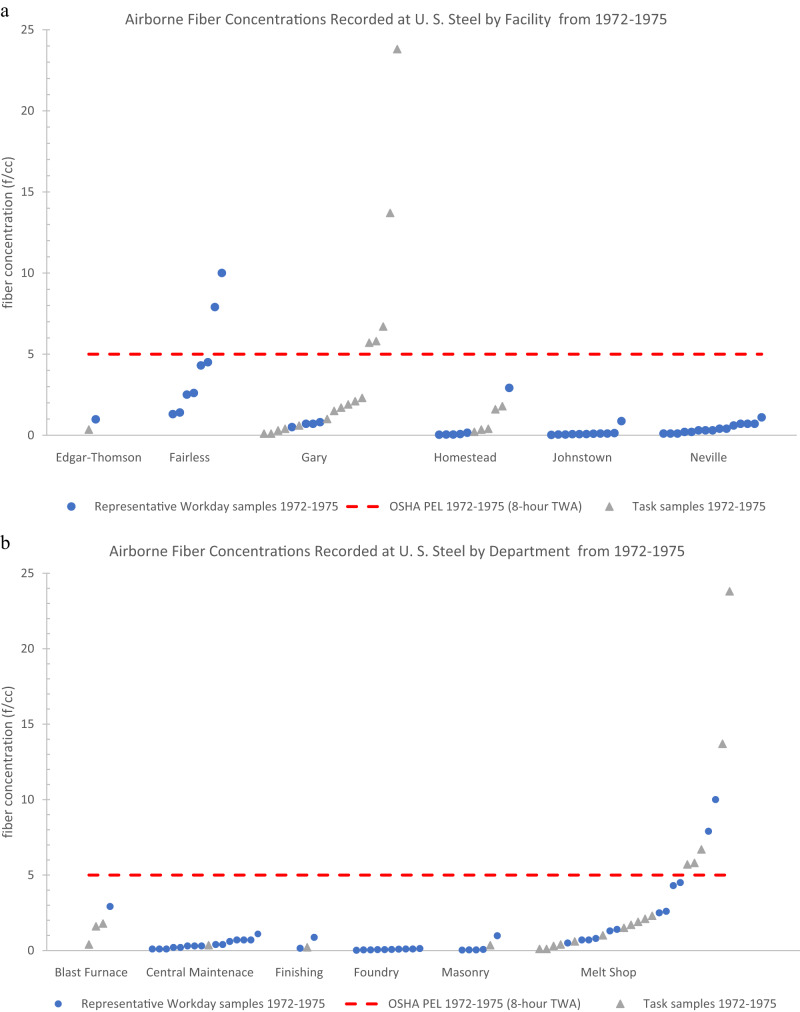
Airborne fiber concentrations recorded at U. S. Steel from 1972 to 1975. **a** Scatterplot of individual data points (representative workday samples are blue circles; task samples are gray triangles) from 1972 to 1975 across each facility for which asbestos air samples were recorded. Seven datapoints, two at Fairless Works and five at Gary Works, out of 67 total samples for this time period exceeded the contemporaneous PEL (red dotted line) of 5 f/cc. **b**. Scatterplot of individual data points (representative workday samples are blue circles; task samples are gray triangles) from 1972 to 1975 across each department for which asbestos air samples were recorded. Seven datapoints from the melt shop department out of the total of 67 samples during this time period exceeded the contemporaneous PEL (red dotted line) of 5 f/cc.

Figure 3a presents airborne asbestos concentrations based on personal samples collected from 1972 until 1975 by facility. Data were collected at six facilities during this time: (1) Edgar Thomson Plant, (2) Fairless Works, (3) Gary Works, (4) Homestead Works, (5) Johnstown Works, and (6) Neville Island Plant. Out of these six facilities, Fairless Works representative workday samples and Gary Works task samples were the only two in this time period where the mean airborne asbestos concentration added to the standard deviation or 95th percentile exceeded the contemporaneous PEL (Tables 2 and 3).

For Fairless Works, there were eight airborne asbestos personal samples that were classified as representative workday exposures collected between 1972 and 1975. The mean of these samples was 4.31 f/cc and the standard deviation was 3.14 f/cc (Table 3). As mentioned previously, this was the only representative workday sample group in which the mean added to the associated standard deviation exceeded the contemporaneous PEL (5 f/cc). This was because of two PEL exceeding samples that occurred in the melt shop department. Both samples were for the same moldman employee who was not following proper procedures and one exceeded the OSHA 15-minute ceiling limit of 10 f/cc [12].

For Gary Works, there were 20 airborne asbestos personal samples (16 task and 4 representative workday samples) collected between 1972 and 1975. The representative workday samples had a mean concentration of 0.68 f/cc and a maximum concentration of 0.80 f/cc was reported, both well below the contemporaneous PEL of 5 f/cc (Table 3). The task samples for this category, as mentioned previously, had a mean added to the standard deviation value (mean = 4.23 f/cc; SD = 6.32 f/cc) that exceeded the contemporaneous PEL (5 f/cc) (Table 2). There were five PEL exceeding task samples at Gary Works during this time period, two of which also exceeded the OSHA 15-minute ceiling limit of 10 f/cc. Similar to the two representative workday tasks at Fairless Works, these samples also involved moldmen from the melt shop department [124].

Excluding Gary Works and Fairless Works samples from 1972 through 1975, there were four facilities where samples were collected during this time. For samples in these four facilities (*n* = 39), the mean of the representative workday samples (*n* = 33) was 0.36 f/cc, the standard deviation was 0.55 f/cc, and the 95th percentile was 1.10 f/cc. Other than Gary Works, only Edgar-Thomson and Homestead Works had task samples. The mean of task samples (*n* = 6) in these facilities during this time was 0.78 /cc, the standard deviation was 0.71 f/cc, and the 95th percentile was 1.79 f/cc.

### 1972–1975 results by department

Figure 3b presents airborne asbestos concentrations based on personal samples collected from 1972 until 1975 by department. Data were collected at six departments during this time period: (1) blast furnace, (2) central maintenance, (3) finishing, (4) foundry, (5) masonry, and (6) melt shop. Out of these six departments, the melt shop (both task and representative workday samples) was the only one in this time period where the mean airborne asbestos concentrations added to the standard deviations or the 95th percentile values exceeded the contemporaneous PEL (Tables 4 and 5).

For the melt shop, there were 28 samples collected between 1972 and 1975. The mean of the representative workday samples (*n* = 12) was 3.10 f/cc and the standard deviation was 3.08 f/cc (Table 5). The mean of task samples (*n* = 16) was 4.23 f/cc and the standard deviation was 6.32 f/cc (Table 4). Although the means of both the representative workday and task samples in the melt shop from 1972 to 1975 were below the contemporaneous PEL of 5 f/cc, the mean added to the standard deviation for both sample types exceeded 5 f/cc. There were two representative workday samples and five task samples that exceeded the contemporaneous PEL of 5 f/cc (Fig. 3b). These seven PEL exceeding samples were of moldmen at Fairless and Gary Works, some of which were noted to not be following the proper procedures for cleaning molds [12, 124].

Excluding the melt shop samples from 1972 through 1975, there were five departments where representative workday samples were collected during this time period. For samples in these five departments (*n* = 39), the mean of the representative workday samples (*n* = 33) was 0.36 f/cc, the standard deviation was 0.55 f/cc, and 95th percentile was 1.10 f/cc. Other than the melt shop, the blast furnace, central maintenance, finishing, and masonry departments had at least one task sample. The mean of task samples from these four departments (*n* = 6) during this time was 0.78 f/cc, the standard deviation was 0.71 f/cc, and 95th percentile was 1.79 f/cc.

### Airborne asbestos concentrations (1976–1985)

For samples from all facilities and departments in this time period (*n* = 203) the mean of representative workday samples (n = 105) was 0.13 f/cc, the standard deviation was 0.30 f/cc, and the 95th percentile was 1.11 f/cc (Table 1). For task samples (*n* = 98), the mean was 0.48 f/cc, standard deviation was 1.93 f/cc, and the 95^th^ percentile was 1.12 f/cc (Table 1). There was a total of three datapoints, all task samples, that were above the contemporaneous PEL of 2 f/cc. All occurred at the Fairless Works facility. One sample occurred in a coke oven patcher in the coke department [70]. The remaining two were central maintenance insulators removing pipe insulation, one of which was the maximum concentration from this time period and exceeded the OSHA 15-minute ceiling limit of 10 f/cc [70, 77]. These samples are described in detail in Appendix III*. PEL Exceeding Instances*.

### 1976–1985 results by facility

**Fig. 4:**
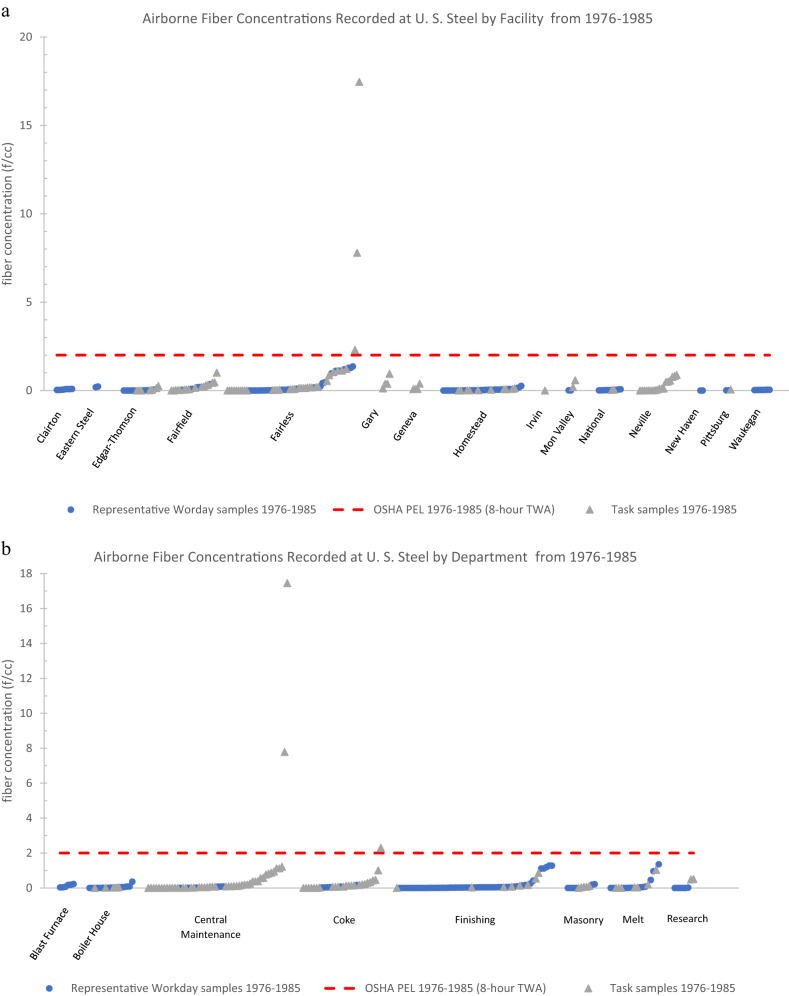
Airborne fiber concentrations recorded at U. S. Steel from 1976 to 1985. **a** Scatterplot of individual data points (representative workday samples are blue circles; task samples are gray triangles) from 1976 to 1985 across each facility for which asbestos air samples were recorded. Three datapoints from Fairless Works out of 203 total samples for this time period exceeded the contemporaneous PEL (red dotted line) of 2 f/cc. **b** Scatterplot of individual data points (representative workday samples are blue circles; task samples are gray triangles) from 1976 to 1985 across each department for which asbestos air samples were recorded. Three datapoints, two from the Central Maintenance department and one from the Coke department, out of 203 total samples for this time period exceeded the contemporaneous PEL (red dotted line) of 2 f/cc.

Figure 4a presents airborne asbestos concentrations based on personal samples collected from 1976 until 1985 by facility. Data were collected at 15 facilities during this time: (1) Clairton Works, (2) Eastern Steel Division, (3) Edgar Thomson Plant, (4) Fairfield Works, (5) Fairless Works, (6) Gary Works, (7) Geneva Works, (8) Homestead Works, (9) Irvin Works, (10) Mon Valley Works, (11) National Duquesne Works, (12) Neville Island Plant, (13) New Haven Works, (14) Pittsburg Works, and (15) Waukegan Works. Out of these 15 facilities, Fairless Works was the only facility in this time period with reported concentrations (three values) that exceeded the contemporaneous PEL (Fig. 4a).

For Fairless Works, there were 62 airborne asbestos personal samples collected between 1976 and 1985. The mean of these representative workday samples (*n* = 27) was 0.37 f/cc, the standard deviation was 0.51 f/cc, the 95th percentile was 1.28 f/cc, and the maximum concentration was 1.36 f/cc (Table 3). The mean of the task samples from Fairless Works from 1976 to 1985 (*n* = 35) was 1.01 f/cc, the standard deviation was 3.17 f/cc, the 95th percentile was 7.79 f/cc, and the maximum concentration was 17.46 f/cc (Table 2). Although the mean is below the contemporaneous PEL of 2 f/cc, the mean added to the standard deviation, 95th percentile, and maximum concentration of task samples exceeds the PEL. This was because of three PEL exceeding samples. One sample occurred in a coke oven patcher in the coke department [70]. The remaining two were central maintenance insulators removing pipe insulation, one of which was the maximum concentration from 1976 to 1985 and exceeded the OSHA 15-minute ceiling limit of 10 f/cc [70, 77].

Excluding Fairless Works samples from 1976 through 1985, there were 14 facilities where samples were collected during this time (*n* = 141). For the representative workday samples (*n* = 78), which appear in 11 of these facilities, the mean was 0.05 f/cc, the standard deviation was 0.07 f/cc, and the 95th percentile was 0.19 f/cc. For task samples (*n* = 63), which appear in 10 of these facilities, the mean was 0.19 f/cc, standard deviation was 0.26 f/cc, and the 95th percentile was 0.82 f/cc.

### 1976–1985 results by department

Figure 4b presents airborne asbestos concentrations based on personal samples collected from 1976 until 1985 by department. There were eight departments sampled during this period: (1) blast furnace, (2) boiler house, (3) central maintenance, (4) coke, (5) finishing, (6) masonry, (7) melt shop, and (8) research lab. Out of these eight departments, none of the mean concentrations of either task or representative workday sample exceeded the PEL. Central maintenance task samples were the only group in this time period where the mean airborne asbestos concentration added to the standard deviations exceeded the contemporaneous PEL (Table 3).

For the central maintenance department, there were 53 samples collected between 1976 and 1985 (47 task samples and 6 representative workday samples). The mean of these task samples was 0.77 f/cc and the standard deviation was 2.74 f/cc (Table 4). The mean plus the associated standard deviation would have exceeded the contemporaneous PEL of 2 f/cc. However, the 95th percentile value of 1.22 f/cc was below the 2 f/cc PEL (Table 4). The six representative workday samples from the central maintenance department during this time period had a mean of 0.05 f/cc and standard deviation of 0.04 f/cc, with a maximum value of 0.09 f/cc (Table 5).

This time period had three PEL exceeding instances (Fig. 4b). Two of these instances, both in insulators at Fairless Works (Fig. 4a) contributed to the large standard deviation in the central maintenance department task samples during this time [70, 77]. The remaining exceedance occurred in a patcher helper from the coke department [70].

Excluding central maintenance department task samples from 1976 through 1985, there were six departments where task samples were collected during this time period (there were no task samples in the blast furnace department during this time period). For these task samples (*n* = 51), the mean was 0.21 f/cc, the standard deviation was 0.38 f/cc, and the 95th percentile was 1.01 f/cc.

### Airborne asbestos concentrations (1986–1993)

**Fig. 5:**
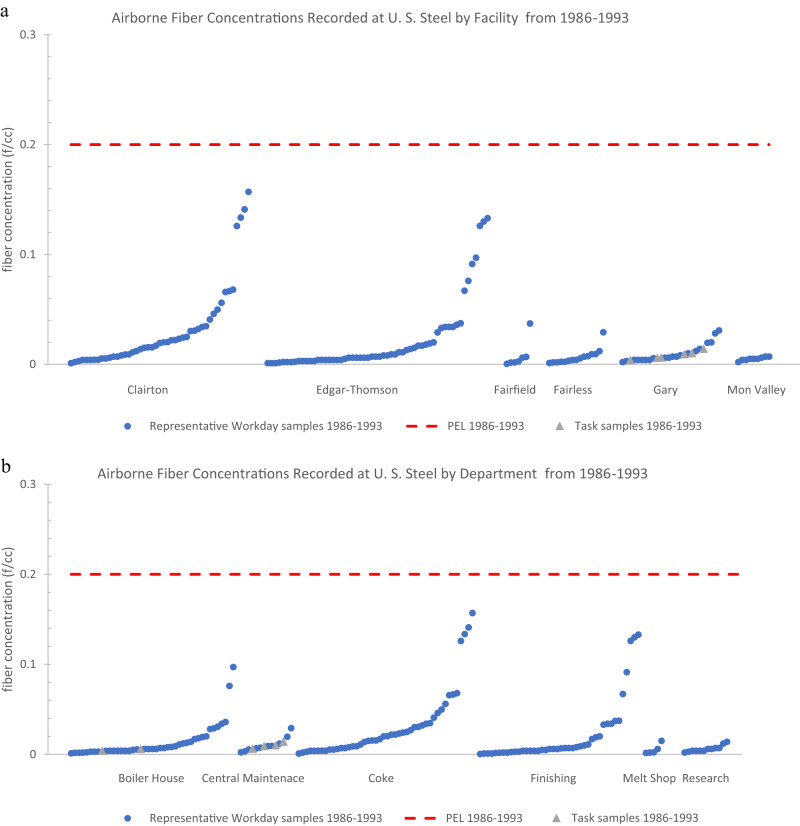
Airborne fiber concentrations recorded at U. S. Steel from 1986 to 1993. **a** Scatterplot of individual data points (representative workday samples are blue circles; task samples are gray triangles) from 1986 to 1993 across each facility for which asbestos air samples were recorded. None of the 162 samples exceeded the contemporaneous PEL (red dotted line) of 0.2 f/cc. **b** Scatterplot of individual data points (representative workday samples are blue circles; task samples are gray triangles) from 1986 to 1993 across each department for which asbestos air samples were recorded. None of the 162 total samples during this time period exceeded the contemporaneous PEL (red dotted line) of 0.2 f/cc.

For both task and representative workday samples, none of the mean asbestos air concentrations, mean added to the standard deviation, or 95th percentiles for any facility during this the time exceeded the contemporaneous PEL of 0.2 f/cc (Tables 2 and 3). Additionally, no individual datapoint exceeded the contemporaneous PEL from 1986 through 1993 (Fig. 5a). For samples from all facilities in this time period (*n* = 158), the mean of representative workday samples (*n* = 152) was 0.02 f/cc, standard deviation was 0.03 f/cc, and the 95th percentile was 0.10 f/cc (Table 1). For task samples (*n* = 6), the mean was 0.01 f/cc and the standard deviation was 0.003 f/cc (Table 1).

### 1986–1993 data by facility

Figure 5a presents airborne concentrations based on personal samples from 1986 until 1993 by facility. Data were collected at six facilities during this time: (1) Clairton Works, (2) Edgar Thomson Plant, (3) Fairfield Works, (4) Fairless Works, (5) Gary Works, and (6) Mon Valley Works.

### 1986–1993 data by department

Figure 5b presents airborne asbestos concentrations based on personal samples collected from 1986 until 1993 by department. There were six departments where sampling occurred during this period: (1) boiler house, (2) central maintenance, (3) coke, (4) finishing, (5) melt shop, and (6) research lab.

### Airborne asbestos concentrations at the Gary Works facility (1994–2006)

**Fig. 6:**
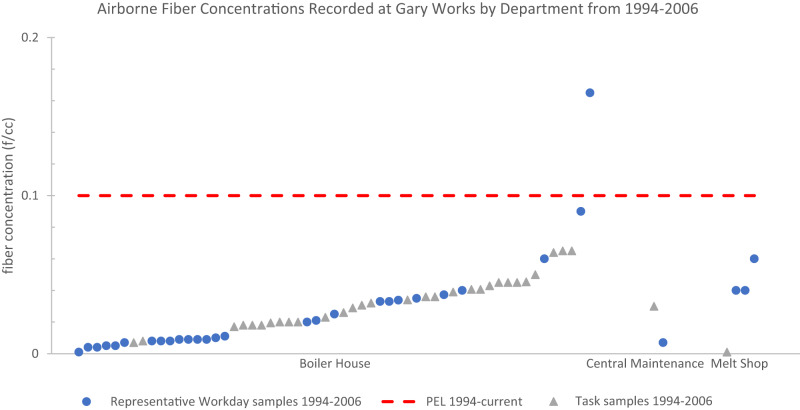
Airborne fiber concentrations recorded at Gary Works from 1994 to 2006. Scatterplot of individual data points (representative workday samples are blue circles; task samples are gray triangles) from 1994 to 2006 across each department for which asbestos air samples were recorded at the Gary Works facility. This was the only facility in which data were available during this time period. One (in the Boiler House) of the 63 total samples during this time period exceeded the contemporaneous PEL (red dotted line) of 0.1 f/cc.

Figure 6 presents airborne asbestos concentrations based on personal samples from 1994 until 2006 at the Gary Works facility. This was the only facility sampled during this time period.

For samples collected at Gary Works during this time period (*n* = 67) the mean of representative workday samples (*n* = 35) was 0.03 f/cc, the standard deviation was 0.03 f/cc, and the 95th percentile was 0.09 f/cc (Table 1). For task samples (*n* = 32), the mean was 0.03 f/cc, the standard deviation was 0.02 f/cc, and the 95th percentile was 0.07 f/cc (Table 1). The mean of both task and representative workday samples added to their respective standard deviations did not exceed the contemporaneous PEL of 0.1 f/cc. One datapoint exceeded the contemporaneous PEL of 0.1 f/cc from 1994 to 2006 (Fig. 6). This representative workday sample was collected while a utilityman was working at the Gary Works facility’s boiler house and had a TWA concentration of 0.17 f/cc [132].

### 1994–2006 data by Department

There were three departments where sampling occurred during this period: (1) boiler house, (2) central maintenance, and (3) melt shop.

None of the mean asbestos air concentrations, mean added to the standard deviation, or 95th percentiles for any department (both task and representative workday samples) during this time period exceeded the PEL of 0.1 f/cc (Tables 4 and 5). One individual sample exceeded the contemporaneous PEL during this time (Fig. 6). This sample was collected while a utilityman was working at the Gary Works facility’s boiler house and had a TWA concentration of 0.17 f/cc [132].

## Discussion

This paper presents all the available personal sampling data for airborne asbestos from 16 U. S. Steel facilities between 1972 and 2006. This represents one of the most robust industrial hygiene datasets from any steelmaking corporation examined in the published literature. Our analysis reinforces the notion that there was a limited opportunity for exposure to asbestos at U. S. Steel in the post-OSHA era. These results may apply, to a large degree, to other steel making and some basic metal production corporations. The industrial hygiene sampling conducted by U. S. Steel is not unlike the campaigns that were conducted by other industrial companies who attempted to ensure that they were complying with U.S. regulatory expectations following the passage of OSHA and in particular, asbestos regulations.

### Highest fiber concentrations in a steel mill

One of the benefits of this paper is that it provides insight into where and how ACMs were used throughout the steel making processes. Heretofore, no single resource was available that focused on this subject in its entirety. An additional benefit of our current paper is that it provides an understanding of the various job classifications that are involved in steel making where historical exposure to asbestos was plausible.

The highest recorded fiber concentrations typically occurred in either moldmen cleaning ingot molds or employees interacting with pipe insulation. These two instances account for 10 of the 11 OSHA PEL (as an 8-hour TWA) exceeding instances in the dataset. No other task had multiple samples with concentrations above the contemporaneous PEL. Only one of these 10 samples was truly a representative workday TWA, the remaining samples were task samples (*n* = 7) or had unknown sampling times (*n* = 2).

Moldmen cleaning molds in 1973 at Fairless Works and Gary Works facilities were the only PEL exceeding instances (*n* = 7; five task samples and two samples with unknown sampling times) in the data from 1972 to 1975. The only ACMs that seem relevant for these samples appear to be hot top boards and liners. The asbestos-containing hot top boards and liners used at U. S. Steel were replaced with non-asbestos alternatives by 1976 or earlier (see section *Handling Hot Tops and Cleaning Ingot Molds)*. Exposures while cleaning the hot tops occurred after molten steel was poured which causes these boards/liners to be consumed by the liquid steel. Due to these high temperatures, we expect that the fibers detected in these samples were harmless silicates, which are the byproduct of thermally degraded asbestos. Records indicated that this task would have involved the use of respirators and that vacuuming with proper capture bags (rather than sweeping or the use of compressed air) was required, especially after U. S. Steel industrial hygienists had conducted this sampling campaign in 1973 (see Appendix III*. PEL Exceeding Instances*). The use of ingot teeming (and therefore the need to clean molds) gradually became obsolete with the introduction of continuous casting, which became the most popular steelmaking method in the 1980s [128].

Two insulators removing old pipe insulation in 1978 had short-term exposures above 5 f/cc. At least one of these employees had reportedly failed to wet materials, which was the required practice at U. S. Steel (see Appendix III*. PEL Exceeding Instances*). Both employees would have been required to wear respirators for this task. After the early 1980s, the removal of potential asbestos-containing insulation was performed by licensed abatement workers. The only PEL exceeding concentration in the dataset after 1978 occurred in 2001 for a utilityman. The utilityman had a TWA of 0.17 f/cc over 241 minutes. The only plausible ACM near the employee was pipe insulation, although the industrial hygienist noted he was not permitted to interact with it.

### The exposure of oversight employees

U. S. Steel sampled the staff whose main duties consisted of oversight responsibilities (e.g., foreman, superintendent, team leader, group leader, coordinator, process observer, tool room attendant, and manager). These workers had the potential to be exposed to asbestos as bystanders.

The mean task sample fiber concentration for oversight employees in the entire dataset was 0.01 f/cc with a standard deviation of 0.02 f/cc (*n* = 5). The mean representative workday asbestos fiber concentration for oversight employees across all time periods was 0.03 f/cc with a standard deviation of 0.03 f/cc and a maximum recorded concentration of 0.14 f/cc (*n* = 19). The mean representative task concentration for oversight employees across all time periods was 0.01 f/cc with a standard deviation of 0.02 f/cc and a maximum recorded concentration of 0.04 f/cc (*n* = 5). Asbestos air sampling for oversight employees, when grouped by department, tended to be less than the values of those performing hands on work.

The lesser exposure to asbestos among oversight positions, who are less frequently involved in hands-on work compared to other roles, is to be expected as studies have demonstrated a clear and predictable decrease in the airborne concentration of asbestos fibers with distance from a source [133, 134]. Donovan et al. [133] found that for persons 1 to 5 feet from the source, airborne asbestos concentrations can be roughly approximated as 50% of the source concentration; 35% between 5 and 10 feet, 10% between 10 and 30 feet, and less than 1% at distances greater than 30 feet. Our data follows this trend as the mean airborne asbestos concentration of representative workday samples for oversight employees was 12.3% of the mean for all other employees from 1972 to 2006 and the mean concentration of task samples for oversight employees was 1.6% of the mean concentration for all other employees.

### The effectiveness of engineering controls and PPE

Based on our reading of the available U. S. Steel industrial hygiene document, this corporation was aware of the hazards of asbestos based on the knowledge available at the time. They continued to modify and improve their safety practices over time. Additional engineering controls (i.e., ventilation systems and the use of non-asbestos-containing products when possible) and work practices (i.e., wetting of ACMs, labeling ACMs, housekeeping, and the use of personal protective equipment) were put in place in response to both regulations and industrial hygiene monitoring.

Some of these efforts are reflected by the trends in the data observed across time periods. There are significant decreases in fiber concentrations for both task and representative workday samples across the first three time periods (1972–1975, 1976–1985, and 1986–1993) until fiber concentrations begin to level off in the last time period (1994–2006) (Fig. 1 and 2). Out of the 11 PEL exceeding instances, 10 of them occurred prior to 1978.

It is known, based on available U.S. Steel industrial hygiene reports, that respirators were routinely worn [135–137]. If the workers wore the appropriate respirator and it was properly fitted, then it can be expected to have provided a level of protection corresponding to its applied protection factor (APF) [138]. For example, several U.S. Steel industrial hygiene reports mentioned workers being recommended quarter and half mask respirators [70, 119, 139]. The APF for quarter masks is 5 and the APF for half masks is 10 [140]. These APFs indicate a 5 to 10-fold reduction in the concentration of the contaminant in the respirator facepiece compared to the ambient environment where samples were taken [138].

### Sampling campaigns vs typical exposures

Because U. S. Steel’s industrial hygiene sampling campaigns were frequently focused on tasks where there was some anticipation of over-exposure to asbestos, as many were the result of inquiries by the United Steelworkers labor union [141–144], the data presented in this paper almost certainly overestimates typical exposures of most employees. That is, tasks or jobs that were believed to have had the potential for exposure to asbestos exceeding the PEL are likely overrepresented in the dataset. Based on the limited use and types of ACMs in a steel mill, it is not surprising that exposures, even when sampling was focused on ‘expected high exposures’, were typically below the PEL.

Additionally, air concentrations that were compared to the PEL may not necessarily represent the employee exposures. For example, the use of respirators for certain workers and tasks per U. S. Steel’s policies, would have reduced exposures by the respirator’s APF. This reduction was not accounted for in our analysis.

### Comparing samples to the contemporaneous OSHA PELs

When one evaluates these data, it needs to be recognized that historically the industrial hygiene community generally believed that if a company could control exposures to be below either the ACGIH TLV or the OSHA PEL, then the workers were likely not to be at an unacceptable risk of developing an occupational disease [145, 146]. It was only when questions were raised about whether low dose cancer models should be used to evaluate the acceptability of exposure did persons question whether these occupational exposure limits were adequately protective [147, 148]. Since 1975, the OSHA PEL for asbestos was determined using a linearized no threshold (LNT) model [149]. The LNT model is a dose-response model that only assumes there is no level of exposure where there is zero risk. The current OSHA PEL for asbestos of 0.1 f/cc estimates a lifetime risk of death from asbestos related cancer of 3.4 per 1000 workers and a 20-year exposure risk of 2.3 per 1000 workers [30, p. 40966–40967]. Of course, the OSHA PEL is not entirely dependent on the results of low dose models, as it considers economic feasibility and cost-benefit analyses [150].

For our analysis, all of the individual sample results as well as the mean, mean added to the standard deviation, and 95th percentiles were compared to the contemporaneous OSHA PELs for asbestos. The PELs were developed for exposures on an 8-hour TWA basis [30]. Representative workday samples, either those with adequate sampling time or calculated 8-hour TWAs, can appropriately be compared to these PELs. Comparing task samples, which were sampled for less than 180 minutes and were used to capture short-term exposures, to the PEL was a conservative assumption in our analysis.

Three out of the 11 PEL exceeding samples in the dataset were considered representative workday samples. Only one sample had a sampling time greater than 180 minutes [151]. The remaining two had unknown sampling times, and there was not enough information provided to assume these samples were not representative of an employee’s entire workday [12].

### Limitations of PCM sampling

The NIOSH method 7400 (PCM analyses), as was used in the available U. S. Steel industrial hygiene reports, counts all fibers (e.g., asbestos, olivine, fiberglass, cotton fibers, etc.) that are greater than 5 µm in length with at least a 3:1 aspect ratio [152]. This method is not specific to asbestos fibers. Based upon the high temperatures present during certain steelmaking operations, asbestos fibers would be converted to olivine (i.e., forsterite) or other harmless silicates [52]. Based on discussions with an expert in the field of microscopy, olivine fibers would be morphologically indistinguishable from asbestos fibers (personal communications with Dr. Bryan Bandli). We believe that many non-asbestos fibers were counted in the sample results as asbestos; however, our data analysis conservatively assumed that all reported concentrations were asbestos.

TEM analysis, unlike PCM analysis, can differentiate between asbestos and non-asbestos fibers. Some TEM analysis was conducted at the Gary Works facility in 1997 or later. Several industrial hygiene reports found that sampling filters contained no asbestos, but they did contain non-asbestos fibers that would have been counted under PCM analysis [153–155]. Additionally, in 1999 an outside accredited laboratory used TEM to reanalyze a U. S. Steel industrial hygiene sample from the year before. This sample was identified as having a relatively high fiber concentration from PCM analysis [156]. The TEM reanalysis of this specific sample found that no asbestos fibers were detected [157].

It is the author’s opinion that, had TEM analysis originally conducted on all of the available U. S. Steel industrial hygiene samples, the measured asbestos concentrations would have likely been significantly lower since non-asbestos fibers (i.e., forsterite, fiberglass, and clothing fibers) were apparently reported. As such, we expect our results likely overestimated the actual asbestos exposures to U. S. Steel employees.

### Comparison to the textbook in-plant practices for job related health hazards control (1989)

In the book *In-Plant Practices for Job Related Health Hazards Control (1989)*, hazards in various industries are discussed based on the hands-on experience of certified industrial hygienists. The chapter on steelmaking had limited discussion specific to asbestos. The authors of that chapter noted that, in 1989, asbestos use in steel mills was limited to equipment brakes and could potentially be found on old pipe insulation [158, pg. 776–777]. Also, the authors conducted a Pareto analysis prioritizing industrial hygiene hazards in this industry. This analysis ranked asbestos 15^th^ out of 20 agents considered. The authors stated that “… in an integrated steel plant, noise, carbon monoxide, heat, coke oven emissions, and chlorinated solvents rank at the top priority of hazard index [158, pg. 779].” The authors additionally noted that:

“[i]ron and steel presents a broad variety of industrial hygiene concerns … The industry has handled its responsibilities well and, as a rule, has not experienced an inordinate amount of occupational-exposure-related disease. Exposures are typically well in control. This is partially because the industry has established a solid base of industrial hygiene expertise and services [158, pg. 778].”

Our analysis agrees with this text as it indicates there was a limited opportunity for exposure to asbestos at U. S. Steel in 1989 and well prior.

### Comparison to Hollins et al. [100]

Hollins et al. characterized 138 asbestos personal air samples from 1972 to 1982 for bricklayers and other tradesmen during furnace and stove relining at three steel mills [100]. Average airborne fiber concentrations during relining of open-hearth furnaces, stoves, and blast furnaces were 0.21 f/cc (SD = 0.37 f/cc), 0.72 f/cc (SD = 0.72 f/cc), and 0.13 f/cc (SD = 0.19 f/cc), respectively [100]. Airborne fiber concentrations of the four samples that had sampling times greater than 227 minutes averaged 0.045 f/cc (SD = 0.017 f/cc) [100]. They estimated that the 8-hour TWA concentrations for bricklayers had an upper bound of 0.34 f/cc and for bricklayer’s helpers had an upper bound of 0.20 f/cc [100].

In the dataset used for this paper, there were 17 samples collected between 1972 and 1985 for bricklayers and bricklayer’s helpers. For representative workday samples (*n* = 11), these data had an average asbestos concentration of 0.14 f/cc with a standard deviation of 0.29 f/cc (Table 18). Task samples (*n* = 6) had similar concentrations with a mean of 0.11 f/cc and standard deviation of 0.12 f/cc (Table 17). Our results were consistent with the findings of Hollins et al. [100] in that exposures during the relining of melting vessels did not exceed the relevant contemporaneous occupational exposure limits. Their data, which were collected at different firms, supports that asbestos exposures were consistent across different steel making companies.

### Epidemiological studies of asbestos-related diseases

There are several reports that select steelworkers may be at an increased risk of developing mesothelioma [159, 160]. Roggli et al. [159] found that for steel/metal workers (which included steel, aluminum and iron foundry workers, furnace workers, and pot room workers) there were 33 cases of mesothelioma out of the 1445 mesothelioma cases (approximately 2.3% of total cases) that were examined. It should be noted that the authors did not distinguish how many of the observed mesothelioma deaths were in steelworkers, compared to the other listed occupations that were combined under the steel/metal workers category. A 2017 Centers for Disease Control and Prevention (CDC) Morbidity and Mortality Weekly Report (MMWR) report stated that between 1999 and 2015, there were 45,221 deaths in 23 states where malignant mesothelioma was the underlying or contributing cause of death listed on the death certificate [160]. From the death certificates, the authors noted that during this time period, there were ten mesothelioma deaths from structural iron and steel workers (approximately 0.022% of total cases), with a proportional mortality ratio of 3.3 (95% CI 1.6–6.0) [160]. Based upon the generally accepted 30-to-40-year latency of mesothelioma [160], the 2017 MMWR report should include steelworkers who worked from approximately 1960 through the mid-1980s. From the dataset analyzed in our paper, 54.5% of the samples were collected at U. S Steel in 1985 or earlier (Table 1).

These report findings are supported by a series of articles that were produced via a collaboration that began in 1962 between the U.S. Public Health Service, the University of Pittsburgh, Graduate School of Public Health and three major steel corporations in order to identify potential hazards in the steel making process [161]. This cohort comprised 59,072 steel workers in seven plants located in Allegheny County, Pennsylvania and represented approximately 62% of all men working in basic iron and steel production in the country in 1953 [161]. This collaboration produced a series of ten journal articles discussing mortality by occupation within the steel trade which were published from 1969 to 1976 [161–170], and a 1981 NIOSH report [171]. Follow-up articles on lung cancer mortality and risk for coke oven operators were published in 1975, 1983 and 1995 [172–174]. None of these 14 epidemiology studies published on this steelworker cohort showed an increased incidence of asbestos-related diseases, including mesothelioma.

It should also be noted that while the link between some types of “asbestos” exposure and mesothelioma has been clearly established, it is simultaneously true that there are a significant number of non-asbestos-related mesothelioma cases occurring spontaneously with no identifiable history of asbestos exposure. It is well-established in the scientific literature that mesothelioma occurs in the general population at a fairly constant rate in the absence of asbestos exposure [175–180]. The Surveillance, Epidemiology, and End Results (SEER) data have also suggested an age-related increase in the incidence of mesothelioma [180–184]. In 2022, an update to the Price and Ware article used the most current SEER data from 1975 through 2018 [184]. Those authors stated that based on that dataset, the estimated lifetime background risk of mesothelioma is between 3.8 and 4.3 per 100,000 [184]. They estimated that after 2040, there will be no additional asbestos-related mesothelioma cases and all of the cases thereafter can be considered non-asbestos-related mesotheliomas [184]. Additionally, there is evidence that mesothelioma can occur in individuals exposed to non-asbestos mineral fibers (i.e., erionite), therapeutic radiation [185–190], and inflammation not associated with asbestos fibers [191, 192].

## Conclusion

For both task samples and representative workday samples, none of the mean airborne asbestos fiber concentrations by facility or department exceeded the contemporaneous PEL for the time period in which they were sampled (Tables 2 and 3; Tables 4 and 5). The only job category within a department for any time period, out of 64 possible job categories, which had a mean concentration that exceeded the contemporaneous PEL for asbestos were insulators in the central maintenance department from 1976 to 1985 (*n* = 5). Eight of the eleven contemporaneous PEL exceeding samples were task samples and are believed to likely not have been representative of average workday exposures. Of the three representative workday samples in the dataset that exceeded contemporaneous PEL, two had unknown sampling times and were conservatively categorized as representative workday samples in our analysis. Additionally, it was likely that due to the analytical methods used, the measured concentrations in the samples were not comprised exclusively of asbestos for many of these PEL exceeding instances. Such employees were generally protected from these PEL exceedances by a respiratory protection program. This further emphasizes that exposures to asbestos that were in excess of the PEL at the time in the steelmaking industry was particularly rare as only 0.9% of representative workday samples exceeded the contemporaneous PEL over this 34-year time period. When compared to modern OELs, 16.6% of representative workday samples from 1972 through 2006 exceeded the current PEL of 0.1 f/cc. Asbestos exposure control methods improved and the use of ACMs decreased across this sampling period. The effectiveness of these measures is clear as only 9 out of 213 (4.2%) representative workday samples past 1980 exceeded 0.1 f/cc. Overall, the data and the information contained within U. S. Steel’s available industrial hygiene documents and reports between 1972 and 2006 indicated that U. S. Steel’s industrial hygiene department were aware of the hazards of asbestos and conducted an active management program to understand and control the airborne concentrations in their workplaces.

### Supplementary information

Supplementary information

## Data Availability

It has been represented to the authors that the reports upon which the data were obtained are available on request from the United States Steel Corporation.

## Appendix I. Job groupings

Job categories were decided based on job tasks and descriptions that were used in the available U. S. Steel industrial hygiene reports, as well as consultation with former employees and industrial hygienists at steel manufacturers to group job titles that involved similar plausible exposures to ACMs.

## Appendix II. Summary of industrial hygiene report datasets that were removed or corrected

*U. S. Steel Internally Corrected Data*

The data from one sampling event contained mathematical errors that were corrected internally by industrial hygienists at U. S. Steel prior to our analysis. In a January 9th, 1979, report from the Fairless Works facility, we are aware that one of the industrial hygienists erroneously reported values between 88.64 f/cc to 2,790.72 f/cc as an 8-hour TWA [193]. Such a concentration is not feasible and was not considered in the data analysis. This was also discovered by U. S. Steel, and there was a corrected version of this dataset [77] to amend these calculation errors. These corrected data were used in our analysis.

*Author Corrected Data*

There were two industrial hygiene reports identified by the authors as containing mistakes that were not corrected internally by U. S. Steel. The entry of these data into our proprietary databases is explained below.

An October 25th, 1978, industrial hygiene report that presented analytical results that contributed 17 datapoints to our analysis [70] clearly contained a mistake in whether the data were representative or task samples. As such, different methods were used in the determination of whether a sample was a representative workday sample or a task sample. In the introduction to this report, the industrial hygienist wrote “[t]he analytical results listed below are calculated by converting the short-term sample results to eight (8) hour time weighted average (TWA) concentrations.” This led us to believe that the presented results were 8-hour TWAs and would therefore be representative workday samples. However, there were four samples that were marked with an asterisk (*) or double asterisk (**). For these samples, the hygienist noted: “Results denoted by asterisk (*) are for 5 min of sampling. These results do not exceed the 5 min ceiling limit, but do exceed the 8 h TWA limit. Results denoted by Double asterisk (**) are also for 5 min samples, but the results exceed both the 5 min ceiling limit and the 8 h TWA limit” [70, p. 4].

Two of the four marked results (0.08 f/cc and 0.10 f/cc) did not adhere to these described criteria, as they were below the 8-hour TWA limit of 2 f/cc in 1978. In our best judgment, we considered all of the datapoints in this report that did not carry asterisks (*n* = 13) as representative workday samples and those with an asterisk (*n* = 4), since they were specified as being 5-minute samples, were considered task samples.

In a separate January 3rd, 1979, report from the Fairless Works facility, we are aware that one of the industrial hygienists erroneously reported 8-hour TWA values of 59.30 f/cc, 55.47 f/cc, and 73.60 f/cc [194]. Such concentrations are not feasible based on the provided short-term data that was used to calculate them (1.36 f/cc for 11 min, 1.04 f/cc for 9 min, and 0.46 f/cc for 3 min). The 8-hour TWA calculations were not considered in our statistical analysis; however, there was no reason to believe the short-term sampling values themselves were inaccurate. As such, these short-term samples, which were provided in the report, were included in our dataset as task samples.

*Author Excluded data*

A total of three industrial hygiene reports were excluded from our proprietary database and thus, were not analyzed. All removed data consisted of samples that were either non-U. S. Steel employees performing abatement work or clear analytical or reporting errors that we believed were invalid.

In an April 11th, 1985, report from the Edgar-Thomson Works facility, one out of six samples recorded, which had sampling times between 421 and 444 min, exceeded the PEL of 2 f/cc [21]. This sample was of a Pump Tender at the number 2 power house. This worker had a measured concentration of 8.6 f/cc, while another pump tender in this report three days later had a reported concentration of only 0.005 f/cc [21]. In addition to the two pump tenders, this report sampled two boiler house group leaders and had two samples for a fan tender. Overall, the values from this report were 0.013 f/cc, 0.009 f/cc, 0.05 f/cc, 0.005 f/cc, 8.6 f/cc, and one sample in which no fibers were detected [21]. With the anomalous sample removed, the grand mean airborne concentration of fibers was 0.015 f/cc (*n* = 5). In our opinion, this relatively high concentration sample is not considered reliable and likely involved an analytical or reporting error as it was over 1,000-fold higher than similar samples in the report [21].

In a May 30th, 1985, report out of Fairless Works, an abatement contract worker was sampled over 240 minutes while removing asbestos [195]. The 8-hour TWA was calculated to be 3.4 f/cc, which exceeded the PEL of 2 f/cc [195]. This employee was not a U. S. Steel employee, but a contract workers hired to remove asbestos-containing pipe insulation. The worker from this sample wore a respirator [195].

In a Fairless Works report from August 10th, 1988, a group of abatement workers from the North Tin Mill were sampled [20]. These employees were not U. S. Steel employees but contract workers from a company hired to remove asbestos-containing pipe insulation from a 124-foot section in the mill [20]. The abatement process presented an opportunity for asbestos exposure. For this reason, the abatement process consisted of building a plastic enclosure around the area to prevent asbestos fibers in the air from escaping and traveling elsewhere in the plant. All area samples collected around the outside of the enclosure yielded a fiber concentration below 0.01 f/cc [20]. Workers within the enclosure wore respirators and Tyvek suits.

## Appendix III. PEL exceeding instances

There were 11 instances of datapoints that exceeded the contemporaneous PELs for asbestos between 1972 and 2006 out of the total 495 personal samples analyzed (2.2%). When evaluating industrial hygiene data, it can be useful to ‘dig a little deeper’ when you examine the circumstances on the day that the sampling took place. These 11 instances, eight of which are task samples, appear in five reports and are discussed below [12, 70, 77, 124, 196].

*III.1 Moldmen Cleaning Molds at Fairless Works (n* = *2) and Gary Works (n* = *5) in 1973*

Seven of the 11 PEL exceeding samples involved moldmen cleaning molds in the melt shop at Fairless Works [12] and Gary Works [124] in 1973. These can be seen in Fig. 3a. As discussed previously, based on the high temperatures of molten steel during the pouring of it into ingot molds, it is logical to assume that asbestos from hot top products would have been converted into silicates like olivine (i.e., forsterite) after the initial pour [52, 126]. We believe these non-asbestos fibers were counted as asbestos fibers for these moldmen PCM samples.

It was reported that in 1977 the use of asbestos-containing hot top materials, which are assumed to be the only possible source of airborne fibers (asbestos or olivine) for moldmen, had ceased [54]. This matches trends observed in our dataset as airborne concentrations of asbestos for moldmen in the melt shop department decreased from a mean of 4.57 f/cc with a standard deviation of 5.51 f/cc between 1972 and 1975 (*n* = 22) to a mean of 0.01 f/cc between 1976 and 1985 (*n* = 1) (Table 19). It should be noted, however, that the number of moldmen samples from these two periods decrease from 22 samples during 1972 to 1975 to only one during 1976 to 1985 (Table 20). In the melt shop, the overall mean fiber concentration from 1972 through 1975 was 3.75 f/cc with a standard deviation of 5.13 f/cc (*n* = 28) and from 1976 through 1985 was 0.22 f/cc with a standard deviation of 0.42 f/cc (*n* = 19) (Table 3).

*Details from Fairless Work’s Industrial Hygiene Report Regarding Moldmen PEL Exceedances*

Based upon the available industrial hygiene reports at the Fairless Works facility, there were two PEL exceeding samples involving the same moldman on back-to-back days in 1973 [12]. Since no sampling time was provided for these samples, the two calculated TWAs (7.9 f/cc and 10 f/cc) were assumed to be representative workday samples. The OSHA contemporaneous ceiling limit of 10 f/cc was exceeded during this task [12]. This employee was vacuuming molds without the use of collection bags. The industrial hygienist noted that: “It should be mandatory that the vacuum cleaner used for cleaning the molds be operated only with the proper bag collector attached and intact … We were informed the vacuuming was done without the bag because the capacity of the cleaner was not sufficient to accomplish the task with the added resistance of a bag. If this is the case, a cleaner with a larger capacity should be utilized so that a collector bag can be attached to retain the fine material picked up” [12].

This U. S. Steel report also noted that “… respirators approved by the U.S. Bureau of Mines (schedule 21B) should be provided and worn by moldmen when working with materials containing asbestos” [12].

*Details from Gary Work’s Industrial Hygiene Report Regarding Moldmen PEL Exceedances*

Based upon the available industrial hygiene reports at the Gary Works facility, there were five samples involving moldmen at the #2 and #3 hot top houses in 1973 that exceeded the contemporaneous PEL of 5 f/cc [124]. There were two task samples in the #2 hot top house that had recordings of 23.8 f/cc (40-minute sampling time) and 13.7 f/cc (42-minute sampling time), which were both greater than the 15-minute ceiling value of 10 f/cc. The third sample in the #2 hot top house that exceeded the 5 f/cc PEL was 6.7 f/cc (13-minute sampling time). Two short-term samples from the #3 hot top house that were PEL exceeding were 5.7 f/cc (1-minute sampling time) and 5.8 f/cc (39-minute sampling time). As previously discussed, we believe that these PCM samples of moldmen likely counted silicates such as olivine (i.e., forsterite) fibers. The industrial hygienist noted that: “Twenty-one air tests were taken in Nos. 2 & 3 Hot Top Houses covering the various phases of operation. Of these tests, two were found to exceed presently established air standards. The offending operation involves blowing (cleaning) molds in the #2 Hot Top House with an air ejector device. This is a relatively short-term operation performed several times a shift where personnel are exposed to high dust contamination including asbestos fibers. The remaining air data ranged below airborne asbestos standards” [124].

Based on this description, the results presented in this report were clearly task samples. Although the 8-hour TWAs for these samples were not provided, the industrial hygienist explained that only two out of 21 samples from this report would have exceeded air standards, both of which occurred in the #2 hot top house [124]. At the #3 hot top house specifically, he stated “Two of the three tests exceed 5 fibers/cc, however, their average daily exposure falls within the 5 fiber/cc value when considering the remaining time of the shift with little to no exposure” [124].

The industrial hygienist believed only two samples from the #2 hot top house would have exceeded air standards from this sampling event, because he was comparing short-term samples to the short-duration ceiling limit and 8-hour TWAs to the PEL [124]. Although referenced, these 8-hour TWAs were not provided in the industrial hygienist’s report.

During our analysis, per our methods, even the task sampling data as it appeared in the report was compared to the PEL. This resulted in three additional short-term instances, one from the #2 hot top house and two from the #3 hot top house, for a total of five samples in this industrial hygiene report that exceeded the contemporaneous PEL of 5 f/cc.

As mentioned in the report, vacuum suction equipment with dust collecting auxiliaries was installed in #3 hot top house; however, this equipment operated unsuccessfully which is why blowing of the molds was performed. As previously mentioned, vacuuming was the industrial hygienist recommended method for cleaning molds [12]. This report and earlier reports from 1972 indicated that this task would have involved the use of approved respirators as of that year [124, 127].

*III.2 Coke Oven Patcher Helper Soaking Rope at Fairless Works in 1978 (n* = *1)*

In 1978, there was one sample of a coke oven patcher helper soaking asbestos rope at Fairless Works that exceeded the contemporaneous PEL of 2 f/cc [70]. This employee was evaluated for 5 minutes while performing this task and had an exposure of 2.3 f/cc; however, the industrial hygienist noted that this did not exceed the ceiling limit of 5 f/cc for a 5-minute sample [70]. It was also noted that respirators were worn for the employee involved in this instance [70]. There were no other available U. S. Steel industrial hygiene reports involving the soaking of asbestos rope across the entire database where exposures exceeding the contemporaneous PELs.

*III.3 Insulators Removing Pipe Insulation at Fairless Works in 1978 (n* = *2)*

There were two PEL exceeding samples recorded in 1978 in which central maintenance insulators were removing old pipe insulation at Fairless Works [70], [77]. A sampling time of 5 minutes was provided for the first insulator’s sample [70]. This sample had a concentration of 7.79 f/cc; which, in the professional judgment of the industrial hygienist, exceeded the 5-minute ceiling limit of 5 f/cc [70]. However, the OSHA ceiling limit of 10 f/cc was not exceeded. It was noted was that this employee had failed to wet the material as was required [70]. It was also noted that respirators were worn for the employee involved in this instance [70]. The selection of an appropriate respirator for this work would have reduced this insulator’s exposure.

The second insulator was sampled for one 10-minute and three 5-minute events, for a total of 25 minutes on the same day [77]. The industrial hygienist did not convert these samples into an 8-hour TWA. Per our methods, since these samples were recorded for one employee on the same day, a single TWA of 17.46 f/cc for the 25 minutes of sampling was entered in our database and considered a task sample. The industrial hygienist noted that this insulator generated exposures greater than the contemporaneous OSHA ceiling level of 10 f/cc in each of his four samples [77]. It was not stated in the industrial hygiene report whether the proper wetting of materials was performed by this employee during this task. As mentioned previously, by this time frame it was recommended practice at U. S. Steel that respirators be worn when working with asbestos-containing insulation [11, p. 2].

*III.4 Boiler Utilityman Suspected of Interacting with Pipe Insulation at Gary Works in 2001 (n* = *1)*

In 2001, there was a single sample of a boiler utilityman at Gary Works that exceeded the contemporaneous PEL [196]. The industrial hygienist who wrote this report suspected that damaged pipe insulation caused this employee’s exposure; however, they noted that this employee was not permitted to interact with any pipe insulation, and there should not have been any additional work taking place in the area [196]. It is unclear precisely how this utilityman was exposed. This utilityman had a representative workday TWA calculated by the industrial hygienist of 0.17 f/cc over a total of 241 minutes of sampling [196]. This exceeded the contemporaneous OSHA PEL of 0.1 f/cc. Three sampling events contributed to this TWA. These three events had sampling times of 96 minutes, 30 minutes, and 115 minutes and sampling concentrations of 0.402 f/cc, 1.099 f/cc, and 0.096 f/cc, respectively. The 30-minute sample exceeded the contemporaneous OSHA 30-minute excursion limit of 1 f/cc. None of the other boiler house samples from this report had concentrations that exceeded the PEL [196].

## Appendix IV. Geometric mean and geometric standard deviation

In this analysis, since each data grouping contains only positive values, the arithmetic mean is always greater than or equal to the geometric mean for the same group of data. Although the geometric mean and geometric standard deviation may be appropriate to discuss for groups that have sufficient sample size, so as not to underestimate exposures, the arithmetic means and arithmetic standard deviations were discussed throughout the paper.

The sample sizes required to determine whether GSD can be used to reliably predict variability depends on both the value of the GSD and the ratio of the 95th percentile value to the occupational exposure limit it is being compared. A table describing these approximate sample size requirements (for a 95% confidence interval) has been recreated from the AIHI textbook, *A Strategy for assessing and Managing Occupational Exposures* (Table B). If the conditions of this table are met, the authors of this paper believe it is reasonable to use the geometric standard deviation to determine variability. The same AIHI textbook claims that a GSD of 1.5 suggests low variability, 2.5 suggests moderate variability, and 3.5 or greater suggests high variability [197, Figure 8.1].

The tables from the results section have been recreated using the geometric mean and geometric standard deviation. The sample size, 95th percentile, and maximum values in each group are also presented (Tables C1–C22). Several of the GSDs in this analysis are above 3.5, however it should be noted that this frequently occurred in groups where all of the values in that group were well below OELs. The presence of non-detect samples (which appear in the data as either half of the reported LOD or 0.001 f/cc) and other unusually small values can contribute to high GSDs in a sample even when all values are well below PEL.

## References

[1] Tarr DG, Baldwin RE, Hamilton CB, Sapir A (1988). The steel crisis in the United States and the European Community: causes and adjustments. Issues in US-EC trade relations..

[2] Lockington JN, Webster DL. Steel. In: Cralley LV, Cralley LJ (eds). In-plant practices for job related health hazards control. vol. 1. John Wiley & Sons, Inc.; 1989. p. 735–91.

[3] Selikoff IJ, Churg J, Hammond EC (1964). Asbestos exposure and neoplasia. J Am Med Assoc..

[4] Industrial Hygiene—Health Services Division. Industrial hygiene survey at Worcester Works—Wire Operations. Industrial Hygiene—Health Services Division, Personnel Services Department: United States Steel Corporation; 1965. 1–41.

[5] McGannon HE (ed). The making, shaping, and treating of steel. 9th. U.S. Steel: Pittsburgh, Pennsylvania; 1971.

[6] Paxton H. Steel. In: Kirk-Othmer encyclopedia of chemical technology. John Wiley & Sons, Inc.; 2004. p. 1–78.

[7] Morse KM. Letter to Lester V. Cralley regarding recommendations and opinions. 1952.

[8] Lee RJ, Lally JS, Fisher RM. Design of a laboratory for particulate analysis. 1981.

[9] Occupational Safety and Health Administration (OSHA). 1971. Emergency Standard for Exposure to Asbestos Dust. Fed. Reg. 235. p. 23207–8.

[10] Morse KM. Threshold limit value for asbestos dust exposure—Federal Occupational Safety and Health Act (OSHA). Recipient: General Superintendents and Plant Managers Superintendents—Personnel Services Supervisors of Safety, 1971:1–3.

[11] Morse KM. Interorganization Correspondence to General Superintendents, Plant Managers and Atendees at OSHA Regional Meetings Regarding Proposed Permanent Asbestos Standard. 1972. 1–7.

[12] Morse KM. Interorganization Correspondence to R. W. Smith Regarding Asbestos Open Hearth-Moldmen. 1973. p. 1–3.

[13] United States Steel Corporation. Environmental Health Monitoring Manual, 1973. p. 1–211.

[14] Williams PRD, Phelka AD, Paustenbach DJ (2007). A review of historical exposures to asbestos among skilled craftsmen (1940–2006). J Toxicol Environ Health, Part B..

[15] Sheefel T. Letter to S. S. Smith Regarding Asbestos Removal at #1 Powerhouse on May 11, 1995. p. 1–4.

[16] Stockton SD. Interorganization Correspondence to G. R. Hanington Regarding Air Sampling - Update Insulation Removal Project. 1986. p. 1–2.

[17] Industrial Hygiene Dept of U.S. Steel. Sample Results of Phase Contrast Microscopy. USS Chemicals Neville Island, 1986. p. 1–2.

[18] Richter R. Letter to G. Bradley Jr Regarding Asbestos Removal #2 Shot Blaster - Tin Mill Roll Shop. 1989. p. 1–1.

[19] Industrial Hygiene Dept of U.S. Steel. Asbestos Air Samples: Area: #2 Shot Blaster Tin Mill Roll Shop, 1989. p. 1–1

[20] Eagle Industrial Hygiene. Report of Asbestos Sampling and Analysis 880752 U.S. Steel - Fairless Works North Tin Mill. Eagle Industrial Hygiene, 1988. p. 1–5.

[21] Elbel FC. Report to U.S. Steel Corporation Covering the Removal of Asbestos-Containing Materials at the #2 Power House Edgar Thomson Plant of the Mon Valley Works on April 8 and 11, 1985. Industrial Health Foundation Inc.: Pittsburgh, PA, 1985. p. 1–15.

[22] Industrial Hygiene Dept of U.S. Steel. Data Summary Lab Report #880789, 1988. p. 1–1.

[23] Industrial Hygiene Dept of U.S. Steel. Data Summary Lab Report #880791, 1988. p. 1–2.

[24] Industrial Hygiene Dept of U.S. Steel. Data Summary Lab Report #880797, 1988.

[25] Industrial Hygiene Dept of U.S. Steel. Data Summary Lab Report #880804, 1988. p. 1–2.

[26] Industrial Hygiene Dept of U.S. Steel. Data Summary Lab Report #880812, 1988. p. 1–2.

[27] Schneider CM, Patrick G. Letter to James P. Jones regarding asbestos abatement monitoring U.S. Steel - Tin Temper Mill Area BCM Project No. 00-5039-18. 1987. p. 1–10.

[28] Riemer TD. Letter to Margaret Phillips Regarding Air Monitoring Results Power House BCM Project No. 07-5039-62. 1991. p. 1–2.

[29] Riemer T. Air monitoring data report for U.S. Steel at Fairless Hills: Project Number: 07-5039-62. BCM Engineers, 1991. p. 1–1.

[30] Occupational Safety and Health Administration (OSHA). 1994. 29 CFR Parts 1910, 1915, and 1926. Occupational exposure to asbestos: RIN: 1218-AB25. Federal Register. 59. p. 40964–1162.

[31] Budavari S. (ed). The Merck Index. 11th. Merck & Co. Inc., 1989.

[32] Bernstein DM, Hoskins JA (2006). The health effects of chrysotile: Current perspective based upon recent data. Regul Toxicol Pharmacol..

[33] Korchevskiy A, Rasmuson JO, Rasmuson EJ (2019). Empirical model of mesothelioma potency factors for different mineral fibers based on their chemical composition and dimensionality. Inhal Toxicol..

[34] Berman DW, Crump KS (2008). A meta-analysis of asbestos-related cancer risk that addresses fiber size and mineral type. Crit Rev Toxicol..

[35] American Conference of Governmental Industrial Hygienists (ACGIH). Threshold limit values for chemical substances and physical agents in the workroom environment with intended changes for 1978. p. 1–50.

[36] Garabrant DH, Pastula ST (2018). A comparison of asbestos fiber potency and elongate mineral particle (EMP) potency for mesothelioma in humans. Toxicol Appl Pharmacol..

[37] Jaurand MC, Bignon J, Sebastien P, Goni J (1977). Leaching of chrysotile asbestos in human lungs: correlation with in vitro studies using rabbit alveolar macrophages. Environ Res..

[38] Roggli VL, Brody AR (1984). Changes in numbers and dimensions of chrysotile asbestos fibers in lungs of rats following short-term exposure. Exp Lung Res..

[39] Hesterberg TW, Chase G, Axten C, Miller WC, Musselman RP, Kamstrup O (1998). Biopersistence of synthetic vitreous fibers and amosite asbestos in the rat lung following inhalation. Toxicol Appl Pharmacol..

[40] de Klerk NH, Musk AW, Williams V, Filion PR, Whitaker D, Shilkin KB (1996). Comparison of measures of exposure to asbestos in former crocidolite workers from Wittenoom Gorge, W. Australia. Am J Ind Med..

[41] Finkelstein MM, Dufresne A (1999). Inferences on the kinetics of asbestos deposition and clearance among chrysotile miners and millers. Am J Ind Med..

[42] Churg A. Neoplastic asbestos-induced disease. In: Pathology of occupational lung disease. 2nd. Williams & Wilkins, Baltimore, MD, 1998. p. 339–91.

[43] Hodgson JT, Darnton A (2000). The quantitative risks of mesothelioma and lung cancer in relation to asbestos exposure. Ann Occup Hyg..

[44] McDonald AD, McDonald JC (1980). Malignant mesothelioma in North America. Cancer..

[45] Berman DW, Crump KS. Final draft: technical support document for a protocol to assess asbestos-related risk. U.S. Environmental Protection Agency—Office of Solid Waste and Emergency Response: Washington, D.C., 2003. Report no.: EPA#9345.4-06. p. 1–474.

[46] Pierce JS, Ruestow PS, Finley BL (2016). An updated evaluation of reported no-observed adverse effect levels for chrysotile asbestos for lung cancer and mesothelioma. Crit Rev Toxicol..

[47] Berman DW, Crump KS (2008). Update of potency factors for asbestos-related lung cancer and mesothelioma. Crit Rev Toxicol..

[48] Speil S, Leineweber JP (1969). Asbestos minerals in modern technology. Environ Res..

[49] Candela PA, Crummett CD, Earnest DJ, Frank MR, Wylie AG (2007). Low-pressure decomposition of chrysotile as a function of time and temperature. Am Mineral..

[50] Jeyaratnam M, West NG (1994). A study of heat-degraded chrysotile, amosite and crocidolite by X-ray diffraction. Ann Occup Hyg..

[51] Lynch JR, Ayer HE (1968). Measurement of asbestos exposure. J Occup Med..

[52] Kusiorowski R, Zaremba T, Piotrowski J, Adamek J (2012). Thermal decomposition of different types of asbestos. J Therm Anal Calorim..

[53] Bosley JJ. Memorandum to W. H. Mayo regarding use of asbestos in steelmaking and mold manufacturing operations. 1970. p. 1–2.

[54] Janes WC. Interorganization correspondence and asbestos sampling results to R. J. Alberts regarding asbestos survey. 1977. p. 1–7.

[55] Boelter FW, Crawford GN, Podraza DM (2002). Airborne fiber exposure assessment of dry asbestos-containing gaskets and packings found in intact industrial and maritime fittings. AIHA J..

[56] McKinnery WN, Moore RW (1992). Evaluation of airborne asbestos fiber levels during removal and installation of valve gaskets and packing. Am Ind Hyg Assoc J..

[57] Spencer JW. Report of findings: exposure assessment: an evaluation of the actual contribution of airborne asbestos fibers from the removal and installation of gasket and packing from ingersoll-rand compressors and pumps: EPI Project No. 21104. 2001; p. 1–13

[58] Madl AK, Clark K, Paustenbach DJ (2007). Exposure to airborne asbestos during removal and installation of gaskets and packings: a review of published and unpublished studies. J Toxicol Environ Health, Part B..

[59] Mowat F, Bono M, Lee RJ, Tamburello S, Paustenbach D (2005). Occupational exposure to airborne asbestos from phenolic molding material (Bakelite) during sanding, drilling, and related activities. J Occup Environm Hyg..

[60] Occupational Safety and Health Administration (OSHA). 1972. Title 29—Labor: Occupational Safety and Health Administration, Department of Labor: Part 1910—Occcupational Safety and Health Standards: Standard for Exposure to Asbestos Dust. p. 11318–21.

[61] Selikoff IJ (1970). Partnership for Prevention - The Insulation Industry Hygiene Research Program. Ind Med..

[62] U.S. Environmental Protection Agency (EPA). 1990. National Emissions Standards for Hazardous Air Pollutants: 40 CFR. 55. p. 48414–33.

[63] Boelter FW, Spencer JW, Simmons CE (2007). Heavy equipment maintenance exposure assessment: using a time-activity model to estimate surrogate values for replacement of missing data. J Occup Environ Hyg..

[64] Longo WE, Egeland WB, Hatfield RL, Newton LR (2002). Fiber release during the removal of asbestos-containing gaskets: a work practice simulation. Appl Occup Environ Hyg..

[65] Mangold C, Clark K, Madl A, Paustenbach D (2006). An exposure study of bystanders and workers during the installation and removal of asbestos gaskets and packing. J Occup Environ Hyg..

[66] Madl AK, Devlin KD, Perez AL, Hollins DM, Cowan DM, Scott PK (2015). Airborne asbestos exposures associated with gasket and packing replacement: A simulation study of flange and valve repair work and an assessment of exposure variables. Regul Toxicol Pharmacol..

[67] Janes WC. Interorganization correspondence to F. R. Beiser regarding asbestos survey. 1975. p. 1–4.

[68] Janes WC. Interorganization correspondence and asbestos fiber exposures results to P. V. Crooks Regarding Asbestos Survey Waukegan Plant. 1976. p. 1–4.

[69] Morrison PK. Letter to T. G. Wylie regarding asbestos survey. 1977. p. 1–1.

[70] DeMichele FA. Interorganization Correspondence and Analytical Results to P. O. Ecelberger Regarding Plant Asbestos Monitoring Program. 1978. p. 1–4.

[71] McGannon HE (ed). The making, shaping, and treating of steel. U.S. Steel: Pittsburgh, Pennsylvania, 1998.

[72] Deibert RH. Letter to R. Roskov Regarding Irritant Gas and Asbestos Exposure - Stearns Brake Repair. 1985. p. 1–1.

[73] Janes WC. Interorganization Correspondence to R. L. Schneider Regarding Asbestos Survey Maintenance. 1976. p. 1–2.

[74] Asbestos Emissions from Brake Dynamometer Tests. Automobile Engineering Meeting; May 14–18; Detroit, MI. Society of Automotive Engineers, 1973.

[75] Paustenbach DJ, Finley BL, Lu ET, Brorby GP, Sheehan PJ (2004). Environmental and occupational health hazards associated with the presence of asbestos in brake linings and pads (1900 to present): a “state-of-the-art” review. J Toxicol Environ Health, Part B..

[76] Madl AK, Balzer JL, Paustenbach DJ (2009). Airborne asbestos concentrations associated with heavy equipment brake removal. Ann Occup Hyg..

[77] DeMichele FA. Report on area, type of sample and date, employee & Check No., job or location, length of sampling, and asbestos, 1979. p. 1–2.

[78] Paustenbach DJ, Richter RO, Finley BL, Sheehan PJ (2003). An evaluation of the historical exposures of mechanics to asbestos in brake dust. Appl Occup Environ Hyg..

[79] Weil S, Delpire L. Internal Memo to Amy Moll regarding Asbestos Exposure - Brakes and Ambient. 1985. p. 1–2.

[80] Sahmel J, Avens H, Ferracini T, Banducci A, Rickabaugh K. Evaluation of airborne asbestos concentrations associated with the operation and maintenance of brakes and clutches on nonautomated heavy equipment. J Environ Public Health. 2022;2022:1–7. 10.1155/2022/9831883 PMC9054464 35495363

[81] Deibert RH. Interorganization Correspondence to T. D. Puckett regarding asbestos content—brake lining. 1984. p. 1–1.

[82] Bernhart WM. Survey Report: asbestos and organic solvents surveys USS Supply—Chicago. Industrial Hygiene Services, 1984. p. 1–8.

[83] Deibert RH. Sample report for the seamless pipe division: bloom conditioning/hot finishing area at the fairfield works facility. USS Industrial Hygiene, 1985. p. 1–2.

[84] Simulation of automobile brake wear dynamics and estimation of emissions. Passenger Car Meeting; June 6–9, 1983; Dearborn, MI. Society of Automotive Engineers, Inc., 1983.

[85] Hickish DE, Knight KL (1970). Exposure to asbestos during brake maintenance. Ann Occup Hyg..

[86] Brake and clutch emissions generated during vehicle operation. In Proceedings of automobile engineering meeting; May 14–18, 1973; Detroit, MI, 1973.

[87] Luxon S (1970). Technical implementation of the new asbestos regulations. Ann Occup Hyg..

[88] Rohl AN, Langer AM, Klimentidis R, Wolff MS, Selikoff IJ (1977). Asbestos content of dust encountered in brake maintenance and repair. J R Soc Med..

[89] Rowson DM (1978). The Chrysotile content of the wear debris of brake linings. Wear..

[90] Sheehy JW, Cooper TC, O’Brien DM, McGlothlin JD, Froehlich PA. Control of asbestos exposure during brake drum service. U.S. Department of Health and Human Services, Public Health Service, Centers for Disease Control, National Institute for Occupational Safety and Health, 1989. Report no. 89-121. p. 1–70.

[91] Williams RL, Muhlbaier JL (1982). Asbestos brake emissions. Environ Res..

[92] Civic TM. Interorganization Correspondence to Dean Wilson Regarding Asbestos Survey 160”, 100”, 48” and 45” Mills and Wheel and Axle Plant. 1976. p. 1–3.

[93] Balzer JL, Cooper WC. The work environment of insulating workers. Am Ind Hygiene Assoc J. 1968;29:222–7. 10.1080/00028896809342992 5658086

[94] Marr WT (1964). Asbestos exposure during naval vessel overhaul. Am Ind Hyg Assoc J..

[95] Krusell N, Cogley D. Asbestos substitute performance analysis. GCA Corporation: Bedford, Massachusetts, 1982. p. 1–333.

[96] Longo WE, Hatfield RL. The use of asbestos containing gloves (Type: Five Finger) Work Practice Study. Materials Analytical Services, Inc, 2001. p. 1–56.

[97] Cherrie JW, Tindall M, Cowie H (2005). Exposure and risks from wearing asbestos mitts. Part Fibre Toxicol..

[98] Janes WC. Interorganization correspondence to F. R. Smith Jr regarding asbestos dust survey. 1974; p. 1–2.

[99] Bradt RC. Asbestos in refractories—applications and environmental issues. Environ Issues Waste Manag Technol. 2001;119:35–46.

[100] Hollins D, Burns A, Unice K, Paustenbach DJ (2019). An analysis of workplace exposures to asbestos at three steel mills located in the United States (1972-82). Toxicol Ind Health..

[101] Anderson PH, Farino WJ. Analysis of fiber release from certain asbestos products: draft final report - Task 1. GCA Corporation: Bedford (MA), 1982. Report no.: GCA-TR-82-16-G, EPA 68-01-5960. p. 1–141.

[102] Dellisanti F, Minguzzi V, Morandi N (2002). Experimental results from thermal treatment of asbestos containing materials. GeoActa..

[103] Cooper WC, Balzer JL. Evaluation and control of asbestos exposures in the insulating trade. In: Proceedings of the second international conference on the biological effects of asbestos. Dresden, Germany, 1968. p. 151–60.

[104] Shupe K. Industrial hygiene exposure record sample report for 23 sheet products division, 02 Finishing Operations Area for the Fairfield Plant, 1991. p. 1–1.

[105] Mangold CA, Beckett RR, Bessmer DJ. Asbestos exposure and control at puget sound naval station: Bremerton, WA, 1970. Report no.: BUMED-732-SHB: snp. p.1–52.

[106] Hollins DM, Paustenbach DJ, Clark K, Mangold CA (2009). A visual historical review of exposure to asbestos at puget sound naval shipyard (1962-72). J Toxico Environ Health, Part B..

[107] Janes WC. Interorganization Correspondence to C. D. Kelly regarding environmental health survey Masonry Department Edgar Thomson Works. 1974. p. 1–4.

[108] Cooper WC, Miedema J. Asbestosis in the manufacture of insulating materials: IARC Scientific Publications No. 8. In: Bogovski P, Timbrell V, Gilson JC, Wagner JC (eds). Biological Effects of Asbestos. World Health Organization, International Agency for Research on Cancer, Switzerland, 1973. p. 175–8.

[109] Deibert RH. Letter to J. F. Fundin from R. H. Deibert Regarding Primary Mill Crane Cab Insulation. 1979. p. 1–2.

[110] Janes WC. Interorganization Correspondence to T. Stevenson Regarding Material Analysis. 1979. p. 1–1.

[111] McCune TR. Interorganization correspondence to R. E. Frazier regarding asbestos removal from #275 Crane Cab. 1981. p. 1–1.

[112] Churg A, Warnock ML (1977). Correlation of quantitative asbestos body counts and occupation in urban patients. Arch Pathol Lab Med..

[113] Occupational Safety and Health Administration (OSHA). 1995. 29 CFR Parts 1910, 1915, and 1926 RIN 1218-AB25 Occupational Exposure to Asbestos; Corrections. Federal Register. 60. p. 33974–4002.

[114] Anderson CD. Interorganization Correspondence to Ron Metcalfe Regarding Asbestos Monitoring - Caster Shipping Cranes 1–4. 1993. p. 1–1.

[115] Industrial Hygiene Dept of U.S. Steel. Airborne Fiber Concentration BOP Shop, Edgar Thomson Plant, Mon Valley Works USX Corporation, 1993. p. 1–1.

[116] CONSAD Research Corporation. Final Report: Economic and Technological Profile Related to OSHA’s Revised Permanent Asbestos Standard for the Construction Industry and Asbestos Removal and Routine Maintenance Projects in General Industry: Contract Number: J-9-F-4-0024. In. Pittsburgh, Pennsylvania, 1985. p. 1–16.

[117] Morse KM. Interorganization Correspondence to Peter M. Serokis Regarding Asbestos Survey Insulation Work at Neville Island Plant. 1972. p. 1–2.

[118] Industrial Hygiene Dept of U.S. Steel. Asbestos Survey and Inspection Report for Coke and Chemicals Division, #3 Battery Department, 1990. p. 1–1.

[119] Quealy JF. Procedures for removing insulation materials containing asbestos. 1980. p. 1–3.

[120] McGannon HE. (ed). The making, shaping, and treating of steel. 7th ed. United States Steel: Pittsburgh, Pennsylvania, 1957.

[121] Inglis B. Aileen L Wedvik as Personal Representative of the Estate of Lawrence L. Wedvik, Plaintiff vs. C. H. Murphy/Clark-Ullman Inc., et al, Defendants: Videotaped Deposition Transcript of: Bruce Inglis, CPA. In: Desiree Guzman. Seattle, WA: Superior Court of Washington for Pierce County, 2015.

[122] Janes WC. Interorganization Correspondence to J. P. Jones Regarding Asbestos Survey. 1976. p. 1–3.

[123] Foseco Inc. In Re: All Asbestos Litigation Filed by the Simmons Firm. In: In the Circuit Court of the Third Judical Circuit Madison County, Illinois. p. 1–126.

[124] Baumann WH. Interorganization correspondence to H. N. Hubbard regarding airborne asbestos exposures #2 & #3 hot top houses - Gary Works. 1973. p. 1–6.

[125] Masaitis JB. Interorganization correspondence to K. M. Morse regarding asbestos open Hearth Homestead Works. 1973. p. 1–1.

[126] Mishra B, Mustoe G (2014). Temperatures reached in asbestos-containing refractory materials during ingot casting of steel. Int J Eng Res Ind Appl..

[127] Morse KM. Letter to J. J. Grimes regarding preliminary asbestos survey at Worchester Works.1972:1–6.

[128] Louhenkilpi S. Continuous casting of steel. In: Seetharaman S (ed) Treatise on process metallurgy. Elsevier, Boston, 2014. p. 373–434.

[129] Stromness NR. Letter and Asbestos Exposures Results to W. J. Kelly and J. F. Boal Regarding Asbestos Exposures - 14 Battery Rebuild. 1980. p. 1–3.

[130] United States Steel. Locations: U.S. Steel’s Footprint. 2022. https://www.ussteel.com/about-us/locations.

[131] United States Steel. Mon Valley Works Edgar Thomson Plant Operations and Environmental Report. 2020, p. 1–35.

[132] Keter Consultants Inc. Asbestos Negative Exposure Assessment at 2 Boiler House for U.S. Steel - Gary Works. Keter Consultants Inc.: Willow Springs, Illinois, 2001. p. 1–31.

[133] Donovan EP, Donovan BL, Shahmel J, Scott PK, Paustenbach DJ (2011). Evaluation of bystander exposures to asbestos in occupational settings: a review of the literature and application of a simple eddy diffusion model. Crit Rev Toxicol..

[134] Donovan EP, Donovan BL, McKinley MA, Cowan DM, Paustenbach DJ (2012). Evaluation of take home (para-occupational) exposure to asbestos and disease: a review of the literature. Crit Rev Toxicol..

[135] Janes WC. Interorganization Correspondence and Laboratory Results to C. D. Kelly Regarding Environmental Health Survey Masonry Department Edgar Thomson Works. 1974. p. 1–8.

[136] Deibert RH. Letter to Frank Burton Jr Regarding Asbestos Survey - No. 2 Coke Battery. 1981. p. 1–2.

[137] Richter R. Letter to S. S. Smith Regarding Asbestos Contamination East Side Electrical Substations. 1995. p. 1–2.

[138] Birkner JS, Colton CE. Respiratory protective equipment. In: Rose VE, Cohrssen B (eds). Patty’s Industrial Hygiene. Sixth. vol. 2. John Wiley & Sons, Hoboken, New Jersey, 2011. p. 1169–233.

[139] Janes WC. Interorganization correspondence and asbestos exposures results to D. W. Brochstein Regarding Asbestos Survey Chicago Heights Plant. 1977. p. 1–4.

[140] Occupational Safety and Health Administration (OSHA). Assigned Protection Factors for the Revised Respiratory Protection Standard. In: Occupational Safety and Health Administration, U.S. Department of Labor, 2009. p. 1–51.

[141] Kucata B. Sample Report for Duquesne Plant Location: Carpenter Shop Department at the National-Duquesne Facility. USS Environmental Health, 1981. p. 1–1.

[142] Quealy JF. Interorganization correspondence to R. H. Jebo Regarding Environmental Health Survey Alameda Plant. 1981. p.1–10.

[143] Pokrywka F. Sample Report for #275 Crane Cab Location: 100” Mill A-Yard Department at the Homestead Works Facility. USS Environmental Health, 1981. p. 1–1.

[144] Quealy JF. Interorganization Correspondence and Samples Results to George Sorna Regarding Sample Analyses Data.1981; p. 1–2.

[145] Paustenbach DJ. The engineer’s responsibility in occupational prevention disease. In: Cralley LJ, Cralley LV, Mutchler JE (eds). Industrial hygiene aspects of plant operations. 2. Macmillan Publishing Company, New York, NY, 1984. p. 5–24.

[146] Paustenbach DJ, Cyrs WD, Cohrssen B (2021). The history and biological basis of occupational exposure limits for chemical agents. Patty’s industrial hygiene..

[147] Paustenbach DJ (ed). The risk assessment of environmental and human health hazards: a textbook of case studies. Wiley: New York, NY, 1989.

[148] Rodricks JV, Brett SN, Wrenn GC (1987). Risk decisions in federal regulatory agencies. Regul Toxicol Pharmacol..

[149] Occupational Safety and Health Administration (OSHA). 29 CFR Parts 1910 and 1926: Occupational Exposure to Asbestos, Tremolite, Anthophyllite, and Actinolite. Federal Register. 1986. p. 51

[150] Martonik JF, Nash E, Grossman E (2001). The history of OSHA’s asbestos rulemakings and some distinctive approaches that they introduced for regulating occupational exposure to toxic substances. Am Ind Hyg Assoc J..

[151] Keter Consultants Inc. Occupation Water Treatment Plant Operator and Airborne Asbestos Sample Results, 2001. p. 1–3.

[152] National Institute for Occupational Safety & Health (NIOSH). Asbestos and other fibers by PCM. In: NIOSH manual of analytical methods. 5th Edition, 2019. p. 1–40.

[153] Keter Consultants Inc. Airborne Asbestos Monitoring for U.S. Steel—Gary Works, 1997. p. 1–3.

[154] Little DF. Sample results regarding asbestos fiber analysis by transmission electron microscopy (TEM), selected area electron diffraction (SAED), and energy dispersive X-ray microanalysis (EDX)—performed by EPA leval II method to Keter Consultants, Inc., 1997. p. 1–1.

[155] Keter Consultants Inc. Airborne asbestos monitoring for U.S. Steel - Gary Works, Utilitymen L. Gordon, K. Price, and A. Delmuro, 1997. p. 1–1.

[156] Keter Consultants Inc. Asbestos negative exposure assessment at turbo blower complex for U.S. Steel - Gary Works. Keter Consultants, Inc.: Willow Springs, Illinois, 1997.

[157] Keter Consultants Inc. Airborne asbestos concentrations sample results regarding turbo blower complex at U.S. Steel - Gary Works, 1997. p. 1–4.

[158] Cralley LV, Cralley LJ. (eds). In-plant practices for job related health hazards control: production processes. Wiley-Interscience, 1989.

[159] Roggli VL, Sporn TA, Case BW, Butnor KJ (2009). Comments on asbestos fibre concentrations in the lungs of brake workers: another look. Ann Occup Hyg..

[160] Mazurek JM, Syamlal G, Wood JM, Hendricks SA, Weston A (2017). Morbidity and mortality weekly report: malignant mesothelioma mortality—United States, 1999–2015. Morb Mortal Wkly Rep..

[161] Lloyd JW, Ciocco A (1969). Long-term mortality study of steelworkers: I. Methodology. J Occup Med..

[162] Redmond CK, Smith EM, Lloyd JW, Rush HW (1969). Long-term mortality study of steelworkers: III Follow-up. J Occup Med..

[163] Robinson H (1969). Long-term mortality study of steelworkers: II. Mortality by level of income in Whites and Non-Whites. J Occup Med..

[164] Lloyd JW, Lundin FE, Redmond CK, Geiser PB (1970). Long-term mortality study of steelworkers: IV. Mortality by work area. J Occup Med..

[165] Lloyd JW (1971). Long-term mortality study of steelworkers V. Respiratory cancer in coke plant workers. J Occup Med..

[166] Redmond CK, Ciocco A, Lloyd JW, Rush HW (1972). Long-term mortality study of steelworkers: VI—Mortality from malignant neoplasms among coke oven workers. J Occup Med..

[167] Lerer TJ, Redmond CK, Breslin PP, Salvin L, Rush HW (1974). Long-term mortality study of steelworkers: VII. Mortality patterns among crane operators. J Occup Med..

[168] Mazumdar S, Lerer TJ, Redmond CK (1975). Long-term mortality study of steelworkers. IX. Mortality patterns among sheet and tin mill workers. J Occup Med..

[169] Redmond CK, Gustin J, Kamon E (1975). Long-term mortality experience of steelworkers. VIII Mortality patterns of open hearth steelworkers (a preliminary report). J Occup Med..

[170] Rockette HE, Redmond CK (1976). Long-term mortality study of steelworkers: X. Mortality patterns among masons. J Occup Med..

[171] Redmond CK, Wieand HS, Rockette HE, Sass R, Weinberg G. Long-term mortality experience of steelworkers. U.S. Department of Health and Human Services, Public Health Service, Centers for Disease Control, National Institute for Occupational Safety and Health, Division of Surveillance, Hazard Evaluations and Field Studies: Cincinnati, Ohio, 1981. p. 1–132.

[172] Mazumdar S, Redmond C, Sollecito W, Sussman N (1975). An epidemiological study of exposure to coal tar pitch volatiles among coke oven workers. J Air Pollut Control Assoc..

[173] Redmond CK (1983). Cancer mortality among coke oven workers. Environ Health Perspect..

[174] Costantino JP, Redmond CK, Bearden A (1995). Occupationally related cancer risk among coke oven workers: 30 years of follow-up. J Occup Environ Hyg..

[175] Walker AM, Loughlin JE, Friedlander ER, Rothman KJ, Dreyer NA (1983). Projections of asbestos-related disease 1980-2009. J Occup Med..

[176] McDonald JC (1985). Health implications of environmental exposure to asbestos. Environ Health Perspect..

[177] Ilgren EB, Wagner JC (1991). Background incidence of mesothelioma: animal and human evidence. Regul Toxicol Pharmacol..

[178] McDonald JC, McDonald AD. Mesothelioma: is there a background? In: Jaurand M-C, Bignon J (eds). The mesothelial cell and mesothelioma. 78. Marcel Dekker, New York, NY, 1994. p. 37–44.

[179] Huncharek M (2002). Non-asbestos related diffuse malignant mesothelioma. Tumori..

[180] Price B, Ware A (2004). Mesothelioma trends in the united states: an update based on surveillance, epidemiology, and end results program data for 1973 through 2003. Am J Epidemiol..

[181] Teta MJ, Mink PJ, Lau E, Sceurman BK, Foster ED (2008). US mesothelioma patterns 1973–2002: indicators of change and insights into background rates. Eur J Cancer Prevent..

[182] Moolgavkar SH, Meza R, Turim J (2009). Pleural and peritoneal mesotheliomas in SEER: age effects and temporal trends, 1973–2005. Cancer Causes Control..

[183] Price B, Ware A (2009). Time trend of mesothelioma incidence in the United States and projection of future cases: an update based on SEER data for 1973 through 2005. Crit Rev Toxicol..

[184] Price B (2022). Projection of future numbers of mesothelioma cases in the US and the increasing prevalence of background cases: an update based on SEER data for 1975 through 2018. Crit Rev Toxicol..

[185] Cavazza A, Travis LB, Travis WD, Wolfe JT, Foo ML, Gillespie DJ (1996). Post-irradiation malignant mesothelioma. Cancer..

[186] Travis LB, Fosså SD, Schonfeld SJ, McMaster ML, Lynch CF, Storm H (2005). Second cancers among 40 576 testicular cancer patients: focus on long-term survivors. J Natl Cancer Inst..

[187] Tward JD, Wendland MMM, Shrieve DC, Szabo A, Gaffney DK (2006). The risk of secondary malignancies over 30 years after the treatment of non-Hodgkin lymphoma. Cancer..

[188] Hodgson DC, Gilbert ES, Dores GM, Schonfeld SJ, Lynch CF, Storm H (2007). Long-term solid cancer risk among 5-year survivors of hodgkin’s lymphoma. J Clin Oncol..

[189] Teta MJ, Lau E, Sceurman BK, Wagner ME (2007). Therapeutic radiation for lymphoma: risk of malignant mesothelioma. Cancer..

[190] Goodman JE, Nascarella MA, Valberg PA (2009). Ionizing radiation: a risk factor for mesothelioma. Cancer Causes Control..

[191] Attanoos RL, Churg A, Galateau-Salle F, Gibbs AR, Roggli VL (2018). Malignant mesothelioma and its non-asbestos causes. Arch Pathol Lab Med..

[192] Hillerdal G, Baris Y (1983). Radiological study of pleural changes in relation to mesothelioma in Turkey. Thorax..

[193] DeMichele FA. Interorganization Correspondence to P. O. Ecelberger Regarding Asbestoe Monitoring. 1979. p. 1–2.

[194] DeMichele FA. Interorganization Correspondence and Analytical Results to P. O. Ecelberger Regarding Plant Asbestos Monitoring Program. 1979. p. 1–5.

[195] U.S. Steel IH Report. Report on 45” Rolling Mill Inspection for Asbestos and Inspection for Asbestos in Building #1, 1985. p. 1–3.

[196] Keter Consultants Inc. Airborne Asbestos Concentration 2 Boiler House - Boiler Utility Table 1, 2001. p. 1–1.

[197] Mulhausen JR, Milz S, Hewett P, Damiano J. Quantitative exposure data: interpretation, decision making, and statistical tools. In: Jahn SD, Bullock WH, Ignacio J (eds). A strategy for assessing and managing occupational exposures. American Industrial Hygiene Association (AIHA), 2015. p. 125–42.

