# Supplementary material for: Occupational exposure to asbestos in the steel industry (1972–2006)
Source: J Expo Sci Environ Epidemiol. 2023 Jul 26;34(3):465–95. doi: 10.1038/s41370-023-00576-4 (PMC11222148; doi:10.1038/s41370-023-00576-4)
Supplement: Supplementary file 1 — Supplementary information [file 41370_2023_576_MOESM1_ESM.docx]

| **Table A.** List of the 21 job classifications and the job titles, as they appear in U. S. Steel industrial hygiene reports, within each classification grouping. | | | |
| --- | --- | --- | --- |
| **Blast Furnace Operators:** | **Boiler House Operators:** | **Coke Operators:** | **Finishing Operator:** |
| Helper | Operator | Door Machine Operator | Annealing Pot Tender |
| Keeper | Engineer | Helper | Blocker |
| **Foundry Operator:** | Fan Tender | **Craftsman:** | Cinderman |
| Core Setter | Fireman | Ironworker | Coil Bander |
| Oven Tender | Helper | Pipefitter | Feeder |
| **Melt Shop operator:** | Pump Tender | Boilermaker | Furnace Operator |
| 1^st^ Helper | **Oversight:** | Oven Patcher | Heater |
| Charging Machine Operator | Coordinator | **Insulator:** | Helper |
| **Bricklayer:** | Group Leader | Pipe Coverer | Line Operator |
| Bricklayer | Foreman | Insulator | Mill Hot Shear Stamper |
| Bricklayer’s Helper | Superintendent | Laborer | Operator |
| **Electrician:** | Tool Room Attendant | **Foundrymen:** | Quencher |
| Electrician | Team Leader | Sand Reclaimer | Shot Blaster |
| Wireman | Manager | Shakeout Man | Tinner |
| **Crane/Forklift operator:** | Process Observer | Caster | **Finishing process support:** |
| Crane Operator | **Mechanical Maintenance:** | Moldman (Foundry) | Hooker |
| Forklift Operator | Mechanical Repairman | **Research Janitor:** | Layerout |
| **Brake repairman:** | Tool Room Attendant | Janitor | Physical Tester |
| Automotive Mechanic | Laborer | **Research tester:** | Roll Hand Tester |
| **Moldmen:** | Repairman | Chemical Technician | Shipper |
| Moldman | Millwright | Physical Tester | Shipper Helper |
| Hot Top Liner | Mechanic | Test Preparer | Stocker |
| **Boiler cleaner:** | Utilityman | **Motor Inspector:** | Weighter |
| Boiler Cleaner |  | Motor Inspector | Wiper |
|  |  |  | Wrapper |

| **Table B.** Approximate sample size requirements to be 95% confident the true 95^th^ percentile is less than the OEL | | | | | |
| --- | --- | --- | --- | --- | --- |
| **Ratio: true 95th/OEL** | **Low Variability (GSD = 1.5)** | **GSD = 2** | **Moderate Variability (GSD = 2.5)** | **GSD = 3** | **High Variability (GSD = 3.5)** |
| 0.75 | 53 | 138 | 231 | 326 | 418 |
| 0.5 | 13 | 30 | 47 | 65 | 82 |
| 0.25 | 6 | 10 | 16 | 20 | 25 |
| 0.1 | 4 | 6 | 8 | 10 | 12 |

This Figure has been recreated from Table 8.1 in the AIHI’s *A Strategy for Assessing and Managing Occupational Exposures Fourth Edition*. Note: The sample sizes were calculated using Equation 5 in Chap/Appendix X Sampling Strategy Design

| **Table C1.** Geometric mean and standard deviation of all personal air sampling (representative workday samples and task samples) for airborne fiber concentrations (1972-2006) | | | | | | | |
| --- | --- | --- | --- | --- | --- | --- | --- |
|  |  |  | | **Asbestos Fiber Concentration by PCM (fibers/cc)** | | | |
| **Sample Type** | **Time Period** | | **n** | **GM** | **GSD** | **95th Percentile** | **PEL** |
| Representative Workday | 1972-1975 | | 45 | 0.33 | 0.03 | 4.50 | 5 |
|  | 1976-1985 | | 105 | 0.02 | *7.88 | 1.11 | 2 |
|  | 1986-1993 | | 152 | 0.01 | 3.41 | 0.10 | 0.2 |
|  | 1994-2006 | | 35 | 0.02 | 2.91 | 0.09 | 0.1 |
| Task | 1972-1975 | | 22 | 1.18 | *4.49 | 13.70 | 5 |
|  | 1976-1985 | | 98 | 0.05 | *11.50 | 1.12 | 2 |
|  | 1986-1993 | | 6 | 0.01 | 1.59 | -- | 0.2 |
|  | 1994-2006 | | 32 | 0.03 | 2.23 | 0.07 | 0.1 |
| **Total** |  | | **495** |  |  |  |  |

Empty (--) values were not calculated because the number of samples were deemed too low. Standard deviations were calculated for all samples with an n of 5 or greater. 95th percentiles were calculated for n of 20 or greater. *GSD greater than 3.5 suggests a high level of variability.

| **Table C2.** Geometric mean and geometric standard deviation of all task samples for airborne fiber concentrations at 16 different U. S. Steel Facilities (1972-2006) | | | | | | | | |
| --- | --- | --- | --- | --- | --- | --- | --- | --- |
|  |  |  | **Asbestos Fiber Concentration by PCM (fibers/cc)** | | | | | |
| **Facility Name** | **Time Period** | | **n** | **GM** | **GSD** | **95th Percentile** | **Max** | **PEL** |
| Edgar-Thomson Plant | 1972-1975 | | 1 | 0.35 | -- | -- | 0.35 | 5 |
|  | 1976-1985 | | 7 | 0.01 | *11.18 | -- | 0.25 | 2 |
| Fairfield Works | 1976-1985 | | 16 | 0.07 | *7.62 | -- | 1.01 | 2 |
| Fairless Works | 1976-1985 | | 35 | 0.05 | *18.89 | 7.79 | 17.46 | 2 |
| Gary Works | 1972-1975 | | 16 | 1.56 | *4.99 | -- | 23.80 | 5 |
|  | 1976-1985 | | 4 | 0.35 | -- | -- | 0.94 | 2 |
|  | 1986-1993 | | 6 | 0.01 | 1.59 | -- | 0.01 | 0.2 |
|  | 1994-2006 | | 32 | 0.03 | 2.23 | 0.07 | 0.07 | 0.1 |
| Geneva Works | 1976-1985 | | 4 | 0.12 | -- | -- | 0.38 | 2 |
| Homestead Works | 1972-1975 | | 5 | 0.61 | 2.62 | -- | 1.79 | 5 |
|  | 1976-1985 | | 10 | 0.02 | *5.98 | -- | 0.12 | 2 |
| Irvin Works | 1976-1985 | | 1 | 0.001 | -- | -- | 0.001 | 2 |
| Mon Valley Works | 1976-1985 | | 2 | 0.33 | -- | -- | 0.58 | 5 |
| National Duquesne Works | 1976-1985 | | 2 | 0.04 | -- | -- | 0.04 | 2 |
| Neville Island Plant | 1976-1985 | | 16 | 0.06 | *10.52 | -- | 0.88 | 2 |
| Pittsburg Works | 1976-1985 | | 1 | 0.05 | -- | -- | 0.05 | 2 |
| **Total** |  | | **158** |  |  |  |  |  |

Empty (--) values were not calculated because the number of samples were deemed too low. Standard deviations were calculated for all samples with an n of 5 or greater. 95th percentiles were calculated for n of 20 or greater. *GSD greater than 3.5 suggests a high level of variability. If the maximum value is low, a high GSD is due to non-detects or extremely low values in the data group.

| **Table C3.** Geometric mean and geometric standard deviation of all representative workday samples for airborne fiber concentrations at 16 different U. S. Steel Facilities (1972-2006) | | | | | | | | | |
| --- | --- | --- | --- | --- | --- | --- | --- | --- | --- |
|  |  |  | | **Asbestos Fiber Concentration by PCM (fibers/cc)** | | | | | |
| **Facility Name** | **Time Period** | | **n** | | **GM** | **GSD** | **95th Percentile** | **Max** | **PEL** |
| Clairton Works | 1976-1985 | | 8 | | 0.05 | 1.59 | -- | 0.09 | 2 |
|  | 1986-1993 | | 48 | | 0.02 | 3.20 | 0.13 | 0.16 | 0.2 |
| Eastern Steel Division | 1976-1985 | | 2 | | 0.20 | -- | -- | 0.22 | 2 |
| Edgar-Thomson Plant | 1972-1975 | | 1 | | 0.98 | -- | -- | 0.98 | 5 |
|  | 1976-1985 | | 10 | | 0.003 | *4.23 | -- | 0.05 | 2 |
|  | 1986-1993 | | 58 | | 0.01 | *3.62 | 0.13 | 0.13 | 0.2 |
| Fairfield Works | 1976-1985 | | 6 | | 0.12 | 2.24 | -- | 0.36 | 2 |
|  | 1986-1993 | | 7 | | 0.003 | *4.16 | -- | 0.04 | 0.2 |
| Fairless Works | 1972-1975 | | 8 | | 3.41 | 2.11 | -- | 10.00 | 5 |
|  | 1976-1985 | | 27 | | 0.04 | *15.52 | 1.28 | 1.36 | 2 |
|  | 1986-1993 | | 15 | | 0.004 | 2.38 | -- | 0.03 | 0.2 |
| Gary Works | 1972-1975 | | 4 | | 0.67 | -- | -- | 0.80 | 5 |
|  | 1986-1993 | | 16 | | 0.01 | 1.96 | -- | 0.02 | 0.2 |
|  | 1994-2006 | | 35 | | 0.02 | 2.91 | 0.09 | 0.17 | 0.1 |
| Homestead Works | 1972-1975 | | 6 | | 0.11 | *5.58 | -- | 2.92 | 5 |
|  | 1976-1985 | | 27 | | 0.01 | *6.42 | 0.17 | 0.26 | 2 |
| Johnstown Works | 1972-1975 | | 11 | | 0.08 | 2.60 | -- | 0.87 | 5 |
| Mon Valley Works | 1976-1985 | | 2 | | 0.01 | -- | -- | 0.01 | 5 |
|  | 1986-1993 | | 8 | | 0.01 | 1.50 | -- | 0.01 | 2 |
| National Duquesne Works | 1976-1985 | | 9 | | 0.01 | 1.12 | -- | 0.07 | 2 |
| Neville Island Plant | 1972-1975 | | 15 | | 0.32 | 2.17 | -- | 1.10 | 5 |
|  | 1976-1985 | | 2 | | 0.004 | *8.32 | -- | 0.02 | 2 |
| New Haven Works | 1976-1985 | | 2 | | 0.001 | -- | -- | 0.001 | 2 |
| Pittsburg Works | 1976-1985 | | 2 | | 0.01 | -- | -- | 0.01 | 2 |
| Waukegan Works | 1976-1985 | | 8 | | 0.03 | 1.28 | -- | 0.04 | 2 |
| **Total** |  | | **337** | |  |  |  |  |  |

Empty (--) values were not calculated because the number of samples were deemed too low. Standard deviations were calculated for all samples with an n of 5 or greater. 95th percentiles were calculated for n of 20 or greater. *GSD greater than 3.5 suggests a high level of variability. If the maximum value is low, a high GSD is due to non-detects or extremely low values in the data group.

| **Table C4.** Geometric mean and geometric standard deviation of all task samples for airborne fiber concentrations at nine different U. S. Steel Departments (1972-2006) | | | | | | | | |
| --- | --- | --- | --- | --- | --- | --- | --- | --- |
|  |  |  | | **Asbestos Fiber Concentration by PCM (fibers/cc)** | | | | |
| **Department** | **Time Period** | | **n** | **GM** | **GSD** | **95th Percentile** | **Max** | **PEL** |
| Blast Furnace | 1972-1975 | | 3 | 1.05 | -- | -- | 1.79 | 5 |
| Boiler House | 1976-1985 | | 5 | 0.01 | *4.27 | -- | 0.04 | 2 |
|  | 1986-1993 | | 2 | 0.005 | -- | -- | 0.01 | 0.2 |
|  | 1994-2006 | | 30 | 0.03 | 1.74 | 0.07 | 0.07 | 0.1 |
| Central Maintenance | 1972-1975 | | 1 | 0.35 | -- | -- | 0.35 | 5 |
|  | 1976-1985 | | 47 | 0.06 | *12.82 | 1.22 | 17.46 | 2 |
|  | 1986-1993 | | 4 | 0.01 | 1.42 | -- | 0.01 | 0.2 |
|  | 1994-2006 | | 1 | 0.03 | -- | -- | 0.03 | 0.1 |
| Coke | 1976-1985 | | 24 | 0.05 | *12.08 | 1.01 | 2.30 | 2 |
| Finishing | 1972-1975 | | 1 | 0.21 | -- | -- | 0.21 | 5 |
|  | 1976-1985 | | 8 | 0.08 | *8.03 | -- | 0.86 | 2 |
| Masonry | 1972-1975 | | 1 | 0.35 | -- | -- | 0.35 | 5 |
|  | 1976-1985 | | 5 | 0.03 | *6.94 | -- | 0.12 | 2 |
| Melt Shop | 1972-1975 | | 16 | 1.56 | *4.99 | -- | 23.80 | 5 |
|  | 1976-1985 | | 7 | 0.02 | *16.66 | -- | 1.04 | 2 |
|  | 1994-2006 | | 1 | 0.001 | -- | -- | 0.001 | 0.1 |
| Research Labs | 1976-1985 | | 2 | 0.50 | -- | -- | 0.51 | 2 |
| **Total** |  | | **158** |  |  |  |  |  |

Empty (--) values were not calculated because the number of samples were deemed too low. Standard deviations were calculated for all samples with an n of 5 or greater. 95th percentiles were calculated for n of 20 or greater. *GSD greater than 3.5 suggests a high level of variability. If the maximum value is low, a high GSD is due to non-detects or extremely low values in the data group.

| **Table C5.** Geometric mean and geometric standard deviation of all representative workday samples for airborne fiber concentrations at nine different U. S. Steel Departments (1972-2006) | | | | | | | | |
| --- | --- | --- | --- | --- | --- | --- | --- | --- |
|  |  |  | | **Asbestos Fiber Concentration by PCM (fibers/cc)** | | | | |
| **Department** | **Time Period** | | **n** | **GM** | **GSD** | **95th Percentile** | **Max** | **PEL** |
| Blast Furnace | 1972-1975 | | 1 | 2.92 | -- | -- | 2.92 | 5 |
|  | 1976-1985 | | 6 | 0.10 | 2.29 | -- | 0.23 | 2 |
| Boiler House | 1976-1985 | | 12 | 0.02 | *6.00 | -- | 0.36 | 2 |
|  | 1986-1993 | | 37 | 0.01 | 2.84 | 0.08 | 0.10 | 0.2 |
|  | 1994-2006 | | 31 | 0.01 | 2.91 | 0.09 | 0.17 | 0.1 |
| Central Maintenance | 1972-1975 | | 15 | 0.32 | 2.17 | -- | 1.10 | 5 |
|  | 1976-1985 | | 6 | 0.03 | 3.16 | -- | 0.09 | 2 |
|  | 1986-1993 | | 10 | 0.01 | 2.14 | -- | 0.03 | 0.2 |
|  | 1994-2006 | | 1 | 0.01 | -- | -- | 0.01 | 0.1 |
| Coke | 1976-1985 | | 6 | 0.001 | *9.34 | -- | 0.17 | 2 |
|  | 1986-1993 | | 46 | 0.02 | 3.28 | 0.13 | 0.16 | 0.2 |
| Finishing | 1972-1975 | | 2 | 0.36 | -- | -- | 0.87 | 5 |
|  | 1976-1985 | | 51 | 0.02 | *7.79 | 1.20 | 1.28 | 2 |
|  | 1986-1993 | | 42 | 0.01 | *4.19 | 0.13 | 0.13 | 0.2 |
| Foundry | 1972-1975 | | 10 | 0.06 | 1.75 | -- | 0.13 | 5 |
| Masonry | 1972-1975 | | 5 | 0.08 | *4.19 | -- | 0.98 | 5 |
|  | 1976-1985 | | 6 | 0.01 | *15.61 | -- | 0.22 | 2 |
| Melt Shop | 1972-1975 | | 12 | 1.98 | 2.73 | -- | 10.00 | 5 |
|  | 1976-1985 | | 12 | 0.03 | *12.28 | -- | 1.36 | 2 |
|  | 1986-1993 | | 5 | 0.004 | 2.55 | -- | 0.02 | 0.2 |
|  | 1994-2006 | | 3 | 0.05 | -- | -- | 0.06 | 0.1 |
| Research Labs | 1976-1985 | | 6 | 0.002 | 3.40 | -- | 0.02 | 2 |
|  | 1986-1993 | | 12 | 0.01 | 1.74 | -- | 0.01 | 0.2 |
| **Total** |  | | **337** |  |  |  |  |  |

Empty (--) values were not calculated because the number of samples were deemed too low. Standard deviations were calculated for all samples with an n of 5 or greater. 95th percentiles were calculated for n of 20 or greater. *GSD greater than 3.5 suggests a high level of variability. If the maximum value is low, a high GSD is due to non-detects or extremely low values in the data group.

| **Table C6.** Geometric means and standard deviations for task sample airborne fiber concentrations for Blast Furnace (BF) operators in that department (1972-1985) | | | | | | | | | | |  |
| --- | --- | --- | --- | --- | --- | --- | --- | --- | --- | --- | --- |
|  |  |  | |  | | **Asbestos Fiber Concentration by PCM (fibers/cc)** | | | | | |
| **Job Title** | **Time Period** | | **n** | | **GM** | | **GSD** | **95th percentile** | **Max** | **PEL** | |
| BF Operator | 1972-1975 | | 3 | | 1.05 | | -- | -- | 1.79 | 5 | |

Empty (--) values were not calculated because the number of samples were deemed too low. Standard deviations were calculated for all samples with an n of 5 or greater. 95th percentiles were calculated for n of 20 or greater. *GSD greater than 3.5 suggests a high level of variability. If the maximum value is low, a high GSD is due to non-detects or extremely low values in the data group.

| **Table C7.** Geometric means and standard deviations for representative workday sample airborne fiber concentrations for Blast Furnace (BF) operators in that department (1972-1985) | | | | | | | | | | |
| --- | --- | --- | --- | --- | --- | --- | --- | --- | --- | --- |
|  |  |  | |  | | **Asbestos Fiber Concentration by PCM (fibers/cc)** | | | | |
| **Job Title** | **Time Period** | | **n** | | **GM** | | **GSD** | **95th percentile** | **Max** | **PEL** |
| BF Operator | **overall** | | 7 | | 0.16 | | 1.49 | -- | 2.92 |  |
|  | 1972-1975 | | 1 | | 2.92 | | -- | -- | 2.92 | 5 |
|  | 1976-1985 | | 6 | | 0.10 | | 2.29 | -- | 0.23 | 2 |

Empty (--) values were not calculated because the number of samples were deemed too low. Standard deviations were calculated for all samples with an n of 5 or greater. 95th percentiles were calculated for n of 20 or greater. *GSD greater than 3.5 suggests a high level of variability. If the maximum value is low, a high GSD is due to non-detects or extremely low values in the data group.

| **Table C8.** Geometric means and standard deviations for task sample airborne fiber concentrations in the Boiler House department grouped by job category (1972-2006) | | | | | | | | |
| --- | --- | --- | --- | --- | --- | --- | --- | --- |
|  | | | **Asbestos Fiber Concentration by PCM (fibers/cc)** | | | | | |
| **Job Title** | **Time Period** | **n** | | **GM** | **GSD** | **95^th^ Percentile** | **Max** | **PEL** |
| BH Operator | 1994-2006 | 8 | | 0.02 | 2.21 | -- | 0.06 | 0.1 |
|  |  |  | |  |  |  |  |  |
| Mechanical Maintenance | **overall** | 24 | | 0.02 | 2.61 | 0.07 | 0.07 |  |
|  | 1976-1985 | 2 | | 0.004 | -- | -- | 0.01 | 2 |
|  | 1986-1993 | 2 | | 0.005 | -- | -- | 0.01 | 0.2 |
|  | 1994-2006 | 20 | | 0.03 | 1.52 | 0.07 | 0.07 | 0.1 |
|  |  |  | |  |  |  |  |  |
| Boiler Cleaner | 1976-1985 | 3 | | 0.03 | -- | -- | 0.04 | 2 |
|  |  |  | |  |  |  |  |  |
| Insulator | 1994-2006 | 2 | | 0.03 | -- | -- | 0.04 | 0.1 |

Empty (--) values were not calculated because the number of samples were deemed too low. Standard deviations were calculated for all samples with an n of 5 or greater. 95th percentiles were calculated for n of 20 or greater. *GSD greater than 3.5 suggests a high level of variability. If the maximum value is low, a high GSD is due to non-detects or extremely low values in the data group.

| **Table C9.** Geometric means and standard deviations for representative workday sample airborne fiber concentrations in the Boiler House department grouped by job category (1972-2006) | | | | | | | | | | |
| --- | --- | --- | --- | --- | --- | --- | --- | --- | --- | --- |
|  |  |  | |  | **Asbestos Fiber Concentration by PCM (fibers/cc)** | | | | | |
| **Job Title** | **Time Period** | | **n** | | | **GM** | **GSD** | **95^th^ Percentile** | **Max** | **PEL** |
| BH Operator | **overall** | | 43 | | | 0.01 | 2.94 | 0.05 | 0.06 |  |
|  | 1976-1985 | | 6 | | | 0.01 | *4.57 | -- | 0.06 | 2 |
|  | 1986-1993 | | 22 | | | 0.01 | 2.45 | 0.02 | 0.03 | 0.2 |
|  | 1994-2006 | | 15 | | | 0.01 | 2.45 | -- | 0.06 | 0.1 |
|  |  | |  | | |  |  |  |  |  |
| Craftsman | 1994-2006 | | 6 | | | 0.01 | 2.30 | -- |  | 0.1 |
|  |  | |  | | |  |  |  |  |  |
| Mechanical Maintenance | **overall** | | 22 | | | 0.01 | *4.17 | 0.10 | 0.17 |  |
|  | 1976-1985 | | 1 | | | 0.001 | -- | -- | 0.001 | 2 |
|  | 1986-1993 | | 12 | | | 0.01 | 3.43 | -- | 0.10 | 0.2 |
|  | 1994-2006 | | 9 | | | 0.02 | *4.67 | -- | 0.17 | 0.1 |
|  |  | |  | | |  |  |  |  |  |
| Boiler Cleaner | 1976-1985 | | 3 | | | 0.14 | -- | -- | 0.36 | 2 |
|  |  | |  | | |  |  |  |  |  |
| Oversight | **overall** | | 6 | | | 0.01 | 1.56 | -- | 0.02 |  |
|  | 1976-1985 | | 2 | | | 0.01 | -- | -- | 0.01 | 2 |
|  | 1986-1993 | | 3 | | | 0.01 | -- | -- | 0.02 | 0.2 |
|  | 1994-2006 | | 1 | | | 0.01 | -- | -- | 0.01 | 0.1 |

Empty (--) values were not calculated because the number of samples were deemed too low. Standard deviations were calculated for all samples with an n of 5 or greater. 95th percentiles were calculated for n of 20 or greater. *GSD greater than 3.5 suggests a high level of variability. If the maximum value is low, a high GSD is due to non-detects or extremely low values in the data group.

| **Table C10.** Geometric means and standard deviations for task sample airborne fiber concentrations in the Central Maintenance department grouped by job category (1972-2006) | | | | | | | | | |
| --- | --- | --- | --- | --- | --- | --- | --- | --- | --- |
|  |  |  | |  | **Asbestos Fiber Concentration by PCM (fibers/cc)** | | | | |
| **Job Title** | **Time Period** | | **n** | | **GM** | **GSD** | **95th percentile** | **Max** | **PEL** |
| Craftsman | **overall** | | 10 | | 0.14 | 2.30 | -- | 0.58 |  |
|  | 1972-1975 | | 1 | | 0.35 | -- | -- | 0.35 | 5 |
|  | 1976-1985 | | 9 | | 0.13 | 2.26 | -- | 0.58 | 2 |
|  |  | |  | |  |  |  |  |  |
| Mechanical Maintenance | **overall** | | 23 | | 0.02 | *12.08 | 0.88 | 0.94 |  |
|  | 1976-1985 | | 19 | | 0.03 | *15.06 | -- | 0.94 | 2 |
|  | 1986-1993 | | 4 | | 0.01 | -- | -- | 0.01 | 0.2 |
|  |  | |  | |  |  |  |  |  |
| Brake Repairman | 1976-1985 | | 2 | | 0.03 | *6.09 | -- | 0.09 | 5 |
|  |  | |  | |  |  |  |  |  |
| Electrician | 1976-1985 | | 7 | | 0.03 | *10.18 | -- | 0.38 | 2 |
|  |  | |  | |  |  |  |  |  |
| Insulator | 1976-1985 | | 2 | | 11.66 | -- | -- | 17.46 | 2 |
|  |  | |  | |  |  |  |  |  |
| Motor Inspector | **overall** | | 6 | | 0.34 | *4.46 | -- | 1.22 |  |
|  | 1976-1985 | | 5 | | 0.55 | 2.78 | -- | 1.22 | 2 |
|  | 1994-2006 | | 1 | | 0.03 | -- | -- | 0.03 | 0.1 |
|  |  | |  | |  |  |  |  |  |
| Oversight | 1976-1985 | | 3 | | 0.01 | -- | -- | 0.04 | 2 |

Empty (--) values were not calculated because the number of samples were deemed too low. Standard deviations were calculated for all samples with an n of 5 or greater. 95th percentiles were calculated for n of 20 or greater. *GSD greater than 3.5 suggests a high level of variability. If the maximum value is low, a high GSD is due to non-detects or extremely low values in the data group.

| **Table C11.** Geometric means and standard deviations for representative workday sample airborne fiber concentrations in the Central Maintenance department grouped by job category (1972-2006) | | | | | | | | | | |  |  |
| --- | --- | --- | --- | --- | --- | --- | --- | --- | --- | --- | --- | --- |
|  |  |  | |  | | **Asbestos Fiber Concentration by PCM (fibers/cc)** | | | | | | |
| **Job Title** | **Time Period** | | **n** | | **GM** | | **GSD** | **95th percentile** | **Max** | **PEL** | |  |
| Mechanical Maintenance | **overall** | | 24 | | 0.08 | | *7.46 | 0.70 | 1.10 |  | |  |
|  | 1972-1975 | | 15 | | 0.32 | | 2.17 | -- | 1.10 | 5 | |  |
|  | 1986-1993 | | 9 | | 0.32 | | 2.17 | -- | 0.03 | 0.2 | |  |
|  |  | |  | |  | |  |  |  |  | |  |
| Brake Repairman | **overall** | | 4 | | 0.01 | | -- | -- | 0.02 |  | |  |
|  | 1976-1985 | | 2 | | 0.01 | | -- | -- | 0.02 | 5 | |  |
|  | 1986-1993 | | 1 | | 0.02 | | -- | -- | 0.02 | 0.2 | |  |
|  | 1994-2006 | | 1 | | 0.01 | | -- | -- | 0.01 | 0.1 | |  |
|  |  | |  | |  | |  |  |  |  | |  |
| Insulator | 1976-1985 | | 3 | | 0.08 | | -- | -- | 0.09 | 2 | |  |
|  |  | |  | |  | |  |  |  |  | |  |
| Motor Inspector | 1976-1985 | | 1 | | 0.01 | | -- | -- | 0.01 | 2 | |  |

Empty (--) values were not calculated because the number of samples were deemed too low. Standard deviations were calculated for all samples with an n of 5 or greater. 95th percentiles were calculated for n of 20 or greater. *GSD greater than 3.5 suggests a high level of variability. If the maximum value is low, a high GSD is due to non-detects or extremely low values in the data group.

Empty (--) values were not calculated because the number of samples were deemed too low. Standard deviations were calculated for all samples with an n of 5 or greater. 95th percentiles were calculated for n of 20 or greater. *GSD greater than 3.5 suggests a high level of variability. If the maximum value is low, a high GSD is due to non-detects or extremely low values in the data group.

| **Table C12.** Geometric means and standard deviations for task sample airborne fiber concentrations in the Coke department grouped by job category (1976-1993) | | | | | | | | | | |
| --- | --- | --- | --- | --- | --- | --- | --- | --- | --- | --- |
|  |  |  | |  | | **Asbestos Fiber Concentration by PCM (fibers/cc)** | | | | |
| **Job Title** | **Time Period** | | **n** | | **GM** | | **GSD** | **95th percentile** | **Max** | **PEL** |
| Craftsman | 1976-1985 | | 23 | | 0.06 | | *11.03 | 1.01 | 2.30 | 2 |
|  |  | |  | |  | |  |  |  |  |
| CO Operator | 1976-1985 | | 1 | | 0.001 | | -- | -- | 0.001 | 2 |

Empty (--) values were not calculated because the number of samples were deemed too low. Standard deviations were calculated for all samples with an n of 5 or greater. 95th percentiles were calculated for n of 20 or greater. *GSD greater than 3.5 suggests a high level of variability. If the maximum value is low, a high GSD is due to non-detects or extremely low values in the data group.

| **Table C13.** Geometric means and standard deviations for representative workday sample airborne fiber concentrations in the Coke department grouped by job category (1976-1993) | | | | | | | | | | | |
| --- | --- | --- | --- | --- | --- | --- | --- | --- | --- | --- | --- |
|  |  |  | |  | | **Asbestos Fiber Concentration by PCM (fibers/cc)** | | | | | |
| **Job Title** | **Time Period** | | **n** | | **GM** | | **GSD** | **95th percentile** | **Max** | **PEL** |  |
| Craftsman | **overall** | | 28 | | 0.03 | | 3.04 | 0.13 | 0.17 |  |  |
|  | 1976-1985 | | 6 | | 0.05 | | 0.67 | -- | 0.17 | 2 |  |
|  | 1986-1993 | | 22 | | 0.02 | | 3.14 | 0.13 | 0.13 | 0.2 |  |
|  |  | |  | |  | |  |  |  |  |  |
| CO Operator | 1986-1993 | | 1 | | 0.16 | | -- | -- | 0.16 | 0.2 |  |
|  |  | |  | |  | |  |  |  |  |  |
| Insulator | 1986-1993 | | 2 | | 0.03 | | -- | -- | 0.05 | 0.2 |  |
|  |  | |  | |  | |  |  |  |  |  |
| Mechanical Maintenance | 1986-1993 | | 19 | | 0.01 | | 2.71 | -- | 0.05 | 0.2 |  |
|  |  | |  | |  | |  |  |  |  |  |
| Oversight | 1986-1993 | | 2 | | 0.04 | | -- | -- | 0.14 | 0.2 |  |

| **Table C14.** Geometric means and standard deviations for task sample airborne fiber concentrations in the Finishing department grouped by job category (1972-1993) | | | | | | | | | | | |  |
| --- | --- | --- | --- | --- | --- | --- | --- | --- | --- | --- | --- | --- |
|  |  |  | | | **Asbestos Fiber Concentration by PCM (fibers/cc)** | | | | | | | |
| **Job Title** | **Time Period** | | **n** | **GM** | | | **GSD** | **95th percentile** | | **Max** | **PEL** | |
| Finishing Operator | **overall** | | 5 | | 0.05 | *11.66 | | |  | 0.54 |  | |
|  | 1972-1975 | | 1 | | 0.21 | -- | | |  | 0.21 | 5 | |
|  | 1976-1985 | | 4 | | 0.04 | -- | | |  | 0.54 | 2 | |
|  |  | |  | |  |  | | |  |  |  | |
| Craftsman | 1976-1985 | | 1 | | 0.05 | -- | | |  | 0.05 | 2 | |
|  |  | |  | |  |  | | |  |  |  | |
| Motor Inspector | 1976-1985 | | 3 | | 0.22 | -- | | |  | 0.86 | 2 | |

Empty (--) values were not calculated because the number of samples were deemed too low. Standard deviations were calculated for all samples with an n of 5 or greater. 95th percentiles were calculated for n of 20 or greater. *GSD greater than 3.5 suggests a high level of variability. If the maximum value is low, a high GSD is due to non-detects or extremely low values in the data group.

| **Table C15.** Geometric means and standard deviations for representative workday sample airborne fiber concentrations in the Finishing department grouped by job category (1972-1993) | | | | | | | | | | | | | |
| --- | --- | --- | --- | --- | --- | --- | --- | --- | --- | --- | --- | --- | --- |
|  |  |  | | | **Asbestos Fiber Concentration by PCM (fibers/cc)** | | | | | | | | |
| **Job Title** | **Time Period** | | **n** | **GM** | | | **GSD** | **95th percentile** | | | **Max** | **PEL** | |
| Finishing Operator | **overall** | | 34 | | 0.02 | *7.12 | | | 1.11 | 1.20 | | |  |
|  | 1972-1975 | | 2 | | 0.36 | -- | | | -- | 0.87 | | | 5 |
|  | 1976-1985 | | 24 | | 0.03 | *7.01 | | | 1.11 | 1.20 | | | 2 |
|  | 1986-1993 | | 8 | | 0.01 | 2.09 | | | -- | 0.02 | | | 0.2 |
|  |  | |  | |  |  | | |  |  | | |  |
| Craftsman | **overall** | | 4 | | 0.01 | -- | | | -- | 0.01 | | |  |
|  | 1976-1985 | | 1 | | 0.01 | -- | | | -- | 0.01 | | | 2 |
|  | 1986-1993 | | 3 | | 0.01 | -- | | | -- | 0.01 | | | 0.2 |
|  |  | |  | |  |  | | |  |  | | |  |
| Crane Operator | **overall** | | 23 | | 0.01 | *5.83 | | | 0.13 | 1.12 | | |  |
|  | 1976-1985 | | 9 | | 0.01 | *8.49 | | | -- | 1.12 | | | 2 |
|  | 1986-1993 | | 14 | | 0.01 | *4.59 | | | -- | 0.13 | | | 0.2 |
|  |  | |  | |  |  | | |  |  | | |  |
| Mechanical Maintenance | **overall** | | 7 | | 0.09 | *12.5 | | | -- | 1.28 | | |  |
|  | 1976-1985 | | 5 | | 0.23 | *7.75 | | | -- | 1.28 | | | 2 |
|  | 1986-1993 | | 2 | | 0.01 | -- | | | -- | 0.04 | | | 0.2 |
|  |  | |  | |  |  | | |  |  | | |  |
| Motor Inspector | 1976-1985 | | 1 | | 0.01 | -- | | | -- | 0.01 | | | 2 |
|  |  | |  | |  |  | | |  |  | | |  |
| Oversight | 1976-1985 | | 4 | | 0.02 | -- | | | -- | 0.04 | | | 2 |
|  |  | |  | |  |  | | |  |  | | |  |
| Process Support | **overall** | | 22 | | 0.01 | *4.99 | | | 0.13 | 0.13 | | |  |
|  | 1976-1985 | | 7 | | 0.01 | *5.45 | | | -- | 0.05 | | | 2 |
|  | 1986-1993 | | 15 | | 0.01 | *5.04 | | | -- | 0.13 | | | 0.2 |

Empty (--) values were not calculated because the number of samples were deemed too low. Standard deviations were calculated for all samples with an n of 5 or greater. 95th percentiles were calculated for n of 20 or greater. *GSD greater than 3.5 suggests a high level of variability. If the maximum value is low, a high GSD is due to non-detects or extremely low values in the data group.

Empty (--) values were not calculated because the number of samples were deemed too low. Standard deviations were calculated for all samples with an n of 5 or greater. 95th percentiles were calculated for n of 20 or greater. *GSD greater than 3.5 suggests a high level of variability. If the maximum value is low, a high GSD is due to non-detects or extremely low values in the data group.

| **Table C16.** Geometric means and standard deviations for representative workday sample airborne fiber concentrations in the Foundry department grouped by job category (1972-1975) | | | | | | | | | | | |
| --- | --- | --- | --- | --- | --- | --- | --- | --- | --- | --- | --- |
|  |  |  | | **Asbestos Fiber Concentration by PCM (fibers/cc)** | | | | | | | |
| **Job Title** | **Time Period** | | **n** | | | **GM** | **GSD** | | **95th Percentile** | **Max** | **PEL** |
| Foundry Operator | 1972-1975 | | 1 | | 0.13 | | | -- | -- | 0.13 | 5 |
|  |  | |  | |  | | |  |  |  |  |
| Foundryman | 1972-1975 | | 9 | | 0.06 | | | 1.70 | -- | 0.10 | 5 |

| **Table C17.** Geometric means and standard deviations for task sample airborne fiber concentrations in the Masonry department grouped by job category (1972-1985) | | | | | | | | | | | | | | | |  |  |
| --- | --- | --- | --- | --- | --- | --- | --- | --- | --- | --- | --- | --- | --- | --- | --- | --- | --- |
|  | |  |  | |  | **Asbestos Fiber Concentration by PCM (fibers/cc)** | | | | | | | | | | | |
| **Job Title** | **Time Period** | | | **n** | | | | **GM** | | **GSD** | **95th percentile** | | **Max** | **PEL** | | | |
| Bricklayer | | **overall** | | 6 | | | 0.04 | | *7.41 | | | -- | 0.35 | |  | |  |
|  | | 1972-1975 | | 1 | | | 0.35 | | -- | | | -- | 0.35 | | 5 | |  |
|  | | 1976-1985 | | 5 | | | 0.03 | | *6.94 | | | -- | 0.12 | | 2 | |  |

Empty (--) values were not calculated because the number of samples were deemed too low. Standard deviations were calculated for all samples with an n of 5 or greater. 95th percentiles were calculated for n of 20 or greater. *GSD greater than 3.5 suggests a high level of variability. If the maximum value is low, a high GSD is due to non-detects or extremely low values in the data group.

| **Table C18.** Geometric means and standard deviations for representative workday sample airborne fiber concentrations in the Masonry department grouped by job category (1972-1985) | | | | | | | | | | | | | | | | |
| --- | --- | --- | --- | --- | --- | --- | --- | --- | --- | --- | --- | --- | --- | --- | --- | --- |
|  | |  |  | | |  | **Asbestos Fiber Concentration by PCM (fibers/cc)** | | | | | | | | | |
| **Job Title** | **Time Period** | | | | **n** | | | **GM** | | | **GSD** | **95th percentile** | | **Max** | **PEL** | |
| Bricklayer | | **Overall** | | 11 | | | | | 0.02 | *12.69 | | -- | 0.98 | | |  |
|  | | 1972-1975 | | 5 | | | | | 0.08 | *4.19 | | -- | 0.98 | | | 5 |
|  | | 1976-1985 | | 6 | | | | | 0.01 | *15.61 | | -- | 0.22 | | | 2 |

Empty (--) values were not calculated because the number of samples were deemed too low. Standard deviations were calculated for all samples with an n of 5 or greater. 95th percentiles were calculated for n of 20 or greater. *GSD greater than 3.5 suggests a high level of variability. If the maximum value is low, a high GSD is due to non-detects or extremely low values in the data group.

| **Table C19.** Geometric means and standard deviations for task sample airborne fiber concentrations in the Melt Shop department grouped by job category (1972-2006) | | | | | | | | | | |  |
| --- | --- | --- | --- | --- | --- | --- | --- | --- | --- | --- | --- |
|  |  |  | |  | **Asbestos Fiber Concentration by PCM (fibers/cc)** | | | | | | |
| **Job Title** | **Time Period** | | **n** | | | **GM** | **GSD** | **95th percentile** | **Max** | **PEL** | |
| Crane/Forklift Operator | 1972-1975 | | 5 | | | 0.37 | *4.04 | -- | 2.30 | 5 | |
|  |  | |  | | |  |  |  |  |  | |
| Mechanical Maintenance | 1976-1985 | | 1 | | | 0.001 | -- | -- | 0.001 | 2 | |
|  |  | |  | | |  |  |  |  |  | |
| Moldman | 1972-1975 | | 11 | | | 3.00 | *3.53 | -- | 23.80 | 5 | |
|  |  | |  | | |  |  |  |  |  | |
| Motor Inspector | 1976-1985 | | 2 | | | 0.45 | -- | -- | 1.04 | 2 | |
|  |  | |  | | |  |  |  |  |  | |
| Melt Shop Operator | 1976-1985 | | 3 | | | 0.01 | -- | -- | 0.03 | 2 | |
|  |  | |  | | |  |  |  |  |  | |
| Oversight | **overall** | | 2 | | | 0.001 | -- | -- | 0.001 |  | |
|  | 1976-1985 | | 1 | | | 0.001 | -- | -- | 0.001 | 2 | |
|  | 1994-2006 | | 1 | | | 0.001 | -- | -- | 0.001 | 0.1 | |

Empty (--) values were not calculated because the number of samples were deemed too low. Standard deviations were calculated for all samples with an n of 5 or greater. 95th percentiles were calculated for n of 20 or greater. *GSD greater than 3.5 suggests a high level of variability. If the maximum value is low, a high GSD is due to non-detects or extremely low values in the data group.

| **Table C20.** Geometric means and standard deviations for representative workday sample airborne fiber concentrations in the Melt Shop department grouped by job category (1972-2006) | | | | | | | | | | | |
| --- | --- | --- | --- | --- | --- | --- | --- | --- | --- | --- | --- |
|  |  |  | |  | **Asbestos Fiber Concentration by PCM (fibers/cc)** | | | | | | |
| **Job Title** | **Time Period** | | **n** | | | **GM** | **GSD** | **95th percentile** | **Max** | **PEL** |  |
| Crane/Forklift Operator | 1986-1993 | | 3 | | | 0.003 | -- | -- | 0.006 | 0.2 |  |
|  |  | |  | | |  |  |  |  |  |  |
| Mechanical Maintenance | **overall** | | 5 | | | 0.02 | *4.74 | -- | 0.08 |  |  |
|  | 1976-1985 | | 3 | | | 0.05 | -- | -- | 0.08 | 2 |  |
|  | 1986-1993 | | 2 | | | 0.004 | -- | -- | 0.02 | 0.2 |  |
|  |  | |  | | |  |  |  |  |  |  |
| Moldman | **overall** | | 12 | | | 1.43 | *6.09 | -- | 10.00 |  |  |
|  | 1972-1975 | | 11 | | | 2.24 | 2.59 | -- | 10.00 | 5 |  |
|  | 1976-1985 | | 1 | | | 0.01 | -- | -- | 0.01 | 0.2 |  |
|  |  | |  | | |  |  |  |  |  |  |
| Motor Inspector | 1976-1985 | | 2 | | | 0.79 | -- | -- | 1.36 | 2 |  |
|  |  | |  | | |  |  |  |  |  |  |
| Melt Shop Operator | 1976-1985 | | 2 | | | 0.10 | -- | -- | 0.96 | 2 |  |
|  |  | |  | | |  |  |  |  |  |  |
| Oversight | **overall** | | 7 | | | 0.008 | *5.91 | -- | 0.06 |  |  |
|  | 1976-1985 | | 4 | | | 0.002 | -- | -- | 0.004 | 2 |  |
|  | 1994-2006 | | 3 | | | 0.05 | -- | -- | 0.06 | 0.1 |  |

Empty (--) values were not calculated because the number of samples were deemed too low. Standard deviations were calculated for all samples with an n of 5 or greater. 95th percentiles were calculated for n of 20 or greater. *GSD greater than 3.5 suggests a high level of variability. If the maximum value is low, a high GSD is due to non-detects or extremely low values in the data group.

Empty (--) values were not calculated because the number of samples were deemed too low. Standard deviations were calculated for all samples with an n of 5 or greater. 95th percentiles were calculated for n of 20 or greater. *GSD greater than 3.5 suggests a high level of variability. If the maximum value is low, a high GSD is due to non-detects or extremely low values in the data group.

| **Table C21.** Geometric means and standard deviations for task sample airborne fiber concentrations in the Research Labs department grouped by job category (1972-2006) | | | | | | | | | | |
| --- | --- | --- | --- | --- | --- | --- | --- | --- | --- | --- |
|  |  |  | |  | **Asbestos Fiber Concentration by PCM (fibers/cc)** | | | | | |
| **Job Title** | **Time Period** | | **n** | | | **GM** | **GSD** | **95th percentile** | **Max** | **PEL** |
| Research Tester | 1976-1985 | | 2 | | | 0.50 | -- | -- | 0.51 | 2 |

Empty (--) values were not calculated because the number of samples were deemed too low. Standard deviations were calculated for all samples with an n of 5 or greater. 95th percentiles were calculated for n of 20 or greater. *GSD greater than 3.5 suggests a high level of variability. If the maximum value is low, a high GSD is due to non-detects or extremely low values in the data group.

| **Table C22.** Geometric means and standard deviations for representative workday sample airborne fiber concentrations in the Research Labs department grouped by job category (1972-2006) | | | | | | | | | | |
| --- | --- | --- | --- | --- | --- | --- | --- | --- | --- | --- |
|  |  |  | |  | **Asbestos Fiber Concentration by PCM (fibers/cc)** | | | | | |
| **Job Title** | **Time Period** | | **n** | | | **GM** | **GSD** | **95th percentile** | **Max** | **PEL** |
| Research Tester | **overall** | | 14 | | | 0.003 | 2.59 | -- | 0.02 |  |
|  | 1976-1985 | | 6 | | | 0.002 | 3.40 | -- | 0.02 | 2 |
|  | 1986-1993 | | 8 | | | 0.004 | 1.48 | -- | 0.01 | 0.2 |
| Janitor | 1986-1993 | | 4 | | | 0.01 | -- | -- | 0.01 | 0.2 |
